# Brazilian Society of Angiology and Vascular Surgery 2023 guidelines on the diabetic foot

**DOI:** 10.1590/1677-5449.202300872

**Published:** 2024-05-17

**Authors:** Eliud Garcia Duarte, Cicero Fidelis Lopes, Danilo Roberto Fadel Gaio, Jamil Victor de Oliveira Mariúba, Lorena de Oliveira Cerqueira, Marcos Antonio Bonacorso Manhanelli, Tulio Pinho Navarro, Aldemar Araújo Castro, Walter Jr. Boim de Araujo, Hermelinda Pedrosa, Júnio Galli, Nelson de Luccia, Clayton de Paula, Fernando Reis, Milton Sérgio Bohatch, Tércio Ferreira de Oliveira, Amanda Fernandes Vidal da Silva, Júlio Cesar Peclat de Oliveira, Edwaldo Édner Joviliano

**Affiliations:** 1 Hospital Estadual de Urgência e Emergência do Estado do Espírito Santo – HEUE, Departamento de Cirurgia Vascular, Vitória, ES, Brasil.; 2 Universidade Federal da Bahia – UFBA, Departamento de Cirurgia Vascular, Salvador, BA, Brasil.; 3 Faculdade de Engenharia de Sorocaba – FACENS, Sorocaba, SP, Brasil.; 4 Universidade Estadual Paulista “Júlio de Mesquita Filho” – UNESP, Departamento de Cirurgia Vascular, São Paulo, SP, Brasil.; 5 Universidade Vila Velha – UVV, Departamento de Cirurgia Vascular, Vila Velha, ES, Brasil.; 6 Conjunto Hospitalar de Sorocaba – CHS/Seconci, Departamento de Cirurgia Vascular, Sorocaba, SP, Brasil.; 7 Universidade Federal de Minas Gerais – UFMG, Faculdade de Medicina, Belo Horizonte, MG, Brasil.; 8 Universidade Estadual de Ciências da Saúde de Alagoas – UNCISAL, Departamento de Cirurgia Vascular, Maceió, AL, Brasil.; 9 Sociedade Brasileira de Angiologia e de Cirurgia Vascular – SBACV-PR, Curitiba, PR, Brasil.; 10 Universidade Federal do Paraná – UFPR, Hospital das Clínicas – HC, Curitiba, PR, Brasil.; 11 Hospital Regional de Taguatinga – HRT, Departamento de Cirurgia Vascular, Brasília, DF, Brasil.; 12 Universidade de São Paulo – USP, Faculdade de Medicina, Hospital das Clínicas – HC, São Paulo, SP, Brasil.; 13 Rede D’or São Luiz, Departamento de Cirurgia Vascular, São Paulo, SP, Brasil.; 14 Faculdade de Medicina de São José do Rio Preto – FAMERP, Hospital de Base, São José do Rio Preto, SP, Brasil.; 15 Sociedade Brasileira de Angiologia e de Cirurgia Vascular – SBACV-SP, São Paulo, SP, Brasil.; 16 Universidade Federal do Estado do Rio de Janeiro – UNIRIO, Departamento de Cirurgia Vascular, Rio de Janeiro, RJ, Brasil.; 17 Universidade de São Paulo – USP, Faculdade de Medicina de Ribeirão Preto – FMRP, Departamento de Cirurgia Vascular, Ribeirão Preto, SP, Brasil.

**Keywords:** diabetic foot, foot ulcer, diabetes mellitus

## Abstract

The diabetic foot interacts with anatomical, vascular, and neurological factors that challenge clinical practice. This study aimed to compile the primary scientific evidence based on a review of the main guidelines, in addition to articles published on the Embase, Lilacs, and PubMed platforms. The European Society of Cardiology system was used to develop recommendation classes and levels of evidence. The themes were divided into six chapters (Chapter 1 - Prevention of foot ulcers in people with diabetes; Chapter 2 - Pressure relief from foot ulcers in people with diabetes; Chapter 3 -Classifications of diabetic foot ulcers; Chapter 4 - Foot and peripheral artery disease; Chapter 5 - Infection and the diabetic foot; Chapter 6 - Charcot's neuroarthropathy). This version of the Diabetic Foot Guidelines presents essential recommendations for the prevention, diagnosis, treatment, and follow-up of patients with diabetic foot, offering an objective guide for medical practice.

## INTRODUCTION

Guidelines, an organized collection of medical information on a topic that is derived from quality scientific evidence, help doctors with diagnostic, therapeutic, and monitoring decisions for their patients.^1^ Understanding that such information requires constant updating to maintain its relevance and safety for specialists, in 2023 the Brazilian Society of Angiology and Vascular Surgery updated and incorporated new guidelines into its library. The objective is to provide a tool to assist in clinical decisions while preserving the doctor’s autonomy, as provided for in the Federal Council of Medicine’s Code of Medical Ethics.^2^

It is estimated that 415 million adults aged 20 to 79 years had diabetes mellitus (DM) worldwide in 2015, approximately 46.5% of whom lived in 3 countries: China (109 million), India (69 million) and the USA (29 million).^3^ Another 10-20 million lived in Brazil, Russia, and Mexico.^3^ According to International Diabetes Federation estimates, 9.1-26.1 million people with DM will develop diabetic foot ulcers (DFU) each year.^4^ Approximately 34% of people with DM will develop DFU at some point in their lives,^4^ with an annual risk of 2.5%-42% within 5 years.^5^ In addition to functional impairment and reduced quality of life, approximately 20% of patients with foot injuries will not heal 1 year after diagnosis,^6^ and the recurrence rate during this period is approximately 40%.^4^

**Figure 1:**
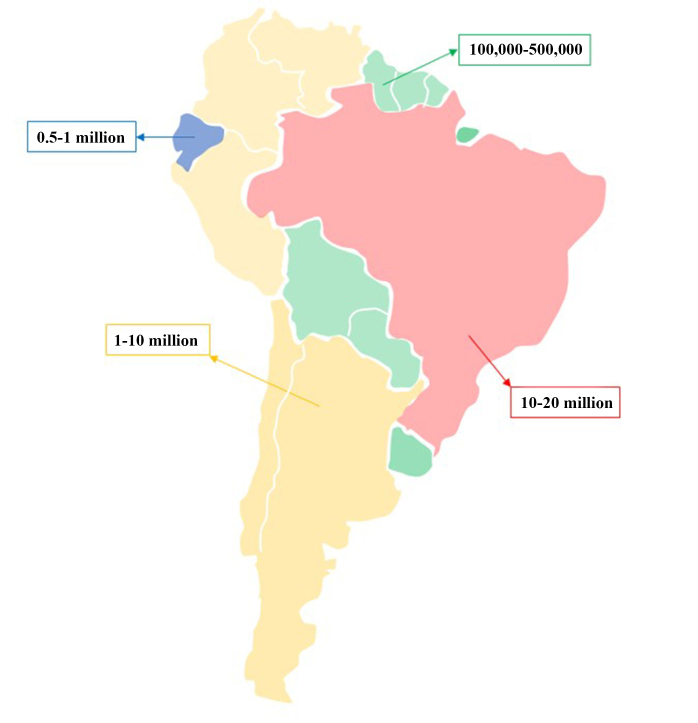
Estimated total number of adults (20-79 years) living with diabetes mellitus in South America.^3^

Having 13 million people diagnosed with DM, Brazil ranks fourth in worldwide prevalence and first in South America (Figure 1).^3,7^ Santos et al.^8^ analyzed diabetic foot complications in 27 Brazilian state capitals over a 10-year period (2008-2018), recording 45,095 complications. There was also a significant increase in complications between 2008 (5.68/100,000 inhabitants) and 2018 (17.68/100,000 inhabitants).

BRAZUPA, a cross-sectional study evaluating 1455 patients at 19 Brazilian centers, focused on risk factors for ulcers and amputation in patients with DM. Alarmingly, the mean age of the population with a limb at risk (57 years) was younger than that of either Western Europe or North America. Of the included patients, 18.6% had an active ulcer, 25.3% had a previous ulcer, and 13.7% had undergone an amputation.^9^

Diabetic foot corresponds to an interaction between anatomical, vascular (macro- and microangiopathy) and neurological factors, representing a complex challenge in the daily practice of vascular surgeons.^3,10^

## OBJECTIVES

This study compiles scientific evidence by reviewing the main guidelines and relevant articles, presenting important recommendations for the prevention, diagnosis, treatment, and follow-up of patients with diabetic foot and offering objective guidelines for medical practice.

## METHODOLOGY

Previously published guidelines were critically synthesized and the most relevant articles in the Embase, Lilacs, PubMed/MEDLINE, and Cochrane platforms were reviewed, which added important information for decision-making and recommendations. The main revised guidelines were: *The management of diabetic foot: A clinical practice guideline* by the Society for Vascular Surgery in collaboration with the American Podiatric Medical Association and the Society for Vascular Medicine (2016),^11^ *Best practice recommendations for the Prevention and Management of Diabetic Foot Ulcers* (2017),^12^ *Practical Guidelines on the prevention and management of diabetic foot disease* (2019),^13^ *Global vascular guidelines on the management of chronic limb-threatening ischemia* (2019),^14^ and *Australian evidence-based guidelines for diabetes-related foot disease* (2022).^15^

Only reviewed publications were included, following the “pyramid of evidence” principle (Tables 1 and 2). Multiple randomized clinical trials or meta-analyses of multiple randomized clinical trials were at the top of the pyramid, followed by single randomized clinical trials or large non-randomized studies (including meta-analyses of large cohorts), meta-analyses of small non-randomized studies, observational studies, and case series. Expert opinion was at the base of the pyramid, and case reports and summaries were excluded.

**Table 1:** Recommendation classes according to the European Society of Cardiology system.^16^

**Recommendation class**	**Definition**	**Suggestions for use**
Class I	Evidence and/or general acceptance that the treatment or procedure is beneficial, effective and applicable.	Recommended/indicated.
Class II	Conflict of evidence and/or differing opinions on the applicability/efficacy of the treatment or procedure.	
Class IIa	Relevant evidence is in favor of applicability/effectiveness.	Should be considered.
Class IIb	Applicability/efficacy is less established by evidence/opinion.	Can be considered.
Class III	Evidence and/or general acceptance that the treatment or procedure is not effective/applicable and may cause harm in certain cases.	Not recommended.

**Table 2:** Levels of evidence according to the European Society of Cardiology system.^16^

**Levels of evidence**	
Level A	Multiple randomized controlled trials or meta-analyses of randomized controlled trials.
Level B	Single randomized clinical trial or large non-randomized studies.
Level C	Expert opinion and/or small, retrospective studies or registries.

The European Society of Cardiology system was used to develop recommendation classes and levels of evidence.^16^ Strength (class) is graded from I to III, with I being the strongest (Table 1). Recommendations were developed by the authors assigned to each section, and all members approved the text and final classifications.

The main themes were divided into 6 chapters:

**Chapter 1** - Preventing foot ulcers in people with diabetes;
**Chapter 2** – Relieving pressure on foot ulcers in people with diabetes;
**Chapter 3** - Classifying diabetic foot ulcers;
**Chapter 4** – The diabetic foot and peripheral arterial disease;
**Chapter 5** - Infection and the diabetic foot;
**Chapter 6** - Charcot neuroarthropathy.

## CHAPTER 1. PREVENTING FOOT ULCERS IN PEOPLE WITH DIABETES

### Introduction

Diabetic foot is among the most serious complications of DM. It is a source of great suffering and financial cost, in addition to a considerable burden on the patient’s family, health care professionals and facilities, and society in general.^13^ It is estimated that in 2015 approximately USD 673 billion (12% of global health expenditure) was spent on treating DM and its complications.^3^

The frequency of amputation is much higher among people with diabetes than those without it.^17^ This is especially true in developed countries such as Canada, where adults with diabetes are 20 times more likely to be hospitalized for non-traumatic lower limb amputation than adults without diabetes.^18^ Preventive measures, foot care education, and early aggressive intervention are important components of diabetes treatment.

### Pathophysiology

Although the prevalence and spectrum of the diabetic foot vary in different regions of the world, the pathways of ulceration are similar in most patients. Peripheral neuropathy and peripheral arterial disease (PAD) often play a central role in diabetic foot complications, while infection often arises as a secondary phenomenon. However, all 3 components often have a synergistic role in the etiological triad. Peripheral neuropathy occurs in approximately 50% of diabetic patients, who often gradually develop “high pressure” zones in the foot that lead to loss of protective sensation (LPS), which is considered the main cause of DFU.^19,20^

**Figure 2:**
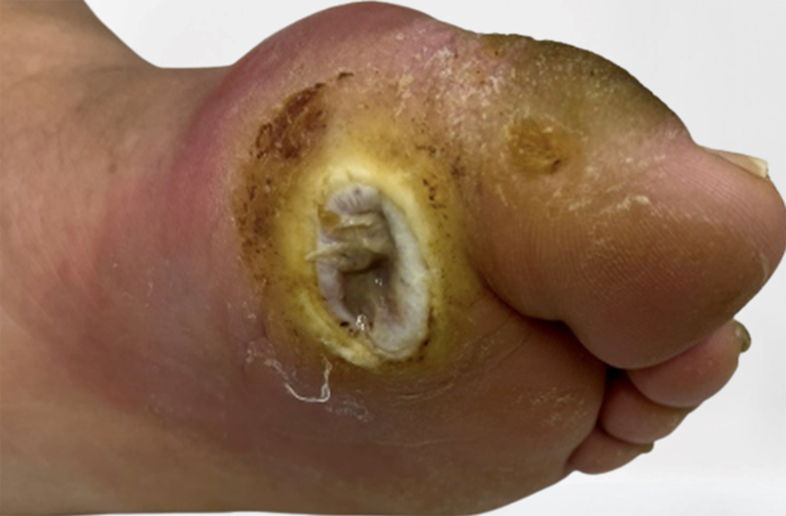
Plantar perforation due to mechanical stress.

In people with neuropathy, minor trauma (eg, improperly fitting shoes or acute mechanical or thermal injury) may precipitate foot ulceration. LPS, acquired foot deformities, and limited joint mobility can result in abnormal biomechanical loading on the foot. High mechanical stress in certain areas can lead to callus formation. The callus then leads to a further load increase on the foot, usually followed by subcutaneous hemorrhaging and, occasionally, skin ulceration. Finally, regardless of the primary cause of ulceration, continuing to walk with an insensitive foot impairs healing.^13,21^ People with diabetes are also more susceptible to infections due to neuropathy, PAD, microcirculation dysfunction, and immunopathy.^19^Figure 2 shows DFU resulting from biomechanical alterations to the foot, including callus formation and progression to ulcer and infection.

**Figure 3:**
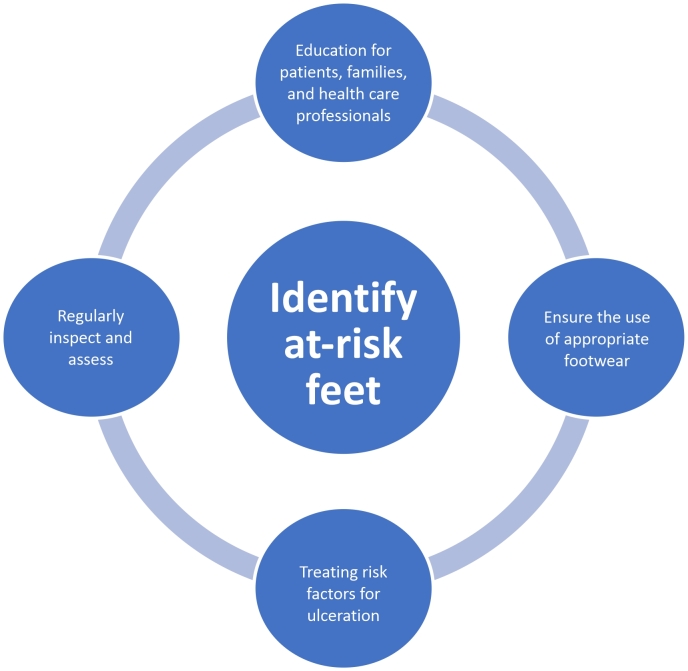
The pillars of diabetic foot ulcer prevention.^22^

There are 5 pillars to DFU prevention (Figure 3):

1. identifying the foot at risk;
2. regularly inspecting and examining the foot at risk;
3. educating patients, their families, and health care professionals;
4. ensuring the routine use of appropriate footwear;
5. treating risk factors for ulceration.

#### 1. Identifying the foot at risk

A lack of symptoms in people with diabetes does not exclude the disease; they may present asymptomatic neuropathy, PAD, pre-ulcerative signs, or an ulcer.^13,22^ A thorough examination of the feet is important for early disease detection. Screening for peripheral neuropathy and PAD can help identify patients at risk of foot ulcers (recommendation class I, level of evidence B). A history of ulcers or amputation and poor glycemic control increase the risk.^23,24^ Periodic reassessments are recommended for at-risk patients according to the risk stratification shown in Table 3 (recommendation class I, level of evidence C).

**Table 3:** IWGDF 2019 foot risk stratification and follow-up schedule.

**Category**	**Risk of ulceration**	**Characteristics**	**Frequency**
**0**	Very low	No LPS or PAD	Once per year
**1**	Low	LPS or PAD	Once every 6-12 months
**2**	Moderate	LPS + PAD or	Once every 3-6 months
		LPS + foot deformity or PAD + foot deformity	
**3**	High	LPS or PAD and:	Once every 1-3 months
		History of foot ulcer	
		Previous amputation	
		Terminal CKD	

LPS: loss of protective sensitivity; PAD: peripheral arterial disease; CKD: chronic kidney disease. Source: Bus et al.^22^

#### 2. Regularly inspecting and examining the foot at risk

**Figure 4:**
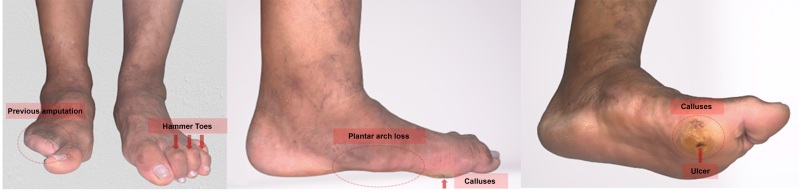
Examination findings in the diabetic foot. (A) History of previous amputation and hammer toes; (B) Plantar arch loss; (C) Calluses and ulcer.

The foot should be examined at each follow-up visit for active disease (ulceration or gangrene) (Figure 4A), as well as for lesions that increase the risk of ulceration, such as fungal infection, skin cracks and fissures, deformed nails, skin maceration, calluses and deformities (hammer toe, claw toe, and pes cavus) (Figure 4B and 4C) (recommendation class I, level of evidence B). Cold limb temperature may suggest ischemia, whereas flushing, increased warmth, or swelling may suggest inflammation, such as acute Charcot foot or cellulitis.^23,24^

Current American Diabetes Association and Canadian Diabetes Association guidelines recommend screening for diabetic neuropathy in all patients upon diagnosis of type 2 DM and after 5 years in patients with type 1 DM.^25^ Subsequent reevaluation should follow the International Working Group on the Diabetic Foot (IWGDF) risk stratification system.^22^

##### Risk stratification IWGDF 0

Patients should be assessed annually to identify risk factors for ulceration, such as PAD and neuropathy.^21,22^Chart 1 outlines basic clinical examination of these patients. In general, LPS is caused by diabetic neuropathy. If detected, it is generally necessary to obtain more information about the history of the disease and conduct additional tests regarding its causes and consequences (recommendation class I, level of evidence B).

**Chart 1:** Annual baseline assessment of very low risk patients (IWGDF 0).

Ø **History:**
• Previous ulcer/amputation; intermittent lameness/pain at rest.
Ø **Vascular examination:**
• Pulse palpation (pedis and posterior tibial).
Ø **Neurological examination:**
• Pressure perception (10 g monofilament);
• Vibration perception (128 Hz tuning fork);
Tactile sensitivity test (light touch for 1-2 seconds with the examiner's index finger on the tips of the toes).

IWGDF: International Working Group on the Diabetic Foot. Source: Bus et al.^22^

##### Risk stratification IWGDF ≥ 1

A more comprehensive examination must be performed in patients with PAD, LPS, deformities, a history of ulcer or amputation, or end-stage chronic kidney disease, as shown in Chart 2 (recommendation class I, level of evidence B).

**Chart 2:** Assessment of patients at higher risk (IWGDF ≥ 1).

Ø **Anamnesis:**
• Previous ulcer/amputation;
• End-stage kidney disease;
• Previous foot education/knowledge of foot care;
• Biopsyocosocial conditions;
• Access to the health system;
• Physical limitations that can impede self-care (visual acuity, obesity);
• Poor foot hygiene, for example, improper toenail trimming, dirty feet, fungal infection; dirty socks;
• Intermittent and resting claudication;
• Changes in sensitivity (allodynia, hyperalgesia, paresthesias).
Ø **Footwear type.**
Ø **Vascular examination:**
• Foot pulse palpation (pedis and posterior tibial).
Ø **Integumentary examination:**
• Skin color, temperature, presence of callus or edema, pre-ulcerative signs.
Ø **Bone/joint examination:**
• Deformities (claw or hammer toes);
• Abnormally large bony prominences;
• Limited joint mobility.
Ø **Neurological examination:**
• Pressure perception: 10 g Semmes-Weinstein monofilament;
• Vibration perception: 128 Hz tuning fork;
• Tactile sensation test: with the index finger, lightly touch the tips of the toes for 1-2 seconds.

IWGDF: International Working Group on the Diabetic Foot. Source: Bus et al.^22^

#### 3. Educating patients, their families, and health care professionals

Due to the lack of standardization and high heterogeneity of studies on self-care in foot ulcer prevention, no high-quality evidence on the effect of such interventions is available. However, they allow a person to detect the first signs of DFU, thus contributing to basic foot hygiene.^26^

Home monitoring of plantar foot temperature once a day with an infrared thermometer can be considered a preventive intervention, especially when high temperatures are observed for 2 consecutive days (recommendation class IIb, level of evidence B).^27-29^ However, despite its easy applicability, the results may unnecessarily worry people and lower their confidence, and calibrated equipment is required.^27-29^

Pre-ulcerative signs, such as blisters, fissures, or calluses with subcutaneous hemorrhaging, ingrown toenails, or onychomycosis appear to be strong predictors of foot ulceration.^30^ A health care professional should remove abundant calluses, protect blisters (draining them when necessary), and treat fissures, ingrown toenails, and fungal infections (recommendation class I, level of evidence C).^31,32^ Flexor tenotomy may be considered for patients with an ulcer or pre-ulcerative sign in the toe who do not respond to conservative treatment and who require normalization of the foot structure to prevent ulceration (recommendation class IIb, level of evidence C).^33,34^ Because these treatments may cause harm when performed inappropriately, they should only be performed by professional, adequately trained health care providers.^31,32^

Chart 3 lists the main care recommendations for patients with diabetic foot.^22^

**Chart 3:** Main recommendations for patients with diabetic foot ulcers.

**Education for patients, families, and health care professionals**
Balanced diet, rich in fiber, low in saturated fats and sugars.
Appropriate nail trimming (straight; avoid removing cuticles).
Avoid walking barefoot or wearing inappropriate shoes.
Daily assessment of the feet (family help is necessary for cases of visual impairment or disability).
Wash feet daily (water temperature < 37 °C), drying carefully between the toes.
Moisturize feet well (not between the toes).
To avoid burning the feet, do not use hot water bottles.
Do not attempt to remove calluses.
Have feet regularly evaluated by health care professionals.
Notify health care professionals if there are blisters, cuts, scrapes, ulcerations, or temperature increases.
Routinely consult an ophthalmologist, nutritionist, and an endocrinologist.
Adopt safety measures to control falls and mitigate risks inherent to proprioception changes.

Source: Bus et al.^22^

#### 4. Ensuring the use of appropriate footwear

All of the patient’s footwear must be clinically assessed for the following characteristics:^12^

- fit: the toe space must be large enough to avoid pressure and the heel must be firm, but not too tight;
- structure: shoes must have laces/Velcro fasteners; they must not have seams or structures that could result in friction or pressure;
- cushioning;
- general characteristics: shoes must be made of breathable materials, such as leather, to allow moisture to dissipate;
- movement control: shoes must limit excessive pronation (everted foot and arch flattening);
- other: clinicians must confirm that there are no foreign objects inside the shoe;
- never use footwear that has previously caused ulceration.

The footwear of people at moderate or high risk of ulceration (IWGDF risk 2-3) must be adjusted to protect and accommodate the shape of the toes, including adequate length, width, and depth. This may require custom-made shoes, insoles, or orthotics to reduce the risk of ulceration or recurrence (recommendation class I, level of evidence B).^35-37^

**Figure 5:**
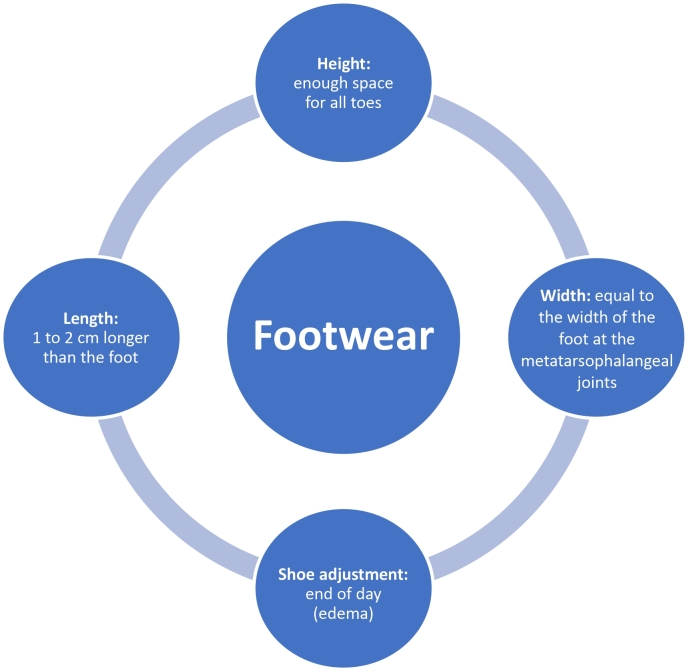
Features of suitable footwear.^22^

The benefit of continuously using custom molded shoes or insoles with proven pressure relief outweigh the potential harm.^38,39^ However, footwear with inadequate length or width increases the risk of ulceration, so a proper fit must be ensured.^18^The characteristics of suitable footwear are summarized in Figure 5. A risk stratification protocol for footwear selection is shown in Chart 4.

**Chart 4:** Risk stratification for footwear selection.

**IWGDF 0**	Appropriate footwear: ready for use off the rack.
**IWGDF 1-3**	Appropriate footwear: custom made or selected.

IWGDF: International Working Group on the Diabetic Foot. Source: Bus et al.^22^

#### 5. Treating risk factors for ulceration

Any modifiable risk factor or pre-ulcerative sign must be treated. Treatment must be repeated until these anomalies disappear and do not recur. In patients who have recurrent ulcers due to foot deformities despite adhering to the above mentioned preventive measures, surgical intervention should be considered.^22^Chart 5 summarizes the main measures for controlling the risk of ulceration. Table 4 summarizes the main recommendations from the latest IWGDF consensus.^22^

**Chart 5:** Summary of measures to control the risk of ulceration.

**Measures to control the risk of ulceration**
Treat excess calluses.
Protect or adequately drain blisters if necessary.
Adequately treat onychodystrophy and onychomycosis.
Perform surgeries to restructure foot biomechanics, especially if there are recurrent ulcerations.
Notify health care professionals if there are blisters, cuts, scrapes, ulcerations, or temperature increases.
Routinely consult an ophthalmologist, nutritionist, and endocrinologist.
Adopt safety measures to control falls and mitigate risks inherent to proprioception changes.

Source: Bus et al.^22^

**Table 4:** Recommendations of the International Working Group on the Diabetic Foot (IWGDF), 2019.

**Recommendations**	**Recommendation class and level of evidence**
1. Examine a person with diabetes at very low risk for foot ulceration annually (IWGDF risk 0) for signs or symptoms of loss of protective sensation and peripheral arterial disease to determine whether they are at increased risk for foot ulceration.	Class I/level of evidence B.^23^
2. Screen a person with diabetes at risk of foot ulceration (IWGDF risk 1-3) for: history of foot ulceration or lower limb amputation, diagnosis of end-stage kidney disease, presence or progression of foot deformity, limited joint mobility, abundant callus, and any pre-ulcerative sign on the foot.	Class I/level of evidence B.^23^
3. Repeat screening once every 6-12 months for those classified as IWGDF risk 1, once every 3-6 months for those classified as IWGDF risk 2, and once every 1-3 months for those classified as IWGDF risk 3.	Class I/level of evidence C.^22^
4. Instruct diabetic patients at risk of foot ulceration (IWGDF risk 1-3) to protect their feet by avoiding walking shoeless or in flip-flops, whether indoors or outdoors. Educate them and encourage self-care.	Class I/level of evidence C.^12^
5. Instruct/encourage/remind diabetic patients at risk of ulceration (IWGDF risk 1-3) to: inspect the entire surface of both feet and inside the shoes to be worn daily; wash their feet daily (carefully drying them, especially between the toes); use moisturizers to hydrate dry skin; cut their toenails straight; and avoid using chemical agents, plasters, or any other technique to remove corns or calluses.	Class I/level of evidence B.^26^
6. Consider instructing diabetic patients at moderate or high risk of foot ulceration (IWGDF risk 2-3) to self-monitor foot skin temperature once a day to identify early signs of inflammation and help prevent ulceration. If the temperature difference is above the threshold for similar regions on both feet on 2 consecutive days, instruct the patient to walk less and consult a health care professional.	Class IIb/level of evidence B.^27-29^
7. To reduce plantar pressure and avoid foot ulceration, instruct diabetic patients at moderate risk for foot ulceration (IWGDF risk 2) or with a healed non-plantar ulcer (IWGDF risk 3) to wear therapeutic footwear that conforms to the shape of the foot and fits properly. When a foot deformity or pre-ulcerative sign is present, consider prescribing custom-made shoes, insoles, or toe orthotics.	Class I/level of evidence B.^35,36^
7. Consider prescribing orthopedic interventions, such as silicone toe orthoses or (semi-)rigid orthopedic devices, to help reduce heavy calluses in diabetic patients at risk of foot ulceration (IWGDF risk 1-3).	Class IIa/level of evidence B.^37^
8. For diabetic patients with a healed plantar ulcer (IWGDF risk 3), prescribe therapeutic footwear with proven effects on weight-bearing during walking to help prevent a recurrent plantar foot ulcer; encourage patients to wear these shoes at all times.	Class I/level of evidence B.^38,39^
9. Provide appropriate treatment for any pre-ulcerative signs or profuse calluses on the foot, ingrown toenails, or fungal infections to help prevent a foot ulcer in diabetic patients at risk of foot ulceration (IWGDF risk 1-3).	Class I/level of evidence C.^31,32^
10. Consider digital flexor tendon tenotomy to prevent ulceration in diabetic patients with a profuse callus or ulcer on the top or distal region of a nonrigid hammertoe that has not healed with nonsurgical treatment.	Class IIb/level of evidence C.^33,34^
11. In diabetic patients with a forefoot plantar ulcer that has not healed with nonsurgical treatment, consider Achilles tendon lengthening, joint arthroplasty, uni- or panresection of the metatarsal heads, metatarsophalangeal joint arthroplasty, or osteotomy to help prevent forefoot plantar ulcer recurrence once the active ulcer has healed.	Class IIb/level of evidence C.^40-42^
12. To help prevent a foot ulcer in diabetic patients at moderate or high risk of foot ulceration (IWGDF risk 2-3) who have neuropathic pain, nerve decompression is not suggested as a replacement for standard care.	Class III/level of evidence C.^22^
13. Consider advising diabetic patients at low or moderate risk of foot ulceration (IWGDF risk 1 or 2) to perform foot mobility exercises to reduce ulceration risk factors, ie, decreasing plantar pressure and increasing the foot and ankle’s range of motion to improve neuropathy symptoms.	Class IIa/level of evidence B.^43-46^
14. Advise diabetic patients (IWGDF risk 1 or 2) to get daily exercise, such as walking (ie, 1000 extra steps/day). Advise them to wear appropriate footwear when performing load-bearing activities and monitor the skin frequently for pre-ulcerative signs or skin breakdown.	Class IIb/level of evidence B.^47,48^

Adapted from Bus et al.^22^

## CHAPTER 2. RELIEVING PRESSURE ON FOOT ULCERS IN PEOPLE WITH DIABETES

Pressure relief can be achieved with temporary footwear until the ulcer heals and the foot tissues stabilize. Removable or non-removable pressure relief boots can effectively reduce pressure on ulcers in the plantar surface.^20,49,50^

Although pressure relief casts effectively support the healing of non-infected nonischemic neuropathic plantar ulcers, it is necessary to carefully select patients and personnel with specialized training to minimize the risk of iatrogenic complications.^51^

When a bony deformity of the foot prevents the use of appropriate footwear or relief of pressure-related ulcers, consultation with a foot and ankle surgeon may be considered to evaluate and treat the deformity.^52,53^Flowchart 1 summarizes pressure relief treatment. Table 5 summarizes the main recommendations from the latest IWGDF consensus.^13^

**Flowchart 1:**
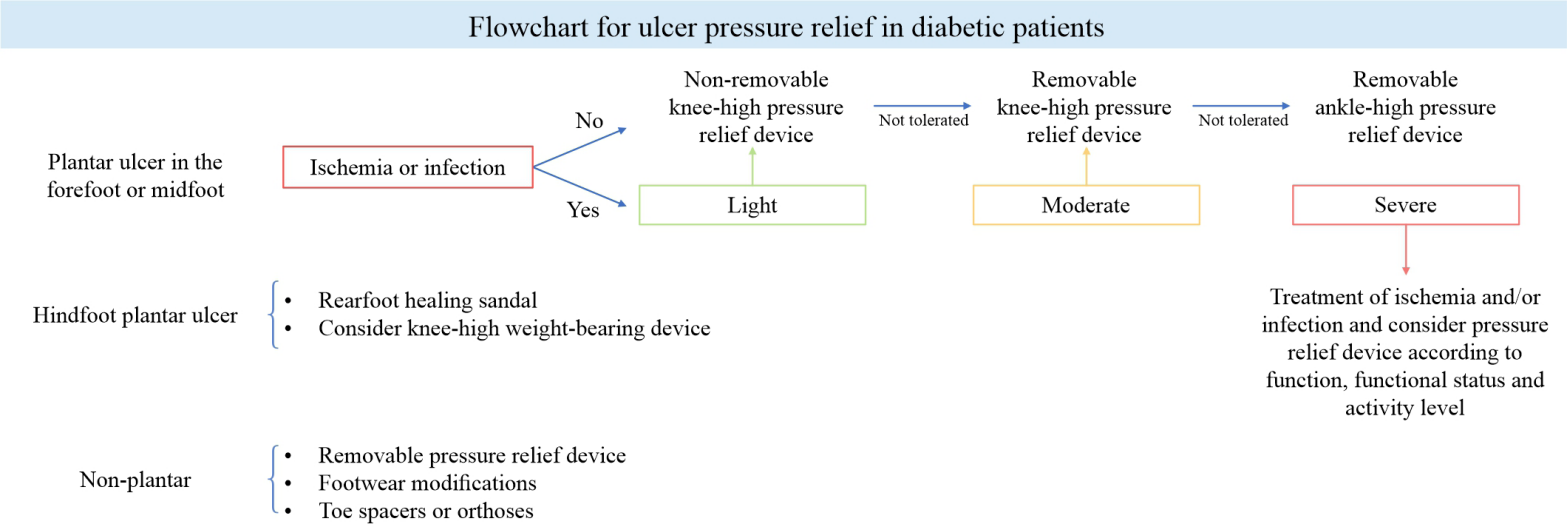
Practical summary of pressure relief measures. Source: Schaper et al.^13^

**Table 5:** Main recommendations from the latest consensus of the International Working Group on the Diabetic Foot.

**Recommendations**	**Recommendation class and level of evidence**
1. For neuropathic plantar ulcers of the forefoot or midfoot, use a knee-high non-removable pressure relief device (full cast or weight-bearing walking boot with added plaster or an adhesive bandage around the boot) with a foot interface as the first choice for pressure relief treatment. Non-removable weight-bearing devices help heal diabetic foot ulcers by redistributing pressure in the foot and lower leg and through forced adherence.	Class I/level of evidence A.^51,54-58^
2. For neuropathic forefoot/midfoot ulcers, use a full cast, or a removable weight-bearing walking boot that has been made non-removable; the choice will depend on available resources, the skills of the involved technicians, patient preference, and the extent of the foot deformity.	Class I/level of evidence A.^51,55,59^
3. For neuropathic plantar ulcers in the forefoot or midfoot in which a non-removable knee-high pressure relief device is contraindicated or not tolerated, consider using a removable knee-high weight-bearing walking boot with a foot interface device as a second choice for pressure relief treatment.	Class IIb/level of evidence B.^60,61^
4. For neuropathic plantar ulcers in the forefoot or midfoot where a knee-high weight-bearing boot is contraindicated or not tolerated, use a removable ankle-high pressure relief device as a third treatment option.	Class IIa/level of evidence C.^62-66^
5. For neuropathic plantar ulcers in the forefoot or midfoot, instruct patients not to use prefabricated therapeutic footwear or conventional footwear as a pressure relief treatment unless none of the above mentioned devices are available.	Class IIb / level of evidence B.^55,66-68^
6. In item 5, consider using properly fitted felted foam in combination with therapeutic footwear or conventional footwear as a fourth option for pressure relief.	Class IIb/level of evidence C.^69-71^
7. For neuropathic plantar ulcers of the forefoot or midfoot with mild infection or ischemia, consider using a knee-high non-removable pressure relief device.	Class IIb / level of evidence C.^55,72,73^
8. For neuropathic plantar ulcers of the forefoot or midfoot with mild or moderate infection and ischemia, consider using a removable knee-high pressure relief device.	Class IIb/level of evidence C.^73^
9. For neuropathic plantar ulcers in the forefoot or midfoot with moderate or severe infection or ischemia, primarily address the infection and/or ischemia and consider using a removable pressure relief device that improves patient functionality.	Class IIb/level of evidence C.^55,72,73^
10. For neuropathic heel ulcers, consider using a knee pressure relief device or other intervention that effectively reduces plantar heel pressure and can be tolerated by the patient.	Class IIa/level of evidence B.^74,75^
11. For non-plantar ulcers, use a removable ankle pressure relief device, shoe modifications, toe spacers, or orthotics, depending on the type and location of the ulcer.	Class I/level of evidence B.^74^

Adapted from Schaper et al.^13^

## CHAPTER 3. CLASSIFYING DIABETIC FOOT ULCERS

### Introduction

Due to the complexity of factors involved in DFU, there is still no classification system for routine clinical use that encompasses the diverse populations the world.^13,76^ In a review, Monteiro-Soares et al.^76^ found 37 classifications for DFU. In part, this wide variety is due to different purposes, eg, clinical care, research, and auditing. Clinical care, which concerns limbs and injuries in individual patients, aims to standardize communication between health professionals, establish prognosis, and guide therapeutic approaches. Research and auditing, however, are concerned with limbs and injuries in groups of patients.^13,76,77^ Currently, there is no classification/scoring system for analyzing individual prognosis in people with DFU.^13^

### Clinical practice

A consensus classification, scoring system, and description of foot injuries in routine clinical practice would facilitate decision-making and communication between professionals.^77^ Descriptive classifications separate patients into groups but do not necessarily establish prognoses. Scoring systems assign scores to the factors involved in the disease and generally serve to estimate severity and adverse outcomes.^13,77^

The system used in routine clinical practice, including communication between multidisciplinary health teams, must be simple enough to easily memorize and apply, and it should not require any specialized equipment.^13,77^ The IWGDF recommends the Site, Ischemia, Neuropathy, Bacterial Infection, and Depth (SINBAD) system (recommendation class I, level of evidence B).^13^

There are only 2 classification systems to aid clinical decision making: IWGDF/IDSA and the Wound, Ischemia, and foot Infection (WIfI) system. Current guidelines recommend WIfI, although the IWGDF/IDSA can be used alone if the equipment required for the WIfI system is unavailable (recommendation class IIb, level of evidence B).^13,14,77^

### Clinical research

The purpose of a classification system is to identify clinical characteristics for the inclusion or exclusion of patients in studies. Because this is usually done on an experimental basis, only participating centers must agree on the criteria and descriptions.^77^ The Perfusion, Extent, Depth, Infection and Sensation (PEDIS) classification system has refined definitions for prospective research projects, emphasizing reliance on 5 specified criteria: area; depth; infection; neuropathy; and ischemia.^77^

### Clinical auditing

The reasons for auditing can range from a simple description of patient numbers to a search for associations between diseases and outcomes to comparative results between different institutions. These comparisons are essential for optimized clinical management. Because the groups studied in each case can be large, any classification or scoring system must be simple, unambiguous, easily understood, clearly documented without expensive equipment, and accurate enough to be meaningful.^77^ SINBAD is currently the best validated system for auditing and is recommended by the IWGDF (recommendation class IIa, level of evidence C).^13,77^Flowchart 2 summarizes DFU classifications according to study objectives.

**Flowchart 2:**
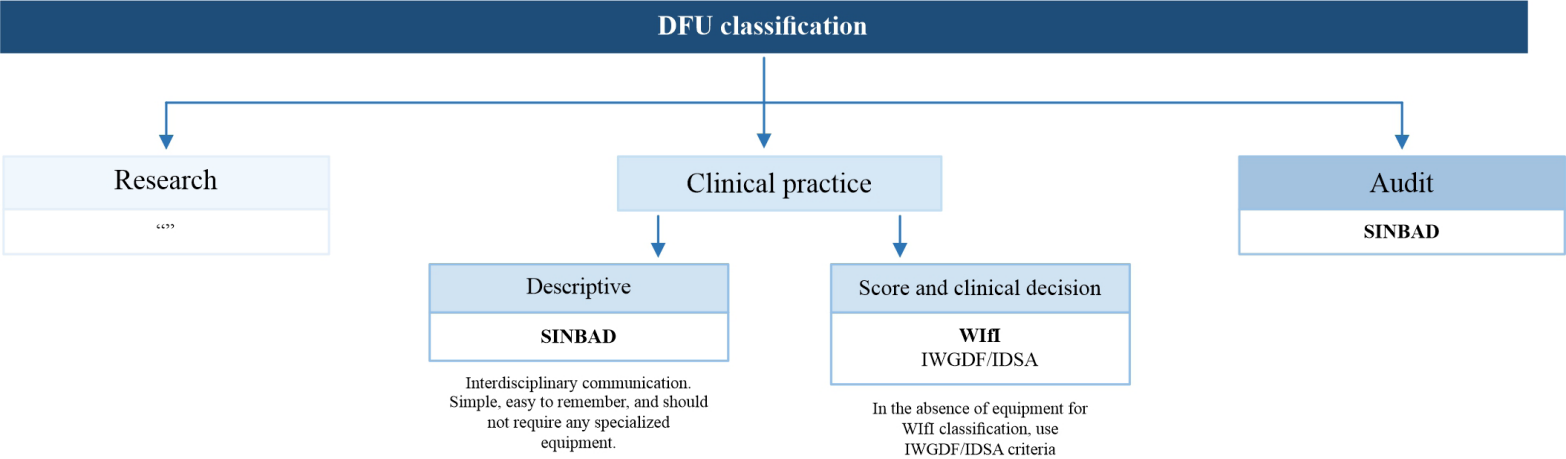
Classificações das DFU conforme o objetivo a ser estudado. DFU: diabetic foot ulcer; IWGDF: International Working Group on the Diabetic Foot; IDSA: Infectious Diseases Society of America; SINBAD: Site, Ischemia, Neuropathy, Bacterial Infection, and Depth; WIfI: wound, ischemia, and foot infection classification system. Source: Game^77^ and Schaper et al.^13^

### Rating systems

#### SINBAD classification

The SINBAD system (described in Table 6) is simple, quick, and easy to use, requiring no specialized equipment other than clinical examination, and contains the necessary information to allow screening by a specialized team. The total score is obtained by summing the points for all important ulcer-related data (scored as 0 or 1). The 6 DFU domains are: area, depth, presence of sepsis, PAD, denervation, and location. Total scores can reach 6 points. This system has been validated for predicting ulcer healing and amputations.^78-80^ SINBAD is the most widely validated system in different research contexts and has broadly consistent results.^13^

**Table 6:** SINBAD classification system.

**Category**	**Definition**	**Score**
1. Local	Forefoot	0
	Midfoot or rearfoot	1
2. Ischemia	Preserved flow	0
	Missing flow	1
3. Neuropathy	Preserved protective sensitivity	0
	Absent protective sensitivity	1
4. Infection	Absent	0
	Present	1
5. Area	Ulcer < 1 cm^2^	0
	Ulcer ≥1 cm^2^	1
6. Depth	Skin and subcutaneous involvement	0
	Muscle, tendon, or deeper involvement	1
Maximum score		6

Adapted from Schaper et al.^13^

#### Infectious Diseases Society of America classification

The Infectious Diseases Society of America (IDSA) guidelines were developed for scientific purposes as part of the PEDIS classification system and to identify patients in need of hospital admission for antibiotic therapy. They were later validated for assessing the risk of major and minor amputation. The IDSA system consists of 4 severity levels for DFU and infection, as shown in Table 7.^13^

**Table 7:** Infectious Diseases Society of America classification system.

**Clinical picture**	**PEDIS score**	**Infection severity**
Ulcer without exudate or inflammation.	1	Not infected
At least 2 manifestations of inflammation (exudate or erythema, mild pain, heat or induration), cellulitis/erythema extends around the ulcer and the infection is limited to the skin or subcutaneous tissue; no other local complications or systemic disease.	2	Light
Signs of infection (as above) in metabolically stable patients without toxemia who have ≥ 1 of the following features: cellulitis extending > 2 cm, disseminated lymphangitic streaking beneath the superficial fascia, deep tissue abscess, gangrene, and involvement of muscle, tendon, joint, or bone.	3	Moderate
Infection in patients with toxemia or metabolic instability (eg, fever, chills, tachycardia, hypotension, confusion, vomiting, leukocytosis, acidosis, severe hyperglycemia, or azotemia).	4	Severe

Adapted from Schaper et al.^13^

#### WIfI System

Published in 2014, the WIfI (wound, ischemia, and foot infection) system was designed to classify at-risk limbs. It stratifies the risk of major amputation within 1 year and predicts whether revascularization will be necessary for wound healing and limb salvage.^81^ The Global Vascular Guidelines, published in 2019, recommend using the WIfI classification analogously to the Malignant Tumor Classification system for cancer staging to analyze the at-risk limb.^14^ It is the classification of choice for patients with DFU according to the latest IWGDF guidelines, which were also published in 2019.^13^ WIfI has been validated on several continents, including in specific groups with DFU.^82-84^ It has been used in large multicenter trials, such as BEST-CLI, BEST-CLI2, BASIL-2, and BASIL-3, and has high levels of interobserver and intraobserver reproducibility.^85^

The system’s purpose is to provide a more accurate description of the main factors that lead to non-healing and limb loss, as well as to assist in decision-making in clinical practice.^76^ WIfI does not require quantifying the wound area or determining the presence of neuropathy, but specific equipment is needed to measure pressures and ischemic indices.^76,77^

The system consists of 3 components: wound extent, the presence of ischemia, and the degree of foot infection. Wound extent is evaluated using improved criteria from the University of Texas classification, which, depending on the degree, also correlates with the expected type of surgery. The foot infection classification system, which is the same as that used by the IDSA, predicts amputation risk and has been validated for DFUs. The degree of ischemia is determined using pressures and indices to objectively assess foot perfusion and healing potential without revascularization.^80^ Through a Delphi consensus, the Society for Vascular Surgery committee responsible for the Global Vascular Guidelines on the Management of Chronic Limb-threatening Ischemia created a score for foot wounds, ischemia, and infection in relation to the 1-year risk of limb amputation (very low, low, moderate, or high), assigning a risk of intervention for each possible combinations of scores.^14,76^Tables 8, 9 and 10 detail the WIfI classification system.

**Table 8:** Wound, ischemia, and foot infection (WIfI) classification system.

**Ulcer score**	**Diabetic foot ulcer**	**Gangrene**
0	None	None
	Clinical description: ischemic pain at rest*	
1	Small, shallow ulcer on the distal part of the leg or foot; no bones exposed unless limited to the distal phalanx.	None
	Clinical description: small tissue loss. Recoverable with simple amputation (1 or 2 digits) or skin coverage.	
2	Deeper ulcer with exposed bone, joint, or tendon that generally does not involve the heel; shallow heel ulcer without involvement of the calcaneus.	Limited to toes
	Clinical description: major loss of salvageable tissue requiring multiple digital (≥ 3) or standard transmetatarsal amputations with standard skin coverage.	
3	Extensive and deep ulcer involving the forefoot and/or midfoot; deep heel ulcer with or without involvement of the calcaneus.	Extensive: involving the forefoot or midfoot; heel necrosis ± calcaneal involvement.
	Clinical description: extensive tissue loss recoverable only with complex foot reconstruction or non-traditional transmetatarsal amputation (Chopart or Lisfranc); flap coverage or complex management of the ulcer due to extensive soft tissue loss.	

* Ischemic pain at rest affects the forefoot; it is often worse while reclining but relieved when the limb is hanging; it lasts > 2 weeks and must be associated with ≥ 1 abnormal hemodynamic parameters (ankle-brachial index < 0.4; ankle systolic pressure < 50 mm Hg; toe systolic pressure <30 mmHg; PtcO_2_ < 30 mm Hg, and flat or minimal pulsatile waves). Source: Conte et al.^14^ and Schaper et al.^13^

**Table 9:** Wound, ischemia, and foot infection (WIfI) classification for ischemia.

**Degree**	**ABI**	**TSP, PtcO_2_**
0	≥ 0.8	≥ 60 mmHg
1	0.6-0.79	40-59 mmHg
2	0.4-0.59	30-39 mmHg
3	≤ 0.39	< 30 mmHg

ABI: ankle-brachial index; TSP: toe systolic pressure; PtcO_2_: transcutaneous oxygen pressure.

Measure TSP or Ptco2 if the ABI is incompressible (> 1.3). TSP must be measured in diabetic patients and prevails over ABI when they have discordant values. If arterial calcification prevents reliable examination of ABI or TSP, ischemia should be assessed by PtcO_2_, skin perfusion pressure, or pulse volume recording. If ankle systolic pressure and ABI measurements result in different grades, ankle systolic pressure will be the primary determinant of ischemia degree. Source: Conte et al.^14^ and Schaper et al.^13^

**Table 10:** Wound, ischemia, and foot infection (WIfI) classification for foot infection

**SVS**	**Clinical manifestation of infection**	**IDSA/PEDIS Infection degree**
0	No signs or symptoms of infection	Not infected
1	Infection present, defined by the presence of ≥ 2 of the following:	Minimal
	1.Swelling or local induration;	
	2. Erythema > 0.5 to ≤ 2 cm around the ulcer;	
	3.Local sensitivity or pain;	
	4.Local heat;	
	5. Purulent secretion (thick, opaque-to-white or bloody secretion).	
	Local infection involving only the skin and subcutaneous tissue (no involvement of deeper tissues and no SIRS.	
	Exclude other causes of skin inflammatory response (trauma, gout, acute Charcot neuropathic osteoarthropathy, fracture, thrombosis, venous stasis).	
2	Local infection (described above) with erythema > 2 cm or involving structures deeper than the skin and subcutaneous tissues (eg, abscess, osteomyelitis, septic arthritis, fasciitis) and without signs of SIRS.	Moderate
3	Local infection (described above) with signs of SIRS, manifested by ≥ 2 of the following:	Severe*
	1. Temperature > 38 ºC or < 36 ºC;	
	2. Heart rate > 90 beats/min;	
	3. Respiratory rate > 20 breaths/min or PaCO_2_ < 32 mm Hg;	
	4 Leukocyte count > 12,000 or < 4000 cells/mm^3^ or 10% immature forms.	

IDSA: Infectious Diseases Society of America; PaCO_2_: partial pressure of arterial carbon dioxide; PEDIS: perfusion, extent, depth, infection and sensation; SIRS: systemic inflammatory response syndrome; SVS: Society for Vascular Surgery.

* Ischemia can complicate and increase the severity of any infection. Systemic infection may present with other clinical findings, such as hypotension, confusion, and vomiting, or evidence of metabolic disturbances, such as acidosis, severe hyperglycemia, or new-onset azotemia. Source: Conte et al.^14^ and Schaper et al.^13^

#### PEDIS classification

PEDIS was developed as a descriptive classification system for research, aiming to define DFU and facilitate communication between health services – but not for prognostic purposes. It does not include patient characteristics or the location or number of ulcers. Although mainly designed for research, its use in clinical practice and auditing is not ruled out. PEDIS classifies diabetic foot ulcers into 5 categories of impairment: perfusion, extension, tissue depth/loss, infection, and sensitivity (described in Table 11).^76,77^

**Table 11:** Perfusion, extent, depth, infection and sensation (PEDIS) classification system.

**Category**	**Definition**
1. Perfusion	Degree 1: no signs or symptoms of PAD;
	Degree 2: signs or symptoms of PAD, but no critical ischemia;
	Degree 3: critical ischemia.
2. Extent	Measurement in centimeters.
3. Depth	Degree 1: small superficial ulcer that does not penetrate below the dermis;
	Degree 2: deep ulcer, below the dermis that compromises subcutaneous structures and involves fascia, muscle, and tendon;
	Degree 3: all parts of the foot involved, including bones and/or joints (bone exposure, bone involvement).
4. Infection	Degree 1: no signs or symptoms of infection;
	Degree 2: infection involving the skin and subcutaneous cellular tissue, no deep tissue involvement or SIRS;
	Degree 3: erythema > 2 cm and ≥ 1 of the following: edema, local heat, erythema or infection involving deeper structures such as abscess, osteomyelitis, septic arthritis, fasciitis. No SIRS;
	Degree 4: foot infection with SIRS:
	1.Temperature > 38 °C or < 36 °C;
	2. Heart rate > 90 beats per minute;
	3. Respiratory rate > 20 breaths per minute or PaCO_2_ < 32 mmHg;
	5. Leukocyte count > 12,000/mm or > 10% immature forms of leukocytes.
5. Sensation	Degree 1: no loss of sensation in the affected foot;
	Degree 2: loss of sensation in the affected foot according to objective tests:
	- 10 g monofilament test;
	- Vibratory sensitivity test.

PAD: peripheral arterial disease; PaCO_2_: partial pressure of arterial CO_2;_ SIRS: systemic inflammatory response syndrome. Source: Peters and Lavery.^86^

Table 12 summarizes the recommendations of international guidelines about the use of classification systems.

**Table 12:** Main recommendations from the latest consensus of the International Working Group on the Diabetic Foot (IWGDF).

Recommendations	Recommendation class and level of evidence
1. Use the SINBAD system to classify diabetic foot ulcers and communicate between multidisciplinary health teams.	Class I/level of evidence B^14,87-90^
2. Do not use any classification system to offer an individual prognosis for patients with diabetic foot ulcers.	Class III/level of evidence C^13^
3. Use the IWGDF/IDSA or WIfI system in clinical practice to guide decision making.	Class IIb/level of evidence B^82-84,91,92^
4. Use the SINBAD system for regional/national/international auditing since it allows comparison of diabetic foot ulcer results between institutions.	Class IIa/level of evidence C^78,80^

IDSA: Infectious Diseases Society of America; SINBAD: Site, Ischemia, Neuropathy, Bacterial Infection, and Depth; WIfI: Wound, ischemia and foot infection. Adapted from Schaper et al.^13^

## CHAPTER 4. THE DIABETIC FOOT AND PERIPHERAL ARTERIAL DISEASE

### Introduction

Diabetes is a strong risk factor for PAD, including the development of more diffuse, multisegmental, and predominantly distal arterial disease. These factors increase the complexity of treatment and are associated with a worse prognosis.^93^

### Epidemiology

PAD is highly prevalent in diabetic patients, affecting approximately 25% of those aged > 60 years.^94^ Stoberock et al.^95^ found that the prevalence of PAD varies from 20%-50% and 10%-26% in people with and without diabetes, respectively. The coexistence of DM and PAD (ankle-brachial index [ABI] < 0.9) is associated with a 2- to 4-fold increase in mortality.^96^

PAD affects up to 50% of patients with DFU and is associated with poor prognosis, such as amputation (5-24%), ulcer persistence (10-15%), ulcer recurrence, increased hospital admissions, reduced quality of life, and increased mortality.^97-100^ Patients with DFU and PAD have an estimated 5-year mortality of 50%. This grim prognosis is largely attributable to the systemic nature of arterial disease. Furthermore, patients with ischemic and neuroischemic DFU have a higher risk of all-cause mortality than diabetic patients without DFU or with neuropathic DFU.^95,101^

### Pathophysiology and risk factors for peripheral arterial disease in diabetic patients

PAD develops in diabetes through a complex interaction of hemodynamic, metabolic, and neurohormonal factors, which, through different mechanisms, produce endothelial and smooth muscle cell dysfunction, abnormalities in hemostasis and blood viscosity, chronic inflammation, accumulation of glycation end-products, and oxidative stress.^102,103^ In order of importance, the main risk factors for PAD are smoking, DM, arterial hypertension, and hypercholesterolemia. However, other associations have been described, particularly in DM patients, such as disease duration, high levels of glycosylated hemoglobin, abdominal obesity, male sex, and neuropathy.^98^

### Macro- and microvascular manifestations in diabetic patients

Macrovascular manifestation of atherosclerotic disease in DM is generally bilateral and tends to involve arteries in the infrapopliteal segment. Concomitant femoropopliteal involvement is also common and has the same incidence in the non-diabetic population, while involvement of the iliac segment, especially in isolation, is less frequent.^13,103^ It is also common for diabetic patients to have an incomplete plantar arch and a higher risk of PAD, including a palpable tibial pulse, which can evolve into ulcerations and gangrene in the toes.^13^ The lesions have multisegmental characteristics with long occlusions, a reduced collateral network, and extensive arterial calcification.^13,101^ They affect younger individuals, presenting a rapid clinical course with greater tissue loss and risk of amputation, and have a high recurrence rate after revascularization.^13,103,104^

The microvascular system includes capillaries and arterioles (up to ≈100 um) and is essential for maintaining tissue homeostasis, providing oxygen and nutrients for wound healing. It allows angiogenesis and hormonal signaling and participates in the regulation of systemic blood pressure.^99,105^ Microvascular dysfunction is characterized by an imbalance between blood flow and vascular tone, resulting in compromised oxygen supply to tissue, increased oxidative stress, impaired healing, and target organ damage.^105^ Among the main microvascular complications (polyneuropathy, nephropathy, and retinopathy), retinopathy appears to have a greater correlation with wound healing failure, minor amputation, and mortality.^99^ Thus, microvascular disease has been proposed as a risk factor for both PAD progression and amputation.^99^

Although some authors assert that these microangiopathy mechanisms affect healing, no concrete evidence currently supports such a hypothesis, which prevents extrapolation to clinical practice. Therefore, PAD continues to be the most important cause of perfusion deficit in diabetic patients, and microvascular disease should not preclude recommending limb revascularization.^13^

### Diagnosis

The diagnosis, prognosis, and treatment of diabetic patients with PAD are markedly different from those without PAD. No clinical signs or symptoms accurately exclude PAD in patients with DM.^97^ Approximately 50% of patients are asymptomatic,10% have symptoms of intermittent claudication, and PAD can remain subclinical until patients experience severe tissue loss.^13,97^ The lack of symptoms may be related to polyneuropathy and loss of pain sensitivity.^13^ Early identification of PAD is essential in patients with DFU, since arterial involvement is associated with a greater risk of ulcers that do not heal, infection, and amputation, as well as increased cardiovascular morbidity and mortality.^13^ Ischemic ulceration generally affects the forefoot and toes, but other areas may be affected in patients with diabetic neuropathy, altered biomechanics, or foot deformities. Therefore, all patients presenting signs or symptoms of PAD should undergo complete vascular assessment.^14^

Anamnesis should be directed towards PAD risk factors, such as hypertension, dyslipidemia, smoking, obesity, DM duration, and manifestations of atherosclerotic disease, such as cerebrovascular and coronary disease. Patients who have had diabetes > 10 years are more prone to PAD.^98^ Microvascular disease mainly affects the retinal vessels (eg, amaurosis), glomerular vessels (eg, renal failure), and those that nourish peripheral nerves (vasa nervosarum), causing sensory, motor, and autonomic polyneuropathy.^106^

Ischemic pain at rest affects the forefoot and often increases with recumbency, although it is relieved when the limb is hanging. It lasts > 2 weeks and must be associated with one or more altered hemodynamic parameters (ABI < 0.4; ankle systolic pressure [ASP] < 50 mmHg; toe systolic pressure [TSP] < 30 mmHg; transcutaneous oxygen pressure [PtcO_2_ ] < 30 mmHg and flat or minimal pulse waves). Pressure measurements must be correlated with arterial Doppler waveforms due to medial calcinosis.^14^

All patients suspected of PAD should undergo a thorough physical examination. Although non-specific, characteristics such as coldness, skin xerosis, muscular atrophy, and rarified or dystrophic nails are frequently observed in patients with PAD.^14,98^ However, autonomic and motor polyneuropathy can manifest with some of these integumentary and muscle changes, respectively. The foot’s temperature may be relatively warm due to dysautonomia or arteriovenous shunts, which mask the signs of an ischemic limb.^13^ Patients with suspected PAD should not be examined while sitting in a chair with their leg hanging, since this may lead to false interpretations regarding foot perfusion.^14^ Buerger’s sign, pallor in an elevated foot, redness in a hanging one, and a capillary refill time > 5 seconds are also signs of a limb with arterial insufficiency.^14^

Although foot pulse palpation is part of the initial workup, the results should not be used in isolation to exclude PAD in patients with DM. Even when assessed by experienced professionals, a palpable pulse may be present in limbs with significant ischemia.^13^ A systematic review demonstrated that foot pulse palpation had a sensitivity of 55% and specificity of 60% for PAD diagnosis.^97^ Therefore, a more objective assessment should be performed in all patients with DFU.^13^

Bedside diagnostic tests may lose accuracy due to peripheral neuropathy, arterial calcification, or peripheral edema.^13^ There is still no ideal test or defined cut-off value that safely excludes PAD. It is recommended to use more than 1 test in parallel to increase diagnostic accuracy.^13,97^ In most patients with DFU, the Doppler wave shape of the distal arteries should be assessed in combination with ASP and ABI or with toe systolic pressure (TSP) measurement and the toe/brachial index (TBI). PAD is less likely with an ABI of 0.9-1.3, TBI ≥ 0.75, and a triphasic Doppler waveform (recommendation class I, level of evidence C). However, if there is uncertainty or an unfavorable clinical course, the investigation should be complemented with imaging exams.^13^ Alternative tests that may also be useful when investigating DFU include: pulse oximetry, pulse volume, photoplethysmography, transcutaneous oxygen tension, and skin perfusion pressure.^13,14^

### Ankle-brachial index

Peripheral (autonomic) neuropathy associated with calcification of the tunica media (Mönckeberg sclerosis) in the distal arteries makes them rigid and incompressible, and can even result in a falsely elevated ABI (between 0.4 and 1.4). This phenomenon should be suspected when the ABI is near or within normal range, but is associated with abnormal (damped) monophasic waveforms, which can be recognized acoustically or visually on a monitor. False-normal ASP and ABI values have been reported as independent predictors of major amputation.^13,14^ Detection of a triphasic arterial waveform with a handheld Doppler ultrasound device appears to provide stronger evidence for ruling out PAD. Although an ABI < 0.9 is useful for detecting PAD, results > 0.9 do not exclude PAD.^13,97^

ABI may be more useful for diagnosing PAD in patients with intact feet, but it is less useful for ruling out PAD in patients with foot neuropathy or DFU (sensitivity 69.5% vs 80.7%; specificity 74% vs 91.5%). Thus, it should not be used in isolation to rule out PAD in DFU patients.^97^

### Toe systolic pressure and toe/brachial index

Digital arteries are often spared the extensive calcification that occurs in tibial arteries, so their flow measurement more accurately reflects foot perfusion in people with DM.^14^ TSP measurement is recommended whenever falsely elevated ASP or ABI are detected or suspected, especially when the values do not agree with acoustic or visual analysis of the waveform.^13,14,97^

TSP is measured using a specific cuff that is placed around the base of the great toe and is connected to a standard pressure gauge. A photoplethysmographic or continuous wave Doppler flow detector is then used to determine the flow return after cuff deflation.^14^ TSP is generally 20-40 mmHg less than ASP. TBI < 0.7 is considered abnormal, and TSP < 30 mm Hg is associated with advanced ischemia.^14^ TSP < 50 mmHg has excellent diagnostic ability in patients with DFU, but normal TSP has not been considered accurate enough to rule out diagnosis.^97^ PAD is unlikely if the TBI is ≥ 0.75. TBI and waveform analysis in the tibial arteries (measured at the medial malleolus, dorsum of the foot, and mid-calf for the peroneal artery) are the most useful non-invasive screening tests for patients who require additional diagnostic imaging.^13^

### Audible handheld Doppler: analysis

A validation study to determine the usefulness of audible handheld Doppler ultrasound^107^ examined 200 patients (379 legs). The ABI and TSP of all patients was measured in certified vascular laboratories. Audible handheld Doppler signals were sufficiently sensitive to rule out PAD (98.6% posterior tibial, 97.8% dorsalis pedis), but not sufficiently specific to diagnose it (37.5% posterior tibial, 30.19% dorsalis pedis). The test is simple, quick, and can serve as an alternative to ABI. Audible handheld Doppler results (ABI > 0.9) are identified as biphasic or triphasic. If a monophasic waves or no sound is detected, duplex Doppler ultrasound of the entire limb should be ordered.^12^

### Transcutaneous oxygen pressure

PtcO_2_ allows microvascular assessment and can also reflect the perfusion of large and small vessels.^108^ The sensitivity of PtcO_2_ appears to be better than ASP (82% vs 67%) in intact feet.^109^ However, the sensitivity of both PtcO_2_ and ASP is reduced in patients with DFU (28% vs 47%, respectively), having low diagnostic value in such cases.^110^ However, it should be noted that PtcO_2_ decreases only when reductions in macrovascular arterial perfusion are so critical that they reduce tissue oxygen supply.^108^ This phenomenon occurs through microvascular compensatory mechanisms of hyperemia in ischemic limbs, maintaining a curvilinear relationship between PtcO_2_ values and local perfusion pressures sufficient to maintain normal tissue oxygenation.^108^

### Pulse oximetry

Pulse oximetry is an attractive technique due to its low cost and device availability in most health care settings, although its applicability is still limited by a lack of scientific evidence about PAD diagnosis in patients with DFU. The measurement considers toe saturation < 2% less than finger saturation or a toe saturation increase > 2% when the leg is elevated 12 inches above the horizontal plane.^97^

Overall, there is not yet enough evidence to recommend a single bedside test to reliably rule out PAD in patients with DFU. Normal ABI (or a palpable pulse) cannot reliably rule out PAD. A second test should be performed, such as Doppler waveform assessment, possibly in combination with TSP and TBI measurement. Pulse oximetry could become an attractive alternative if confirmed in future studies.^97^ Bedside tests for probable PAD in diabetic feet are summarized below:^97^

1. ABI < 0.9 can suggest PAD, but values between 0.9 and 1.3 do not exclude PAD, especially in patients with neuropathy and/or DFU;
2. TBI > 0.75 makes PAD diagnosis less likely;
3. Pulse oximetry results (ie, if toe saturation is < 2% less than finger saturation or increased by > 2% when the leg is elevated 12 inches above the horizontal plane) may suggest PAD or make it less likely;
4. Analysis of the tibial waveform (triphasic/monophasic) can be useful in PAD diagnosis.

Several other noninvasive tests, including laser Doppler flowmetry, skin perfusion pressure, and plethysmography, have been used to assess limb perfusion. However, these tests can be influenced by a variety of confounding factors and are not routinely used in most vascular laboratories around the world.^14^

### Assessing prognosis of ischemic diabetic foot ulcers

Due to the scarce literature, comorbidities in patients with DFU, and the complexity of arterial lesions (infrapopliteal predominance, extensive calcification, reduced collateral network, and long lesions), there is still no single measure to consistently predict healing.^13^ Diagnostic tests, the WIfI system, and disorder duration can help physicians decide about more detailed arterial study and the need for revascularization (recommendation class I, level of evidence B). Above all, decisions must always be considered in light of the patient’s comorbidities.^13,14^

### Prognostic value of bedside tests

Indicators of a greater probability of healing in patients with PAD and DFU include skin perfusion pressure ≥ 40 mmHg, TSP ≥ 30 mmHg, and PtcO_2_ ≥ 25 mmHg (recommendation class I, level of evidence B). Any of these findings increases the probability of healing by at least 25%.^13^ Indicators of a low probability of healing and an increased risk of amputation are ASP < 50 mmHg, ABI < 0.5, TSP < 30 mmHg, and PtcO_2_ < 25 mmHg. Although ABI has little value in healing prognosis, subnormal values are associated with a higher risk of amputation. In these patients, imaging tests are recommended and early revascularization should be considered.^13,97^

### WIfI classification system

The WIfI system was developed through expert consensus and has since been validated in populations with and without diabetes.^13,14,81^ This classification system for foot ulcers, ischemia, and infection, which helps estimate the risk of amputation and the potential benefit of limb revascularization, is recommended by international guidelines.^13,14^ The system is detailed in Chapter 3.

### Clinical course

Regardless of the diagnostic test results, imaging studies are recommended and revascularization should be considered in all patients with DFU and PAD if the ulcer does not shrink by approximately 50% within 4-6 weeks, even with ideal treatment (adequate infection control, wound care, and offloading) and no other probable cause of poor healing (recommendation class IIa, level of evidence C).^13,14^

### Comorbidities

Healing is related to the interaction of perfusion deficit with other characteristics of the patient and the foot, such as tissue loss, infection, mechanical load on the ulcer, and comorbidities (eg, heart failure or end-stage renal disease). Clinical stability and metabolic and infection control are fundamental for the regenerative process.^13^ Imaging exams and urgent treatment should also be considered in patients with PAD (even with higher pressure levels) when there are other predictors of poor prognosis, such as infection or extensive ulceration.^13^

### Arterial imaging tests

High-quality arterial imaging is essential for determining the best method of limb revascularization. Anatomical information from the arterial bed must be obtained to assess the severity and distribution of arterial stenoses or occlusions. Detailed study of the infrapopliteal arteries and feet is essential for patients with DFU.^13,14^

#### Color duplex ultrasound

It is recommended to begin the investigation with color duplex ultrasound due to its accessibility, non-invasive nature, and low cost, in addition to using no iodinated contrast medium, no ionizing radiation, and the device’s portability.^13,14^ Arterial circulation in the lower limb can be assessed directly and completely, from the abdomen to the foot. This method provides anatomical detail and a physiological assessment of blood flow, determining the location and extent of the disease, as well as providing information about flow speed and volume. Diffuse multisegmental involvement, extensive calcification, edema, and tissue loss can hamper the quality of the examination.^13,14^ Its main disadvantages are the delay required to perform the examination, its high operator dependence, the fact that it does not produce a map of the arterial bed, and its limited estimation of collateral arterial supply.^14^

#### Computed tomography angiography and nuclear magnetic resonance imaging

Computed tomography (CT) angiography has high sensitivity and specificity in the aortoiliac (95% and 96%, respectively) and femoropopliteal (97% and 94%, respectively) segments. Its sensitivity and specificity fall slightly near the infrapopliteal region (95% and 91%, respectively), especially in cases of extensive calcification, which can make it difficult to evaluate smaller arteries.^111^ Other disadvantages include allergic reactions, contrast-induced nephropathy, and the use of ionizing radiation.^13,14^

One of the main advantages of nuclear magnetic resonance imaging (MRI) angiography is its use of a contrast agent with low nephrotoxicity (gadolinium) and no ionizing radiation. Disadvantages include stenosis overestimation, difficulty assessing in-stent restenosis, compatibility issues with implanted devices (pacemakers and defibrillators), long image acquisition times, and image artifacts. Its use is limited in patients with claustrophobia, as well as those with severe renal failure (creatinine clearance < 30 mL/min) due to the risk of nephrogenic systemic fibrosis. New non-gadolinic agents, such as ultrasmall paramagnetic iron oxide particles, may be safer in patients with compromised renal function.^13,14^

To assess arterial disease in leg and foot vessels, neither CT nor nuclear MRI angiography produce complete images with sufficient resolution for therapeutic planning. Thus, the 2019 Global Vascular Guidelines on the Management of Chronic Limb-threatening Ischemia, which have been endorsed by the Society for Vascular Surgery, the European Society for Vascular Surgery, and the World Federation of Vascular Societies, do not recommend CT angiography for detailed study of infrapopliteal disease, which must be investigated by complete diagnostic angiography, including the ankle and foot.^13,14^

#### Digital subtraction angiography

Digital subtraction angiography is still considered the gold standard for arterial imaging due to its high spatial resolution, especially for the infrapopliteal territory. Its advantages include allowing treatment during the procedure, while its disadvantages include the use of iodinated contrast and its invasiveness, which can result in complications related to arterial puncture.^13,14^ CO_2_ angiography can be used in patients allergic to iodinated contrast and in those with severe chronic kidney disease. Its image quality is lower than iodinated angiography and the image progressively degrades along the leg, although it can still provide useful diagnostic images and it reduces the volume of iodinated contrast.^13,14^Flowchart 3 summarizes the use of imaging tests in patients with a revascularization plan.

**Flowchart 3:**
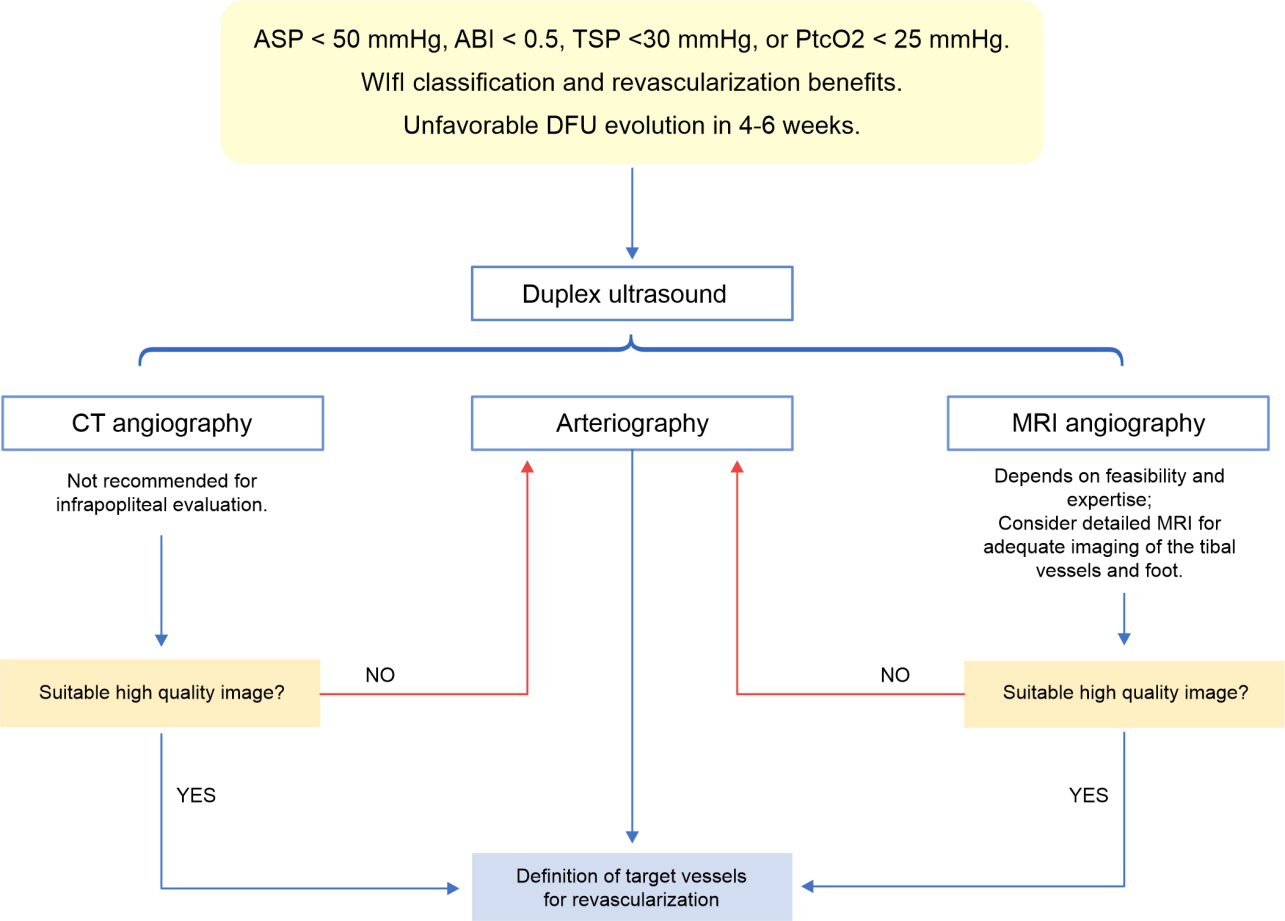
Use of imaging exams in patients with a revascularization plan. ASP = ankle systolic pressure; ABI = ankle-brachial index; CT: computed tomography; DFU = diabetic foot ulcer; MRI: magnetic resonance imaging; PtcO_2_ = transcutaneous oxygen pressure; TSP = toe systolic pressure; WIfI: wound, ischemia, and foot infection classification system. Source: Conte et al.,^14^ Forsythe et al.^97^ and Schaper et al.^13^

### Limb revascularization

The indications for PAD treatment are similar in diabetic and non-diabetic patients: limiting claudication, reducing pain at rest, and reducing tissue loss associated with non-healing ulcers and gangrene.^14^ Approximately 25% of patients with ischemic DFU have no revascularization options, and the major amputation rate due to unsuccessful limb revascularization or to being ineligible for revascularization is 25%-50%.^101,112^ These individuals are generally characterized by multilevel arterial disease, including high involvement of the arteries of the foot (approximately 75% of cases).^112^ Among diabetic patients, Faglia et al.^113^ found that the lack of a patent tibial artery at the end of angioplasty resulted in a 62% amputation rate, compared to 1.7% among patients with at least 1 patent artery to the foot.

The goal of revascularization is to restore direct blood flow to at least 1 artery in the foot, preferably one supplying the anatomical region of the ulcer. Perfusion is the main parameter for DFU healing, amputation level selection, and limb salvage. It should be noted that a delay of more than 2 weeks from DFU diagnosis to revascularization substantially increases the risk of limb loss.^114^ However, revascularization must be considered on a case-by-case basis, since the ulcers can heal in up to 50% of patients with DFU and PAD who do not undergo revascularization.^13^

Once PAD has been diagnosed, the need for revascularization will be based on the PLAN concept, in which: P = patient risk, L = limb threat severity (WIfI classification), and AN = anatomic pattern of disease, ie, assessing the extent of arterial disease according to the Global Anatomic Staging System. PLAN assists in treatment selection, from primary amputation to revascularization, and helps determine the best revascularization option (open or endovascular surgery).^14^

A systematic review found that the limb salvage rate ranges from 70-90% for patients who undergo revascularization (either open or endovascular), with more than 60% of ulcers healed within 1 year.^115^ Even in patients with unfavorable arterial anatomy who undergo ultradistal bypass or inframalleolar angioplasty, limb salvage rates at 1 year have been reported at 86% and 77%.^101^

Based on current evidence, no technique (endovascular, open, or hybrid) can be considered superior to another. Furthermore, no large randomized studies have determined the most appropriate revascularization methods specifically for patients with DFU and PAD.^101^ Decisions must consider individual factors, such as the morphological distribution of PAD, autogenous vein availability, comorbidities, and surgeon expertise (recommendation class I, level of evidence B).^13,14,101^

The Bypass vs Angioplasty in Severe Ischemia of the Leg (BASIL)^116^ study compared endovascular intervention with open surgery. Perioperative morbidity was higher in the surgery group, but overall and 1-year amputation-free survival were similar between groups. However, at 2 years, the surgery group was associated with a lower risk of amputation and death. It was concluded that angioplasty should be used first for patients with a life expectancy of ≤ 2 years, and that bypass is preferable when a vein graft is available. However, only a minority of the sample (42%) had DM, there was no subgroup analysis, and the study was not focused on patients with ulcers. Therefore, we cannot extrapolate these findings to patients with DFU and PAD.^117^

Revascularization should not be performed if there is no realistic chance of ulcer healing or when progression to amputation is inexorable (recommendation class III, level of evidence C). Patients with the following characteristics are not candidates for revascularization: significant frailty, low life expectancy, poor functional status, bedridden, large area of tissue destruction that makes the foot functionally unviable, and unable to undergo rehabilitation after revascularization. In these cases, primary amputation or a palliative approach must be decided upon by the patient and a multidisciplinary team.^13,14^

#### Angiosome-directed revascularization

**Figure 6:**
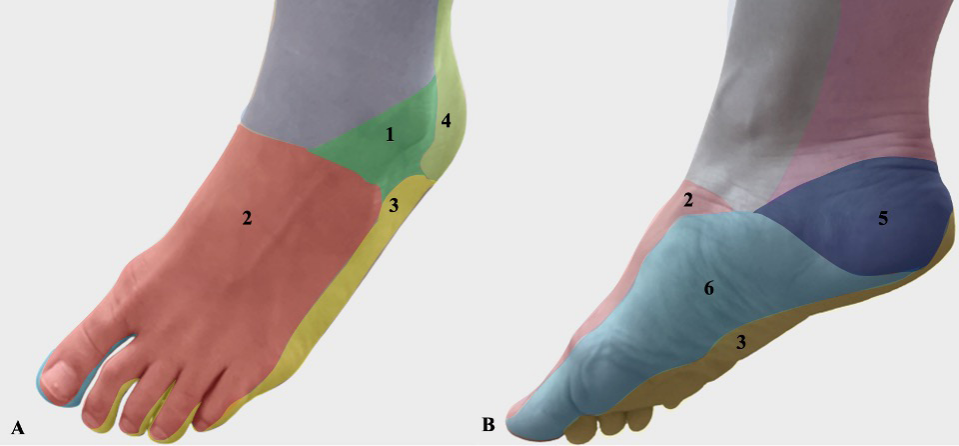
Illustration of foot angiosomes anterior view and posterior view.^119^ (1) Anterior communicating angiosome (of the peroneal artery); (2) Dorsalis pedis angiosome (of the anterior tibial artery); (3) Lateral plantar angiosome (of the posterior tibial artery); (4) Lateral calcaneal angiosome (of the peroneal artery); (5) Medial calcaneal angiosome (of the posterior tibial artery); (6) Medial plantar angiosome (of the posterior tibial artery).

In 1987, Taylor & Palmer proposed the concept of the angiosome, a three-dimensional unit of tissue nourished by an artery.^118^ The 3 main vessels (posterior tibial, fibular, and anterior tibial) nourish specific areas of the leg and foot (Figure 6). Hence, the aim is to identify and revascularize the artery that nourishes the specific area of tissue loss (direct revascularization), restoring pulsatile flow directly to the ischemic region, which makes healing more likely. Alternatively, non-angiosome targeted therapy (indirect revascularization) uses a “best vessel” approach, which selects the most suitable target artery, regardless of whether it is related to the area of tissue loss, thus restoring blood flow to the area through collateral vessels.^13,101,119^

Given that patients with DM have a poor network of collateral circulation and typically do not have a complete pedal arch or collateral flow from the peroneal artery to the foot, it seems intuitive that angiosome-directed revascularization would be more effective. Thus, the current consensus is that angiosome-directed revascularization should be performed whenever possible (recommendation class IIb, level of evidence C).^13,101^ However, due to the lack of standardized definitions and to methodological errors, the scientific robustness of the angiosome concept in patients with DM is unknown.^13^

Successful angioplasty of ≥ 1 occluded vessels is not the same as a clinically successful procedure, and before the procedure is completed, blood flow to the ulcerated area must be verified. If possible, opening multiple arteries may be useful, as long ≥ 1 feeds the ischemic area directly.^13^ The effectiveness of a revascularization procedure should be assessed using objective perfusion measurements, such as: blood pressure skin perfusion > 40 mmHg, TSP > 30 mmHg, or PtcO_2_ > 25 mmHg. Since skin oxygen tension increases progressively over a period of several weeks after successful percutaneous transluminal angioplasty, PtcO_2_ should be measured at least 1-3 weeks after the procedure.^13^

Extensive debridement or partial amputation of the foot should not be performed until the limb has been revascularized in patients with advanced ischemia, severe tissue loss, or no infection. In patients with severe infection, especially those with systemic inflammatory response syndrome, drainage must be performed before revascularization to control sepsis. As soon as sepsis is controlled and the patient is clinically stable, arterial studies and limb revascularization must be performed as soon as possible. After infection has been controlled and blood flow has been restored, definitive surgery can be performed to make the limb functional.^13^ Revascularization is another step in DFU treatment and, after the procedure, multidisciplinary follow-up must be guaranteed as part of a comprehensive care plan that addresses immediate infection treatment, ulcer debridement, biomechanical unloading, glycemic control, and comorbidity treatment.^13^

Intensive treatment is needed to reduce cardiovascular risk in these patients, including smoking cessation, hypertension treatment, blood glucose control, and therapy with statins and low-dose antiplatelet agents.^13,15^ Young et al.^120^ found that an aggressive approach to cardiovascular risk management reduced mortality in patients with neuroischemic DFU (5-year mortality decreased from 58% to 36%, with a 38% relative risk reduction). No specific evidence supports a single most appropriate antiplatelet agent or a combination of new direct oral anticoagulants in patients with PAD and DFU.^13^ Some studies have found that cardiovascular outcomes are lower in patients with PAD who use clopidogrel rather than acetylsalicylic acid.^121,122^ A meta-analysis of the COMPASS and VOYAGER trials found that low-dose rivaroxaban plus aspirin was superior to aspirin alone for reducing cardiovascular and limb outcomes, although it led to a relative increase in non-fatal major bleeding. This review concluded that the combination is beneficial for patients with PAD. However, the number of diabetic patients was limited (40-47%), few had an at-risk limb (2.8-31.8%), no information was provided about ulceration in the limb, and there was no subgroup analysis of patients with DFU and PAD.^123^Flowchart 4 summarizes the approach to patients with DFU and PAD, and Table 13 summarizes the main recommendations for diabetic patients with PAD.

**Flowchart 4:**
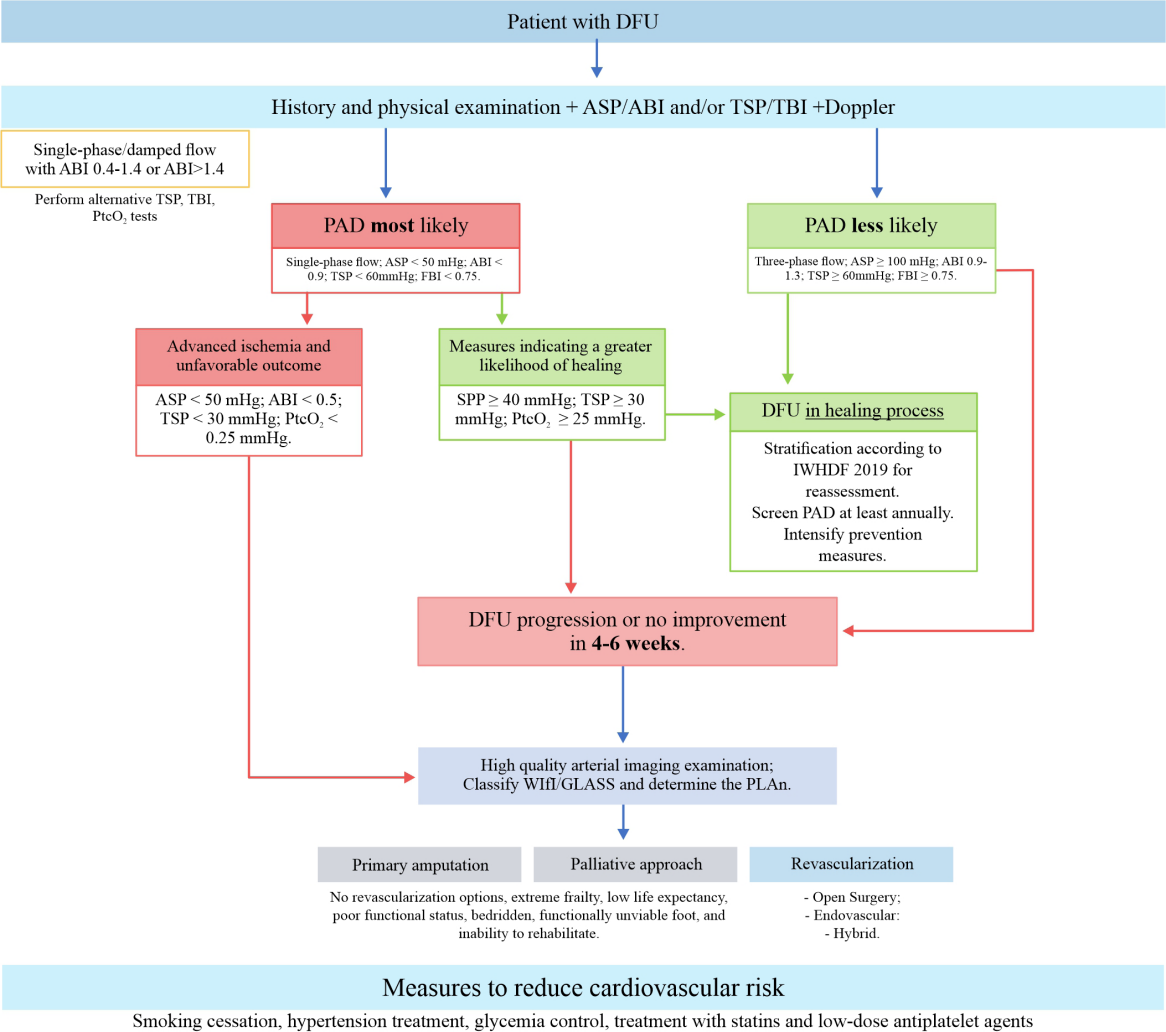
Summary of the approach to patients with DFU and PAD. ASP: ankle systolic pressure; ABI: ankle-brachial index; DFU: diabetic foot ulcer; GLASS: global limb anatomic staging system; IWGDF: International Working Group on the Diabetic Foot; PAD: peripheral arterial disease; PLAn: clinical decision-making method in 3 steps (P - patient risk, L - limb threat severity, and An - anatomic pattern of disease); PtcO_2_: transcutaneous oxygen pressure; TSP: toe systolic pressure; TBI: toe-brachial index; SPP: skin perfusion pressure; WIfI: Wound, Ischemia and Foot Infection classification system. Source: Conte et al.,^14^ Forsythe et al.,^97^ Schaper et al.^13^ and Chuter et al.^15^

**Table 13:** Recommendations for diabetic patients with PAD.

**Recommendations**	**Recommendation class and level of evidence**
1. Investigate PAD annually (anamnesis and pulse palpation) in all diabetic patients, even in those without foot ulceration.	Good clinical practice^124,125^
2. Complement the investigation of PAD and DFU with Doppler waveform evaluation in combination with ASP and ABI or TSP and TBI. ABI 0.9-1.3, TBI ≥ 0.75 and the presence of triphasic waves make a PAD diagnosis less likely.	Class I/level of evidence C^126-128^
3. In a patient with DFU and PAD, any of the following diagnostic test results (SPP ≥ 40 mmHg; TSP ≥ 30 mmHg; or PtcO_2_ ≥ 25 mmHg) increases the likelihood of healing by ≥ 25%.	Class I/level of evidence B^97,129^
4. Stratify amputation risk and the benefits of revascularization in patients with DFU and PAD using the WIfI classification system.	Class I/level of evidence B^13,81,117^
5. Always consider urgent vascular imaging and revascularization in DFU: 1) when ASP < 50 mmHg, ABI < 0.5, TSP < 30 mmHg, or PtcO_2_ < 25 mmHg; 2) when the ulcer is not healing or does not heal within 4-6 weeks despite optimal treatment.	Class IIa/level of evidence C^114,130,131^
6. Do not assume that diabetic microangiopathy is the cause of poor healing in patients with DFU.	Class III/level of evidence C^114,130,131^
7. Perform an arterial study of the entire limb with detailed images of the infrapopliteal arteries in surgical planning using: color duplex ultrasound, tomography angiography, magnetic resonance angiography, or digital angiography.	Class I/level of evidence C^14,132^
8. Treat patients with DFU and PAD urgently, since they are at high risk of limb loss.	Class I/level of evidence B^114,115^
9. Avoid revascularization when there is an unfavorable risk-benefit ratio.	Class III/level of evidence C^130,133^
10. Restore angiosome-guided direct blood flow when possible and objectively assess perfusion to determine its efficacy.	Class IIb/level of evidence C^134,135^
11. Choose the revascularization method based on individual factors, the anatomical complexity of PAD, autogenous vein availability, patient comorbidities, and service experience.	Class I/level of evidence B^14,96,115^
12. Ensure multidisciplinary follow-up after revascularization, including intensive treatment to reduce cardiovascular risk with PAD and DFU (smoking cessation, hypertension treatment, blood glucose control, administration of statins and antiplatelet agents.	Class I/level of evidence B^13,14,96^

ABI: ankle-brachial index; ASP: ankle systolic pressure; DFU: diabetic foot ulcer; PAD: peripheral arterial disease; PtcO_2_: transcutaneous oxygen pressure; SPP: skin perfusion pressure; TBI: toe-brachial index; TSP: systolic toe pressure; WIfI: wound, ischemia, and foot infection classification system. Source: Forsythe et al.;^97^ Schaper et al.;^13^ and Chuter et al.^15^

## CHAPTER 5. DIAGNOSING AND TREATING FOOT INFECTIONS IN PEOPLE WITH DIABETES

### Introduction

The complication that most often leads to hospitalization in diabetic patients is foot infection, and it is also a leading cause of amputation.^136,137^ Up to 17% of patients with an infected DFU progress to amputation within 1 year, while 10% become reinfected after wound healing.^137^ Considering only acute infections, the rates of minor amputation required for treatment can reach 40%.^138^ Therefore, to reduce morbidity and improve outcomes, a precise systematic approach is needed for early diagnosis of diabetic foot infections.^13^

### Diagnosis

Diabetic foot infections have been clinically defined by the IWGDF as “an inflammatory process in any tissue below the malleoli in a person with diabetes.” Despite this definition, however, it is possible for there to be no characteristic inflammatory process, especially in patients with associated PAD. Thus, assessment of factors that predispose patients to infection, such as deep, recurrent, long-standing or traumatic ulcers, chronic kidney disease, and diabetes-related immunity changes, can help resolve diagnostic suspicion.^139,140^ Assessing changes in temperature and edema can also be useful for diagnosis, since they are present in infectious processes and may be the result of underlying cellulitis or inflammatory processes related to Charcot arthropathy.^141^

Although most diabetic foot infections are superficial, deep infections have devastating potential, since they can spread upwards through the fascia and tendons of the deep compartments of the foot. In these cases, they can produce rapidly progressive infections, leading to increased internal compartment pressure, compartment syndrome, and necrosis due to tissue perfusion changes.^13^

When evaluating a foot ulcer in a diabetic patient, the presence of infection should be investigated. Clinical differentiation between a soft tissue infection, diabetic neuropathic osteoarthropathy, and osteomyelitis is a diagnostic challenge and requires a detailed work-up. Pain, fever, and elevated inflammatory markers can occur and overlap in all of these conditions.^142^ At this point, the ulcer must be classified according to the IWGDF/IDSA system, which has been validated for stratifying infections and has been included in the WIfI system, the most frequently used scale for diabetic foot classification (recommendation class I, level of evidence B).^13,136^ The classification method is detailed in Chapter 3.

Upon assessing the severity of the infectious process, hospital admission should be considered in severe and complex infections for which surgery is recommended, especially in patients with important comorbidities and PAD. Complementary laboratory assessment can determine severity parameters and help diagnose the infection when the physical examination is inconclusive. Leukocytosis, which is included in the IWGDF/ISDA classification system, is associated with the severity of the infectious process. Laboratory tests for infection markers are also indicated, such as erythrocyte sedimentation rate, C-reactive protein, and procalcitonin level (recommendation class IIb, level of evidence C). C-reactive protein and procalcitonin levels have greater sensitivity to earlier elevation, while an erythrocyte sedimentation rate > 70 mm/h is associated with bone infection.^13^

Additional parameters can be used to determine the presence of osteomyelitis in diabetic foot infections. There are 2 clinical predictors of osteomyelitis: the ulcer’s size/ depth and the probe-to-bone test. A deep ulcer with visual bone exposure has a 100% specificity, but only a 32% sensitivity, for diagnosing osteomyelitis. When the ulcerated area is > 2 cm, sensitivity increases to 52% and the specificity remains high (92%).^143^ The probe-to-bone test involves gently introducing a sterile blunt probe into the wound. If it reaches the bone or joint space, the result is positive. Positive results indicate bone infection with a sensitivity of 87% and specificity of 83%.^144^ The gold standard for diagnosing osteomyelitis is aseptic bone biopsy (percutaneous or surgical route). A positive culture or histology is the only way to determine the specific pathogen and guide antibiotic therapy. Although feasible, bone biopsy should be considered in cases where there may be resistance, where there has been previous treatment, or where current antimicrobial treatment has failed.^142^

Soft tissue cultures must be collected aseptically from all wounds (curettage or biopsy) to guide treatment. When the culture is obtained from deep tissue and a single pathogen grows, it may suggest the etiology of the associated bone infection, although studies have found a correlation between soft tissue and bone cultures in < 50% of cases, being as low as 17.4% in some cases.^145,146^ Thus, in most cases material should be collected aseptically, surgically, or percutaneously due to the reliable results.^147^ Acute infections of lesser severity that have not undergone previous treatment can be considered for empirical treatment without culture collection, although culture collection should be performed or repeated when the clinical course is unfavorable or when the ulcer is subject to surgical debridement (recommendation class IIa, level of evidence C).^11,13^

### Imaging exams

Due to its easy access and low cost, radiography should be the initial imaging modality for patients with a DFU and suspected osteomyelitis (recommendation class I, level of evidence B).^142^ A meta-analysis found that radiography had a sensitivity of 28% and a specificity of 68%.^143^ However, when combined with the probe-to-bone test, the sensitivity and specificity increase to 97% and 93%, respectively.^148^ Therefore, these tests should be combined for initial diagnosis.

Radiographic changes are only apparent when bone loss of 30%-50% has occurred, and they may not be visualized in the first 10 days of infection.^142^Chart 6 shows plain radiography parameters that are associated with soft tissue changes and osteomyelitis.^149^

**Chart 6:** Radiographic parameters associated with soft tissue changes and osteomyelitis.

Ø **Soft tissue changes:**
• abnormal density of fatty tissue;
• presence of gas;
• loss of tissue planes.
Ø **Bone changes:**
• blurring or loss of cortical bone;
• ill-defined erosions and bone sequestration;
• periosteal reactions;
bone sclerosis.

Source: Abikhzer et al.^142^ and Aragón-Sánchez et al.^149^

CT has high spatial resolution and provides better assessment of bone structures than plain radiography when assessing osteomyelitis. CT can also detect gas and small or deep abscesses that are imperceptible in radiography. It has a sensitivity of 67% and a specificity of 50% for diagnosing osteomyelitis.^142^ Nevertheless, MRI is the main diagnostic modality for osteomyelitis in diabetic patients (recommendation class I, level of evidence B). MRI can also show bone marrow signal changes, which may manifest before bone lysis becomes evident in radiography or CT.^142^ It has a sensitivity of 93% and a specificity of 75% for diagnosing diabetic foot osteomyelitis.^150^ Routine non-contrast MRI with fat-suppressed sequences (T1, T2, and STIR) on multiple orthogonal planes can be used to diagnose osteomyelitis. Contrast is often necessary and may not be feasible in diabetic patients with chronic kidney disease.^142^

When bone marrow is replaced with a low T1 signal (darker than skeletal muscle), it is typically associated with osteomyelitis. This may also be associated with the loss of a normal cortical T1 signal due to bone lysis or the presence of periosteal edema, which increases diagnostic confidence for osteomyelitis. The “ghost” effect of bone structures involved in osteomyelitis is also a useful sign. Bones are imperceptible in T1-weighted sequences due to marrow replacement and cortex loss, becoming readily visible in fluid-sensitive (fat-saturated T2 sequence) or contrast-enhanced sequences. A diagnosis of osteomyelitis is reinforced when bone marrow adjacent to an ulcer (with or without a fistula) is replaced with soft tissue edema.^142^

Although positron emission tomography with fluorodeoxyglucose and scintigraphy with labeled leukocytes can also help clarify the diagnosis, due to their higher cost and more limited availability, they should be reserved for when a conclusion cannot be reached from the initial assessment.^142^

### Treatment

Flowchart 5 summarizes diabetic foot infection treatment. Empirical antibiotic therapy must be based on the pathogen’s local susceptibility data, considering availability and possible drug interactions. Virulent pathogens such as *Staphylococcus aureus* and beta-hemolytic streptococci should be treated, considering that less virulent pathogens only tend appear as local contaminants/colonizers. Additionally, for all tropical countries, the IWGDF recommends including an antibiotic that is effective against *Pseudomonas aeruginosa* due to its high prevalence, especially when the lesion has been in contact with humid media.^13^

**Flowchart 5:**
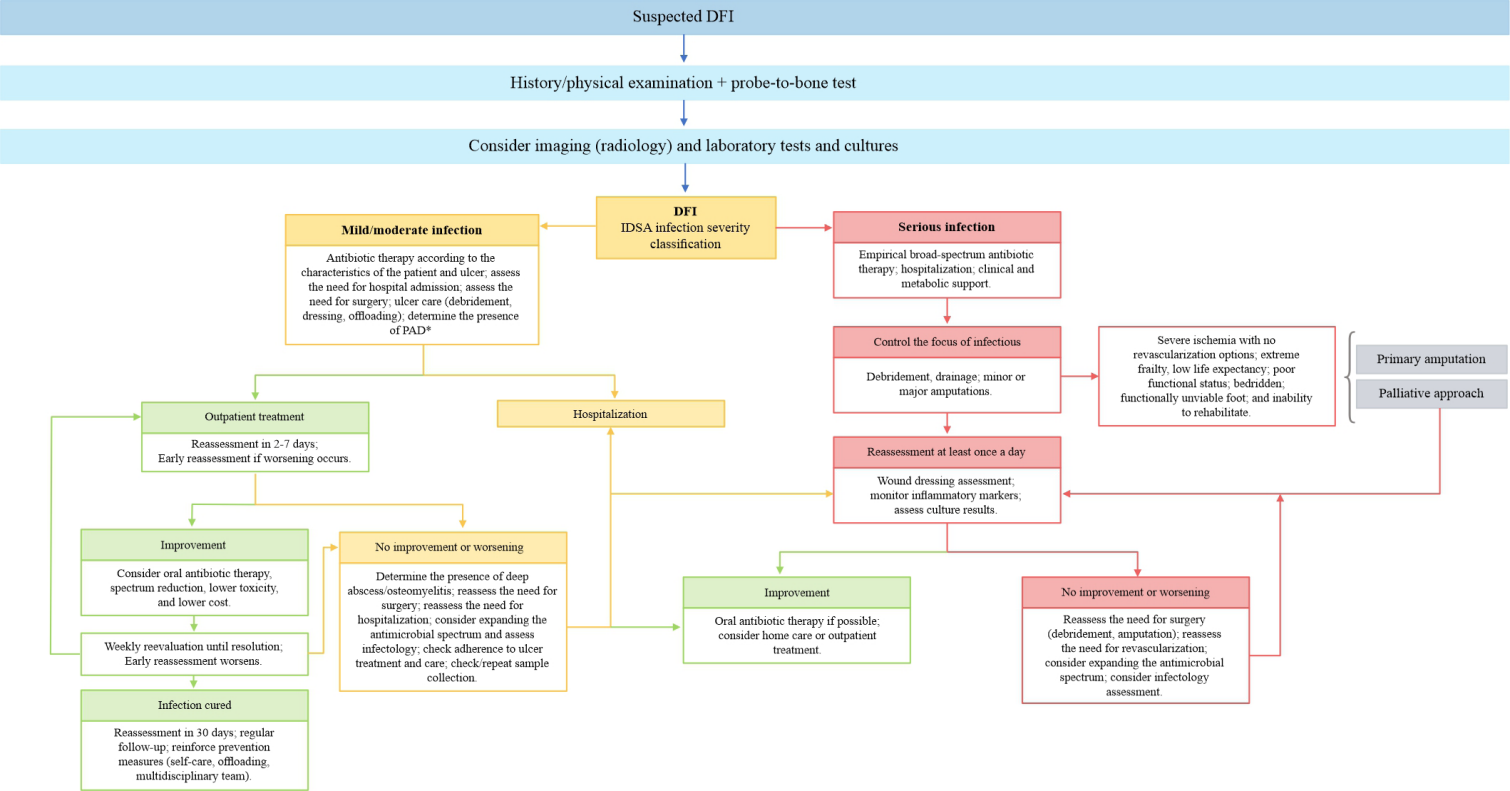
Treatment of infected diabetic foot ulcers.*Chapter 4 describes diabetic foot ulcer treatment in patients with peripheral arterial disease. DFI = diabetic foot infection; PAD = peripheral arterial disease. Source: Conte et al.^14^ and Schaper et al.^13^

More complex cases should involve consultation with an infectious disease specialist, including individualized discussion of each case.^13^Table 14 suggests combinations of empirical antibiotic therapy according to infection severity. According to expert opinion, simple wounds can receive antibiotic therapy for 1 to 2 weeks. The treatment period may be extended to 4 weeks, especially in extensive wounds that are healing and in cases with more severe PAD, comorbidities associated with delayed wound healing, or an increased rate of infection treatment failure (recommendation class IIa, level of evidence C).^151^ If the infection has not resolved after this period, the possibility of treatment failure must be considered and the treatment should be reassessed.^13,151^

**Table 14:** Suggested empirical antibiotic regimens for diabetic foot infection treatment.

**Infection severity**	**Additional factors**	**Pathogens involved**	**Suggested empirical therapy**
**Light**	No factors	Gram-positive	SPRP, 1st generation cephalosporin.
	Recent antibiotic use	Gram-positive and gram-negative bacilli	In case of allergy: clindamycin, fluoroquinolones, macrolide, doxycycline.
	Risk of MRSA	MRSA	Beta-lactams with beta-lactamase inhibitor; S/T; fluoroquinolones.
**Moderate or severe**	No factors	Gram-positive and negative bacilli	Beta-lactams with beta-lactamase inhibitor.
	Recent antibiotic use	Gram-positive and negative bacilli	Beta-lactams with beta-lactamase inhibitor; Carbapenems;
	Macerated ulcer or hot weather	Gram-positive, gram-negative bacilli, Pseudomonas	(request evaluation from an infectious disease specialist)
	Ischemic limb, necrosis/gas	Gram-positive, gram-negative bacilli, Anaerobes	Beta-lactams with beta-lactamase inhibitor; SPRP + ceftazidime; SPRP + ciprofloxacin; Carbapenems.
	Risk Factors for MRSA	MRSA	Beta-lactams with beta-lactamase inhibitor; Carbapenems; second- and third-generation cephalosporins + clindamycin or metronidazole.
	Risk factors for gram bacilli resistance	BLPO	Consider adding or replacing with vancomycin, linezolid, or daptomycin.

BLPO: beta-lactamase-producing organism; MRSA: methicillin-resistant *Staphylococcus aureus*; SPRP: semisynthetic penicillinase-resistant penicillin; S/T: sulfamethoxazole + trimethoprim. Source: Schaper et al.^13^

Using topical antibiotics in the wound or indiscriminate use of systemic/topical antibiotics to prevent infection in non-infected ulcers is not recommended. When wound infection has not been confirmed after detailed evaluation, antibiotics provide no actual benefits and could induce bacterial resistance, which overrides any theoretical benefit they might provide in such scenarios (recommendation class III, level of evidence B).^13^

Although cases of mild infection occur, most diabetic foot infections will require surgical intervention to resolve. In cases of severe infection with necrosis and deep abscesses, surgical evaluation is essential and drainage/decompression should ideally be performed urgently, in most cases within 24 hours.^11^ In general, urgent cases are associated with soft tissue infection, although this is rare in isolated bone infections. Initially, the surgical procedure must involve resection of devitalized, infected tissue and pressure reduction in deep compartments, maintaining all viable skin coverage, even in non-critical areas, considering its use in future surgeries after the infection has been controlled.^152^

Although the gold standard treatment for osteomyelitis involves resection of the compromised bone segment, similar success rates have been described for osteomyelitis treatment without bone resection or conservative bone resection, especially when limited to the forefoot, with remission rates in some series reaching 64% in 1 year.^153,154^ The suggested treatment time in such cases is 6 weeks, with improvement observed ideally in the first 2-4 weeks (recommendation class IIa, level of evidence B). If no resolution occurs during this period, the approach should be changed, including a biopsy to identify the pathogen or resection of the involved structure.^155^

When complete resection of the bone lesion is performed, antibiotic therapy can be reduced to a few days, especially when cultures of bone tissue fragments from the resection edge are negative. When the resection edge yields positive cultures, 6 weeks of treatment is recommended (recommendation class IIb, level of evidence C).^13^

Similar to serious infections, osteomyelitis treatment can be modified from intravenous to oral, while maintaining an antibiotic regimen with similar coverage and dosage in the upper range. Prolonging treatment beyond 6 weeks has shown no benefits^88^ and, according to the IWGDF, long-term suppressive antibiotic therapy should only be used in cases involving a large amount of necrotic bone tissue that is not amenable to removal or in cases of infected orthopedic material.^155^ Follow-up with laboratory monitoring is suggested, including foot X-rays and serial measurement of C-reactive protein and erythrocyte sedimentation rate. The patient can be considered cured after 1 year of follow-up.^138^

Regarding adjuvant therapies for infection, there are no high quality recommendations about hyperbaric or topical oxygen therapy, granulocyte colony-stimulating factor, topical antiseptics,^89^ silver compounds^90^ or negative pressure therapy for diabetic foot infection (recommendation class III, level of evidence B).^13,156,157^ To date, trials regarding these adjuvant therapies have been low quality and do not substantially support their use in light of the cost and potential adverse effects.^13^Table 15 summarizes the main recommendations for treating diabetic foot infections.

**Table 15:** Summary of the main recommendations for diabetic foot infection treatment.

**Recommendations**	**Recommendation class and level of evidence**
1. Determine the presence of infection based on signs and symptoms of local inflammation in every diabetic patient with foot ulcers.	Class I/level of evidence C^158-162^
2. Use the Infectious Diseases Society of America classification system to stratify severity.	Class I/level of evidence B^90,163,164^
3. Use CRP, ESR, or procalcitonin measurement in cases of diagnostic uncertainty.	Class IIb/level of evidence C^165-170^
4. Use plain radiography, the probe-to-bone test, and ESR measurement to diagnose osteomyelitis.	Class I/level of evidence B^171-175^
5. Request MRI (preferably) or PET-CT/scintigraphy with marked leukocytes to diagnose osteomyelitis only if there is diagnostic uncertainty after initial evaluation.	Class I/level of evidence B^176-180^
6. Collect cultures aseptically to determine the pathogen involved in all infected diabetic foot ulcers.	Class I/level of evidence C^145,181-183^
7. Collect bone cultures (surgically or percutaneously) to identify the pathogen in cases of osteomyelitis, especially when empirical treatment has failed or there is a high probability of osteomyelitis and diagnostic uncertainty after imaging tests.	Class IIa/level of evidence C^145,181-183^
8. Use antibiotic therapy to treat infected diabetic foot ulcers according to the sensitivity profile of the likely pathogens involved, infection severity, and previous antibiotic use.	Class I/level of evidence B^184-190^
9. Start parenteral antibiotic therapy in cases of severe infection; the treatment can be changed to an oral regimen after clinical improvement and when feasible from the point of view of tolerance and bioavailability.	Class IIa/level of evidence C^188-190^
10. Do not use topical antibiotics to treat wound infections.	Class III/level of evidence B^191,192^
11. Administer antibiotics for 1-2 weeks for soft tissue infection and 3-4 weeks for improving extensive lesions and/or concurrent severe PAD, which may prolong the healing period.	Class IIa/level of evidence C^189,190,193-195^
12. Administer antibiotics for ≤ 6 weeks in cases of osteomyelitis, evaluating the results in the first 2 to 4 weeks, considering new collection or treatment adjustment according to the culture results.	Class IIa/level of evidence B^196-198^
13. Administer a spectrum of antibiotics for the most prevalent gram-positive and gram-negative lesions in cases of PAD, previous antibiotic use, or moderate/severe lesions. Add strict anaerobe coverage for moderate/severe cases and consider adding coverage for *Pseudomonas aeruginosa*.	Class IIa/level of evidence C^189-195,199^
14. Reevaluate and adjust antibiotic therapy according to the culture sensitivity results; do not use antimicrobials in non-infected wounds to avoid infection or accelerate healing.	Class I/level of evidence C^200,201^
15. Consider reevaluating treatment and collecting new cultures if treatment fails after the expected time.	Class IIb/level of evidence C^13^
16. Osteomyelitis treatment may not involve surgical resection of the bone when limited to the forefoot. In other cases, consider surgical resection, especially when there is associated soft tissue infection.	Class IIb/level of evidence B^201-207^
17. During surgical bone resection, we suggest collecting a fragment of the remaining bone portion for culture and residual infection assessment.	Class IIb/level of evidence C^208-211^
18. Antibiotic treatment for osteomyelitis can be shortened if the entire focus is removed from the bone and the residual fragment culture is negative. If the culture is positive, continue treatment for 6 weeks.	Class IIb/level of evidence C^208-211^
19. Do not use hyperbaric oxygen therapy, topical oxygen therapy, routine topical antiseptics, silver preparations, or negative pressure therapy to treat ulcers if the only recommendation is to treat infection.	Class III/level of evidence B^212-215^

CRP: C-reactive protein; ESR: erythrocyte sedimentation rate; MRI: magnetic resonance imaging; PAD: peripheral arterial disease; PET-CT: Positron emission tomography–computed tomography. Adapted from Schaper et al.^13^

## CHAPTER 6. CHARCOT ARTHROPATHY

### Introduction

Although Charcot arthropathy was first described in1868, its pathophysiology remains undefined. It is associated with conditions that cause neuropathy of the lower limbs, and diabetes is its main cause.^216^ Its incidence among people with DM can vary from 0.1% to 13%, reaching up to 29% in patients who already have neuropathy.^217,218^ Since deformities resulting from Charcot arthropathy lead to inadequate pressure distribution in the foot, they are an important cause of foot ulcers in diabetic patients.^217^

### Diagnosis

Charcot arthropathy is defined as a non-infectious neuro-osteoarthropathy of the bones and joints that leads to changes in sensitivity and destruction of foot architecture.^219^ It usually involves the midfoot, hindfoot, and ankle, and 2 mechanisms for its development have been described.^216,217^ According to neurovascular theory, its development can be explained through dysautonomia caused by neuropathy: increased vascularization and the stimulation of osteoclastic activity is the cause of the deformities. According to neurotraumatic theory, multiple joint and bone injuries develop due to a lack of protective sensitivity and inadequate injury healing, resulting in the development of arthropathy.^216,220^

Although early diagnosis is decisive for preserving the limb, in up to 79% of cases error leads to delayed diagnosis by as much as 7 months.^217^ Both acute and chronic presentations are possible, and diagnosis is still essentially clinical. The acute form presents with erythema, edema, pain, and increased foot temperature and is often confused with other diseases, such as cellulitis, gout, sprains, or deep vein thrombosis. The chronic form is the most characteristic, including plantar arch loss and a ‘rocker bottom’ deformity.^221^ Acute Charcot arthropathy should be considered for presentations involving edema, pain, and erythema of the foot without evident skin lesions (good clinical practice).

In suspected Charcot arthropathy patients with intact skin, infrared skin temperature can be measured at the highest sites on the foot or ankle and compared with the contralateral limb at the same anatomical point (recommendation class IIb, level of evidence C). A 2 °C increase in skin temperature compared to the contralateral foot has been used as a threshold for diagnosing active Charcot arthropathy.^222^ In the absence of other signs or symptoms of inflammation (ie, redness or swelling), an isolated increase in foot temperature may not always be indicative of active Charcot neuropathic osteoarthropathy and should be interpreted in the context of other clinical findings.^223,224^ Although an essential part of the diagnostic evaluation, an isolated elevation in foot skin temperature is insufficient to either diagnose or rule out active disease. Consequently, asymmetric temperature elevation is sensitive but not specific in active Charcot arthropathy diagnosis.

Ideally, plain bilateral radiography should be performed for comparison in patients with diabetes and suspected active Charcot arthropathy (recommendation class IIa, level of evidence B). Radiography should include anteroposterior, medial oblique, and lateral views in a diabetic patient with suspected active Charcot neuropathic osteoarthropathy. Views of the ankle and foot should include anteroposterior and lateral projections. Ideally, standing (ie, “weight bearing”) X-rays should be taken. If a patient is unable to stand up, non-weight bearing X-rays are an alternative but may not show misalignments, which are more apparent in the standing position.^225^

Since non-weight-bearing X-rays may not show changes, weigh-bearing X-rays should always be requested. If the X-ray shows no changes, the diagnosis should not be discarded, since such changes are not expected at the beginning of the process.^221^ MRI can be used for diagnosis, but changes may be indistinguishable from osteomyelitis and must be correlated with clinical and laboratory tests, biopsies, and bone cultures.^221^ If MRI is unavailable or contraindicated, scintigraphy, CT, or single-photon emission CT may be considered to complement diagnosis of active Charcot neuropathic osteoarthropathy.^226,227^

Biochemical markers are generally unaltered in Charcot arthropathy, although C-reactive protein and erythrocyte sedimentation rate levels can be used for differential diagnosis of a lesion due to infection.^216,217,219^ Eichenholtz divided acute Charcot arthropathy into 4 stages, as described in Table 16.^218^

**Table 16:** Eichenholtz classification modified.

**Stage**	**Radiography**	**Clinical**	**Suggested treatment**
**0 (pre-Charcot – prodromal)**	Normal.	Edema, erythema, heat.	Patient education, serial radiographs, discharge with limb protection.
**1 (development)**	Osteopenia, fragmentation, subluxations, or joint dislocations.	Edema, erythema, heat, ligament changes.	Discharge with limb protection and a full cast or removable orthosis until radiographic resolution of the fragments and temperature reduction.
**2 (coalescence)**	Absorption of debris, sclerosis, fusion of large fragments.	Reduction of edema, erythema and heat.	Use full cast or other protective orthoses for discharge.
**3 (reconstruction)**	Consolidation of the deformity, fibrous ankylosis, bone fragments with smooth, rounded edges.	Absence of edema, erythema, warmth, joint stability, and fixed deformity.	Shoes adapted for ulcer prevention, consider referral to an orthopedic surgeon to assess deformity correction.

Source: Adapted from Rosenbaum et al.^228^

### Treatment

Patients with Charcot arthropathy should be referred to a multidisciplinary team for monitoring and care. Initial treatment is based on load relief through full cast immobilization, which has been successfully used to treat the acute phase.^229^ Immobilization to knee height should be begin immediately when active Charcot arthropathy is suspected in a diabetic patient with intact skin (recommendation class IIa, level of evidence C). Early detection, immobilization, and load reduction for the diseased foot have been shown to minimize development of the deformity.^230,231^ Immobilization should continue until symptoms remit, and patients should be followed up with serial radiographs and clinical examination of the limb. A difference of < 2 °C in skin temperature between the limbs and the consolidation of bone changes in radiography are associated with resolution of the process.^217,229^

Contraindications to full cast immobilization should be considered in patients with actively infected ulcers. Patients undergoing treatment must be monitored on a weekly basis. After remission of the initial phase, orthopedic shoes are recommended to prevent recurrence, ulcerations and deformities (recommendation class IIa, level of evidence C).^217^

In addition to orthopedic shoes, chronic phase Charcot arthropathy treatment may involve surgery. These patients should be referred to a multidisciplinary team with an orthopedic surgeon to evaluate possible surgical recommendations for preventing ulcerations or disease recurrence.^232,233^Flowchart 6 outlines an approach to diabetic patients with suspected Charcot arthropathy, while Table 17 summarizes the main recommendations.

**Flowchart 6:**
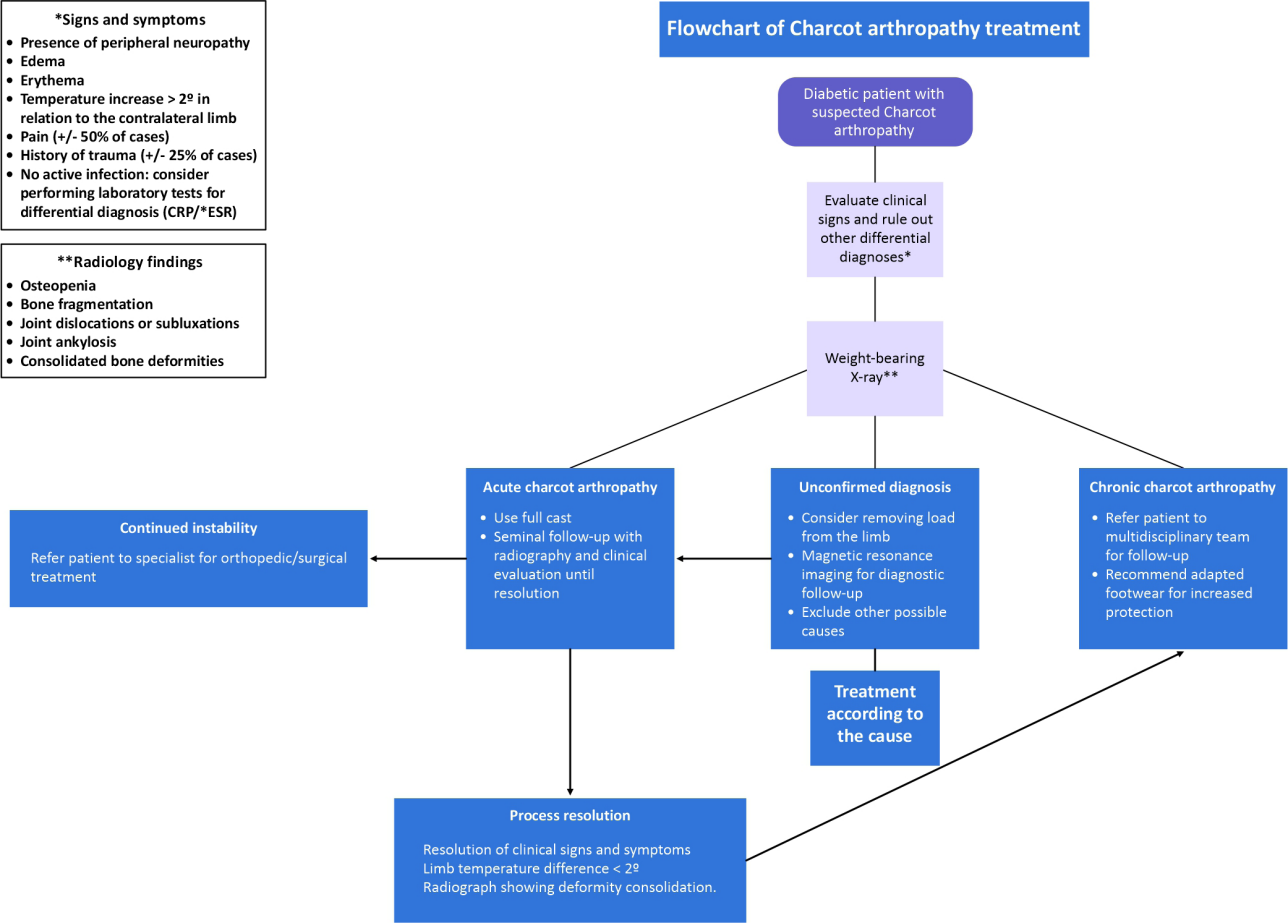
Proposed flowchart for Charcot arthropathy. CCRP: C-reactive protein; ESR: erythrocyte sedimentation rate. Source: Adapted from Milne et al.^233^

**Table 17:** Recommendations for Charcot arthropathy.

**Recommendations**	**Recommendation class and level of evidence**
1. In diabetic patients, pay attention to foot fractures since they may develop into Charcot arthropathy.	Good clinical practice^216,217,220^
2. Always consider the possibility of acute Charcot arthropathy as a differential diagnosis in diabetic patients with peripheral neuropathy who present with edema, erythema, and increased foot temperature (in relation to the contralateral foot) even in the absence of pain.	Good clinical practice^217,221^
3. In patients with suspected Charcot arthropathy and intact skin, consider measuring infrared skin temperature at the highest temperature sites on the foot or ankle compared to the same points on the contralateral limb.	Class IIb/level of evidence C^222-224^
4. To diagnose Charcot arthropathy, we suggest performing a weight-bearing X-ray and, if suspicion remains after normal X-ray results, consider nuclear magnetic resonance imaging.	Class IIa/level of evidence B^225,234-236^
5. We suggest avoiding C-reactive protein, erythrocyte sedimentation rate, complete blood count, alkaline phosphatase, or other blood tests in a diabetic patient with suspected active Charcot neuropathic osteoarthropathy and intact skin to diagnose or exclude the disease.	Class III/level of evidence C^237-242^
6. Initiate leg immobilization/weight bearing if Charcot arthropathy is clinically suspected while performing confirmation studies.	Class I/level of evidence C^229-231^
7. We recommend treatment with weight-bearing devices, preferably non-removable ones; if this is not possible, use removable devices.	Class IIa/level of evidence C^230,231^
8. In patients with acute Charcot arthropathy, limb temperature should be clinically monitored each week until it is reduced to a difference of < 2º; serial radiographs should be performed until there are no new changes and bone lesions consolidate.	Class IIa/level of evidence C^243,244^

Adapted from Milne et al.^233^

## References

[1] Nobre MRC, Bernardo WM (2002). Diretrizes AMB/CFM. Rev Assoc Med Bras.

[2] Migowski A, Stein AT, Santos MS, Fernandes MM, Ferreira DM, Ferreira CB (2016). Diretrizes metodológicas: elaboração de diretrizes clínicas..

[3] Zheng Y, Ley S, Hu F (2018). Global aetiology and epidemiology of type 2 diabetes mellitus and its complications. Nat Rev Endocrinol.

[4] Armstrong DG, Boulton AJM, Bus SA (2017). Diabetic foot ulcers and their recurrence. N Engl J Med.

[5] Stern JR, Wong CK, Yerovinkina M (2017). A meta-analysis of long-term mortality and associated risk factors following lower extremity amputation. Ann Vasc Surg.

[6] Walsh JW, Hoffstad OJ, Sullivan MO, Margolis DJ (2016). Association of diabetic foot ulcer and death in a population-based cohort from the United Kingdom. Diabet Med.

[7] Prompers L, Schaper N, Apelqvist J (2008). Prediction of outcome in individuals with diabetic foot ulcers: focus on the differences between individuals with and without peripheral arterial disease. The EURODIALE study. Diabetologia.

[8] Mesquita LO, Aquino EC, Gouvea ECD, Oliveira PPV, França GVA (2022). Mortalidade por diabetes mellitus no Brasil, 2010 a 2021. Boletim Epidemiológico.

[9] Santos AAA, Gomes AFL, Silva FSS (2022). Tendência temporal das complicações do pé diabético e da cobertura da Atenção Primária à Saúde nas capitais brasileiras, 2008-2018. Rev Bras Med Fam Comunidade.

[10] Parisi MC, Moura A, Menezes FH (2016). Baseline characteristics and risk factors for ulcer, amputation and severe neuropathy in diabetic foot at risk: the BRAZUPA study. Diabetol Metab Syndr.

[11] Hingorani A, LaMuraglia GM, Henke P (2016). The management of diabetic foot: a clinical practice guideline by the Society for Vascular Surgery in collaboration with the American Podiatric Medical Association and the Society for Vascular Medicine. J Vasc Surg.

[12] Botros M, Kuhnke J, Embil J, Canadian Association of Wound Care (2017). Foundations of best practice for skin and wound management: a supplement of Wound Care Canada..

[13] Schaper NC, van Netten JJ, Apelqvist J, Bus SA, Hinchliffe RJ, Lipsky BA (2020). Practical Guidelines on the prevention and management of diabetic foot disease (IWGDF 2019 update). Diabetes Metab Res Rev.

[14] Conte MS, Bradbury AW, Kolh P (2019). Global vascular guidelines on the management of chronic limb-threatening ischemia. Eur J Vasc Endovasc Surg.

[15] Chuter V, Quigley F, Tosenovsky P (2022). Australian guideline on diagnosis and management of peripheral artery disease: part of the 2021 Australian evidence-based guidelines for diabetes-related foot disease. J Foot Ankle Res.

[16] European Society of Cardiology (2023). Recommendations for guidelines production.

[17] Fosse S, Hartemann-Heurtier A, Jacqueminet S, Ha Van G, Grimaldi A, Fagot-Campagna A (2009). Incidence and characteristics of lower limb amputations in people with diabetes. Diabet Med.

[18] Ikonen TS, Sund R, Venermo M, Winell K (2010). Fewer major amputations among individuals with diabetes in Finland in 1997-2007: a population-based study. Diabetes Care.

[19] Armstrong DG, Boulton AJM, Bus SA (2017). Diabetic foot ulcers and their recurrence. N Engl J Med.

[20] Bus SA, Armstrong DG, Gooday C (2020). Guidelines on offloading foot ulcers in persons with diabetes (IWGDF 2019 update). Diabetes Metab Res Rev..

[21] Mens MA, Busch-Westbroek TE, Bus SA (2023). The efficacy of flexor tenotomy to prevent recurrent diabetic foot ulcers (DIAFLEX trial): study protocol for a randomized controlled trial. Contemp Clin Trials Commun.

[22] Bus SA, Lavery LA, Monteiro-Soares M (2020). Guidelines on the prevention of foot ulcers in persons with diabetes (IWGDF 2019 update). Diabetes Metab Res Rev.

[23] Crawford F, Cezard G, Chappell FM (2015). A systematic review and individual patient data meta-analysis of prognostic factors for foot ulceration in people with diabetes: the international research collaboration for the prediction of diabetic foot ulcerations (PODUS). Health Technol Assess.

[24] Mishra SC, Chhatbar KC, Kashikar A, Mehndiratta A (2017). Diabetic foot. BMJ.

[25] Feldman EL, Callaghan BC, Pop-Busui R (2019). Diabetic Neuropathy. Nat Rev Dis Primers.

[26] Adiewere P, Gillis RB, Imran Jiwani S, Meal A, Shaw I, Adams GG (2018). A systematic review and meta-analysis of patient education in preventing and reducing the incidence or recurrence of adult diabetes foot ulcers (DFU). Heliyon.

[27] Skafjeld A, Iversen MM, Holme I, Ribu L, Hvaal K, Kilhovd BK (2015). A pilot study testing the feasibility of skin temperature monitoring to reduce recurrent foot ulcers in patients with diabetes: a randomized controlled trial. BMC Endocr Disord.

[28] Wijlens AM, Holloway S, Bus SA, van Netten JJ (2017). An explorative study on the validity of various definitions of a 2·2 °C temperature threshold as warning signal for impending diabetic foot ulceration. Int Wound J.

[29] van Netten JJ, Prijs M, van Baal JG, Liu C, van der Heijden F, Bus SA (2014). Diagnostic values for skin temperature assessment to detect diabetes-related foot complications. Diabetes Technol Ther.

[30] Waaijman R, de Haart M, Arts ML (2014). Risk factors for plantar foot ulcer recurrence in neuropathic diabetic patients. Diabetes Care.

[31] Young MJ, Cavanagh PR, Thomas G, Johnson MM, Murray H, Boulton AJ (1992). The effect of callus removal on dynamic plantar foot pressures in diabetic patients. Diabet Med.

[32] Pitei DL, Foster A, Edmonds M (1999). The effect of regular callus removal on foot pressures. J Foot Ankle Surg.

[33] Rasmussen A, Bjerre-Christensen U, Almdal TP, Holstein P (2013). Percutaneous flexor tenotomy for preventing and treating toe ulcers in people with diabetes mellitus. J Tissue Viability.

[34] van Netten JJ, Bril A, van Baal JG (2013). The effect of flexor tenotomy on healing and prevention of neuropathic diabetic foot ulcers on the distal end of the toe. J Foot Ankle Res.

[35] Rizzo L, Tedeschi A, Fallani E (2012). Custom-made orthesis and shoes in a structured follow-up program reduces the incidence of neuropathic ulcers in high-risk diabetic foot patients. Int J Low Extrem Wounds.

[36] Lavery LA, LaFontaine J, Higgins KR, Lanctot DR, Constantinides G (2012). Shear-reducing insoles to prevent foot ulceration in high-risk diabetic patients. Adv Skin Wound Care.

[37] Scirè V, Leporati E, Teobaldi I, Nobili LA, Rizzo L, Piaggesi A (2009). Effectiveness and safety of using Podikon digital silicone padding in the primary prevention of neuropathic lesions in the forefoot of diabetic patients. J Am Podiatr Med Assoc.

[38] Bus SA, Waaijman R, Arts M (2013). Effect of custom-made footwear on foot ulcer recurrence in diabetes: a multicenter randomized controlled trial. Diabetes Care.

[39] Ulbrecht JS, Hurley T, Mauger DT, Cavanagh PR (2014). Prevention of recurrent foot ulcers with plantar pressure- based in- shoe orthoses: the CareFUL prevention multicenter randomized controlled trial. Diabetes Care.

[40] Mueller MJ, Sinacore DR, Hastings MK, Strube MJ, Johnson JE (2003). Effect of Achilles tendon lengthening on neuropathic plantar ulcers: a randomized clinical trial. J Bone Joint Surg Am.

[41] Armstrong DG, Rosales MA, Gashi A (2005). Efficacy of fifth metatarsal head resection for treatment of chronic diabetic foot ulceration. J Am Podiatr Med Assoc.

[42] Faglia E, Clerici G, Caminiti M, Curci V, Somalvico F (2012). Feasibility and effectiveness of internal pedal amputation of phalanx or metatarsal head in diabetic patients with forefoot osteomyelitis. J Foot Ankle Surg.

[43] Melai T, Schaper NC, Ijzerman TH (2013). Lower leg muscle strengthening does not redistribute plantar load in diabetic polyneuropathy: a randomised controlled trial. J Foot Ankle Res.

[44] De León Rodriguez D, Allet L, Golay A (2013). Biofeedback can reduce foot pressure to a safe level and without causing new at-risk zones in patients with diabetes and peripheral neuropathy. Diabetes Metab Res Rev.

[45] Iunes DH, Rocha CB, Borges NC, Marcon CO, Pereira VM, Carvalho LC (2014). Self-care associated with home exercises in patients with type 2 diabetes mellitus. PLoS One.

[46] Fayed EE, Badr NM, Mahmoud S, Hakim SA (2016). Exercise therapy improves plantar pressure distribution in patients with diabetic peripheral neuropathy. Int J Pharm Tech Res.

[47] Mueller MJ, Tuttle LJ, Lemaster JW (2013). Weight-bearing versus nonweight-bearing exercise for persons with diabetes and peripheral neuropathy: a randomized controlled trial. Arch Phys Med Rehabil.

[48] Kooiman TJM, de Groot M, Hoogenberg K, Krijnen WP, van der Schans CP, Kooy A (2018). Self-tracking of physical activity in people with type 2 diabetes: a randomized controlled trial. Comput Inform Nurs.

[49] Bus SA, van Deursen RW, Armstrong DG, Lewis JE, Caravaggi CF, Cavanagh PR (2016). Footwear and offloading interventions to prevent and heal foot ulcers and reduce plantar pressure in patients with diabetes: a systematic review. Diabetes Metab Res Rev.

[50] Elraiyah T, Prutsky G, Domecq JP (2016). A systematic review and metaanalysis of off-loading methods for diabetic foot ulcers. J Vasc Surg.

[51] Oliveira AL, Moore Z (2015). Treatment of the diabetic foot by offloading: a systematic review. J Wound Care.

[52] Blume PA, Paragas LK, Sumpio BE, Attinger CE (2002). Single-stage surgical treatment of noninfected diabetic foot ulcers. Plast Reconstr Surg.

[53] Sayner LR, Rosenblum BI, Giurini JM (2003). Elective surgery of the diabetic foot. Clin Podiatr Med Surg.

[54] Bus SA, van Netten JJ, Kottink AIR (2018). The efficacy of removable devices to offload and heal neuropathic plantar forefoot ulcers in people with diabetes: a single-blinded multicentre randomised controlled trial. Int Wound J.

[55] Health Quality Ontario (2017). Fibreglass total contact casting, removable cast walkers, and irremovable cast walkers to treat diabetic neuropathic foot ulcers: a health technology assessment. Ont Health Technol Assess Ser.

[56] Elraiyah T, Prutsky G, Domecq JP (2016). A systematic review and meta-analysis of off- loading methods for diabetic foot ulcers. J Vasc Surg.

[57] Lewis J, Lipp A (2013). Pressure-relieving interventions for treating diabetic foot ulcers. Cochrane Database Syst Rev.

[58] Morona JK, Buckley ES, Jones S, Reddin EA, Merlin TL (2013). Comparison of the clinical effectiveness of different off- loading devices for the treatment of neuropathic foot ulcers in patients with diabetes: a systematic review and meta-analysis. Diabetes Metab Res Rev.

[59] Elraiyah T, Prutsky G, Domecq JP (2016). A systematic review and meta-analysis of off-loading methods for diabetic foot ulcers. J Vasc Surg.

[60] Armstrong DG, Nguyen HC, Lavery LA, van Schie CHM, Boulton AJM, Harkless LB (2001). Off-loading the diabetic foot wound: a randomized clinical trial. Diabetes Care.

[61] Lavery LA, Higgins KR, La Fontaine J, Zamorano RG, Constantinides GP, Kim PJ (2015). Randomised clinical trial to compare total contact casts, healing sandals and a shear-reducing removable boot to heal diabetic foot ulcers. Int Wound J.

[62] Dumont I, Tsirtsikolou D, Lepage M (2010). The Ransart boot: an offloading device for every type of diabetic foot ulcer?. EWMA J..

[63] Dumont IJ, Lepeut MS, Tsirtsikolou DM (2009). A proof-of-concept study of the effectiveness of a removable device for offloading in patients with neuropathic ulceration of the foot: the Ransart boot. Diabet Med.

[64] Birke JA, Pavich MA, Patout CA, Horswell R (2002). Comparison of forefoot ulcer healing using alternative off-loading methods in patients with diabetes mellitus. Adv Skin Wound Care.

[65] Chantelau E, Breuer U, Leisch AC, Tanudjaja T, Reuter M (1993). Outpatient treatment of unilateral diabetic foot ulcers with ‘half shoes’. Diabet Med.

[66] Hissink RJ, Manning HA, van Baal JG (2000). The MABAL shoe, an alternative method in contact casting for the treatment of neuropathic diabetic foot ulcers. Foot Ankle Int.

[67] Elraiyah T, Prutsky G, Domecq JP (2016). A systematic review and meta-analysis of off-loading methods for diabetic foot ulcers. J Vasc Surg.

[68] Morona JK, Buckley ES, Jones S, Reddin EA, Merlin TL (2013). Comparison of the clinical effectiveness of different off- loading devices for the treatment of neuropathic foot ulcers in patients with diabetes: a systematic review and meta-analysis. Diabetes Metab Res Rev.

[69] Birke JA, Pavich MA, Patout CA, Horswell R (2002). Comparison of forefoot ulcer healing using alternative off-loading methods in patients with diabetes mellitus. Adv Skin Wound Care.

[70] Nubé VL, Molyneaux L, Bolton T, Clingan T, Palmer E, Yue DK (2006). The use of felt deflective padding in the management of plantar hallux and forefoot ulcers in patients with diabetes. Foot.

[71] Zimny S, Schatz H, Pfohl U (2003). The effects of applied felted foam on wound healing and healing times in the therapy of neuropathic diabetic foot ulcers. Diabet Med.

[72] Nabuurs-Franssen MH, Huijberts MS, Sleegers R, Schaper NC (2005). Casting of recurrent diabetic foot ulcers: effective and safe?. Diabetes Care.

[73] Nabuurs-Franssen MH, Sleegers R, Huijberts MS (2005). Total contact casting of the diabetic foot in daily practice: a prospective follow-up study. Diabetes Care.

[74] Jeffcoate W, Game F, Turtle-Savage V (2017). Evaluation of the effectiveness and cost- effectiveness of lightweight fibreglass heel casts in the management of ulcers of the heel in diabetes: a randomised controlled trial. Health Technol Assess.

[75] Ganguly S, Chakraborty K, Mandal PK (2008). A comparative study between total contact casting and conventional dressings in the non-surgical management of diabetic plantar foot ulcers. J Indian Med Assoc.

[76] Monteiro-Soares M, Boyko EJ, Jeffcoate W (2020). Diabetic foot ulcer classifications: a critical review. Diabetes Metab Res Rev.

[77] Game F (2016). Classification of diabetic foot ulcers. Diabetes Metab Res Rev.

[78] Monteiro-Soares M, Martins-Mendes D, Vaz-Carneiro A, Dinis-Ribeiro M (2015). Lower-limb amputation following foot ulcers in patients with diabetes: classification systems, external validation and comparative analysis. Diabetes Metab Res Rev.

[79] Forsythe RO, Ozdemir BA, Chemla ES, Jones KG, Hinchliffe RJ (2016). Interobserver reliability of three validated scoring systems in the assessment of diabetic foot ulcers. Int J Low Extrem Wounds.

[80] Jeon BJ, Choi HJ, Kang JS, Tak MS, Park ES (2017). Comparison of five systems of classification of diabetic foot ulcers and predictive factors for amputation. Int Wound J.

[81] Mills JL, Conte MS, Armstrong DG (2014). The Society for Vascular Surgery lower extremity threatened limb classification system: risk stratification based on wound, ischemia, and foot infection (WIfI). J Vasc Surg.

[82] Mathioudakis N, Hicks CW, Canner JK (2017). The Society for Vascular Surgery Wound, Ischemia, and foot Infection (WIfI) classification system predicts wound healing but not major amputation in patients with diabetic foot ulcers treated in a multidisciplinary setting. J Vasc Surg.

[83] Hicks CW, Canner JK, Karagozlu H (2018). The Society for Vascular Surgery Wound, Ischemia, and foot Infection (WIfI) classification system correlates with cost of care for diabetic foot ulcers treated in a multidisciplinary setting. J Vasc Surg.

[84] Hicks CW, Canner JK, Mathioudakis N (2018). The Society for Vascular Surgery Wound, Ischemia, and foot Infection (WIfI) classification independently predicts wound healing in diabetic foot ulcers. J Vasc Surg.

[85] Tokuda T, Hirano K, Sakamoto Y (2018). Use of the wound, ischemia, foot infection classification system in hemodialysis patients after endovascular treatment for critical limb ischemia. J Vasc Surg.

[86] Peters EJ, Lavery LA (2001). Effectiveness of the diabetic foot risk classification system of the International Working Group on the Diabetic Foot. Diabetes Care.

[87] van Acker K (2002). The choice of diabetic foot ulcer classification in relation to the final outcome. Wounds.

[88] Bravo-Molina A, Linares-Palomino JP, Vera-Arroyo B, Salmerón-Febres LM, Ros-Díe E (2018). Inter-observer agreement of the Wagner, University of Texas and PEDIS classification systems for the diabetic foot syndrome. Foot Ankle Surg.

[89] Lavery LA, Armstrong DG, Harkless LB (1996). Classification of diabetic foot wounds. J Foot Ankle Surg.

[90] Ince P, Abbas ZG, Lutale JK (2008). Use of the SINBAD classification system and score in comparing outcome of foot ulcer management on three continents. Diabetes Care.

[91] Monteiro-Soares M, Boyko EJ, Jeffcoate W, Mills JL, Russell D, Game F (2020). Diabetic foot ulcer classifications: a critical review. Diabetes Metab Res Rev.

[92] Robinson WP, Loretz L, Hanesian C (2017). Society for Vascular Surgery Wound, Ischemia, foot Infection (WIfI) score correlates with the intensity of multimodal limb treatment and patient- centered outcomes in patients with threatened limbs managed in a limb preservation center. J Vasc Surg.

[93] Schönborn M, Łączak P, Pasieka P, Borys S, Płotek A, Maga P (2022). Pro- and anti-angiogenic factors: their relevance in diabetic foot syndrome: a review. Angiology.

[94] Ostchega Y, Paulose-Ram R, Dillon CF, Gu Q, Hughes JP (2007). Prevalence of peripheral arterial disease and risk factors in persons aged 60 and older: data from the National Health and nutrition examination survey 1999-2004. J Am Geriatr Soc.

[95] Stoberock K, Kaschwich M, Nicolay SS (2021). The interrelationship between diabetes mellitus and peripheral arterial disease. Vasa.

[96] Aboyans V, Björck M, Brodmann M (2018). Questions and answers on diagnosis and management of patients with Peripheral Arterial Diseases: a companion document of the 2017 ESC Guidelines for the Diagnosis and Treatment of Peripheral Arterial Diseases, in collaboration with the European Society for Vascular Surgery (ESVS): Endorsed by: the European Stroke Organisation (ESO)The Task Force for the Diagnosis and Treatment of Peripheral Arterial Diseases of the European Society of Cardiology (ESC) and of the European Society for Vascular Surgery (ESVS). Eur Heart J.

[97] Forsythe RO, Apelqvist J, Boyko EJ (2020). Effectiveness of bedside investigations to diagnose peripheral artery disease among people with diabetes mellitus: a systematic review. Diabetes Metab Res Rev.

[98] Soyoye DO, Abiodun OO, Ikem RT, Kolawole BA, Akintomide AO (2021). Diabetes and peripheral artery disease: a review. World J Diabetes.

[99] Gazzaruso C, Montalcini T, Gallotti P (2023). Impact of microvascular complications on the outcomes of diabetic foot in type 2 diabetic patients with documented peripheral artery disease. Endocrine.

[100] Jeong D, Lee JH, Lee GB (2023). Application of extracorporeal shockwave therapy to improve microcirculation in diabetic foot ulcers: a prospective study. Medicine.

[101] Forsythe RO, Apelqvist J, Boyko EJ (2020). Effectiveness of revascularisation of the ulcerated foot in patients with diabetes and peripheral artery disease: a systematic review. Diabetes Metab Res Rev.

[102] Azhar A, Basheer M, Abdelgawad MS, Roshdi H, Kamel MF (2023). Prevalence of peripheral arterial disease in diabetic foot ulcer patients and its impact in limb salvage. Int J Low Extrem Wounds.

[103] Spiliopoulos S, Festas G, Paraskevopoulos I, Mariappan M, Brountzos E (2021). Overcoming ischemia in the diabetic foot: minimally invasive treatment options. World J Diabetes.

[104] Meloni M, Morosetti D, Giurato L (2021). Foot revascularization avoids major amputation in persons with diabetes and ischaemic foot ulcers. J Clin Med.

[105] Behroozian A, Beckman JA (2020). Microvascular disease increases amputation in patients with peripheral artery disease. Arterioscler Thromb Vasc Biol.

[106] Ferreira RC (2020). Diabetic foot. Part 1: ulcers and infections. Rev Bras Ortop.

[107] Alavi A, Sibbald RG, Nabavizadeh R, Valaei F, Coutts P, Mayer D (2015). Audible handheld Doppler ultrasound determines reliable and inexpensive exclusion of significant peripheral arterial disease. Vascular.

[108] Lv Y, Yang Z, Xiang L (2023). Lower limb arterial ischemia: an independent risk factor of sudomotor dysfunction in type 2 diabetes. Diabetes Metab Syndr Obes.

[109] Faglia E, Clerici G, Caminiti M, Quarantiello A, Curci V, Somalvico F (2010). Evaluation of feasibility of ankle pressure and foot oxymetry values for the detection of critical limb ischemia in diabetic patients. Vasc Endovascular Surg.

[110] Vriens B, D’Abate F, Ozdemir BA (2018). Clinical examination and non- invasive screening tests in the diagnosis of peripheral artery disease in people with diabetes-related foot ulceration. Diabet Med.

[111] Met R, Bipat S, Legemate DA, Reekers JA, Koelemay MJ (2009). Diagnostic performance of computed tomography angi- ography in peripheral arterial disease: a systematic review and meta-analysis. JAMA.

[112] Zou J, Zhang W, Chen X, Su W, Yu D (2022). Data mining reveal the association between diabetic foot ulcer and peripheral artery disease. Front Public Health.

[113] Faglia E, Clerici G, Clerissi J (2007). When is a technically successful peripheral angioplasty effective in preventing above-the-ankle amputation in diabetic patients with critical limb ischaemia?. Diabet Med.

[114] Noronen K, Saarinen E, Alback A, Venermo M (2017). Analysis of the elective treatment process for critical limb lschaemia with tissue loss: diabetic patients require rapid revascularisation. Eur J Vasc Endovasc Surg.

[115] Hinchliffe RJ, Brownrigg JR, Andros G (2016). Effectiveness of revascularization of the ulcerated foot in patients with diabetes and peripheral artery disease: a systematic review. Diabetes Metab Res Rev.

[116] Adam DJ, Beard JD, Cleveland T (2005). Bypass versus angioplasty in severe ischaemia of the leg (BASIL): multicentre, randomised controlled trial. Lancet.

[117] Conte MS (2010). Bypass versus Angioplasty in Severe Ischaemia of the Leg (BASIL) and the (hoped for) dawn of evidence-based treatment for advanced limb ischemia. J Vasc Surg.

[118] Taylor GI, Palmer JH (1987). The vascular territories (angiosomes) of the body: experimental study and clinical applications. Br J Plast Surg.

[119] Alexandrescu V, Sinatra T, Maufroy C (2019). Current issues and interrogations in angiosome wound targeted revascularization for chronic limb threatening ischemia: a review. World J Cardiovasc Dis.

[120] Young MJ, McCardle JE, Randall LE, Barclay JI (2008). Improved survival of diabetic foot ulcer patients 1995-2008: possible impact of aggressive cardiovascular risk management. Diabetes Care.

[121] CAPRIE Steering Committee (1996). A randomised, blinded, trial of clopidogrel versus aspirin in patients at risk of ischaemic events (CAPRIE). Lancet.

[122] Hiatt WR, Fowkes FG, Heizer G (2017). Ticagrelor versus clopidogrel in symptom- atic peripheral artery disease. N Engl J Med.

[123] Anand SS, Hiatt W, Dyal L (2022). Low-dose rivaroxaban and aspirin among patients with peripheral artery disease: a meta-analysis of the COMPASS and VOYAGER trials. Eur J Prev Cardiol.

[124] Hingorani A, LaMuraglia GM, Henke P (2016). The management of diabetic foot: a clinical practice guideline by the Society for Vascular Surgery in collaboration with the American Podiatric Medical Association and the Society for Vascular Medicine. J Vasc Surg.

[125] Hart T, Milner R, Cifu A (2017). Management of a diabetic foot. JAMA.

[126] Wukich DK, Shen W, Raspovic KM, Suder NC, Baril DT, Avgerinos E (2015). Noninvasive arterial testing in patients with diabetes: a guide for foot and ankle surgeons. Foot Ankle Int.

[127] Tehan PE, Barwick AL, Sebastian M, Chuter VH (2017). Diagnostic accuracy of resting systolic toe pressure for diagnosis of peripheral artery disease in people with and without diabetes: a cross-sectional retrospective case-control study. J Foot Ankle Res.

[128] Barshes NR, Flores E, Belkin M, Kougias P, Armstrong DG, Mills JLS (2016). The accuracy and cost-effectiveness of strategies used to identify peripheral artery disease among patients with diabetic foot ulcers. J Vasc Surg.

[129] Forsythe RO, Apelqvist J, Boyko EJ (2020). Performance of prognostic markers in the prediction of wound healing or amputation among patients with foot ulcers in diabetes: a systematic review. Diabetes Metab Res Rev.

[130] Elgzyri T, Larsson J, Nyberg P, Thörne J, Eriksson K-F, Apelqvist J (2014). Early revascularization after admittance to a diabetic foot center affects the healing probability of ischemic foot ulcer in patients with diabetes. Eur J Vasc Endovasc Surg.

[131] Sheehan P, Jones P, Caselli A, Giurini JM, Veves A (2003). Percent change in wound area of diabetic foot ulcers over a 4- week period is a robust predictor of complete healing in a 12-week prospective trial. Diabetes Care.

[132] Schaper NC, Andros G, Apelqvist J (2012). Diagnosis and treatment of peripheral artery disease in diabetic patients with a foot ulcer: a progress report of the International Working Group on the Diabetic Foot. Diabetes Metab Res Rev.

[133] Elgzyri T, Larsson J, Thörne J, Eriksson KF, Apelqvist J (2013). Outcome of ischemic foot ulcer in diabetic patients who had no invasive vascular intervention. Eur J Vasc Endovasc Surg.

[134] Stimpson AL, Dilaver N, Bosanquet DC, Ambler GK, Twine CP (2019). Angiosome specific revascularisation: does the evidence support it?. Eur J Vasc Endovasc Surg.

[135] Jongsma H, Bekken JA, Akkersdijk GP, Hoeks SE, Verhagen HJ, Fioole B (2017). Angiosome-directed revascularization in patients with critical limb ischemia. J Vasc Surg.

[136] Lavery LA, Armstrong DG, Murdoch DP, Peters EJG, Lipsky BA (2007). Validation of the Infectious Diseases Society of America’s diabetic foot infection classification system. Clin Infect Dis.

[137] Tan TW, Shih CD, Concha-Moore KC (2019). Disparities in outcomes of patients admitted with diabetic foot infections. PLoS One.

[138] Wukich DK, Johnson MJ, Raspovic KM (2022). Limb salvage in severe diabetic foot infection. Foot Ankle Clin.

[139] Lavery LA, Armstrong DG, Wunderlich RP, Mohler MJ, Wendel CS, Lipsky BA (2006). Risk factors for foot infections in individuals with diabetes. Diabetes Care.

[140] Hao D, Hu C, Zhang T, Feng G, Chai J, Li T (2014). Contribution of infection and peripheral artery disease to severity of diabetic foot ulcers in Chinese patients. Int J Clin Pract.

[141] Embil JM, Albalawi Z, Bowering K, Trepman E (2018). Foot care. Can J Diabetes.

[142] Abikhzer G, Le H, Israel O (2023). Hybrid imaging of diabetic foot infections. Semin Nucl Med.

[143] Dinh MT, Abad CL, Safdar N (2008). Diagnostic accuracy of the physical exam- ination and imaging tests for osteomyelitis underlying diabetic foot ulcers: meta-analysis. Clin Infect Dis.

[144] Lam K, Van Asten SAV, Nguyen T, La Fontaine J, Lavery LA (2016). Diagnostic accuracy of probe to bone to detect osteomyelitis in the diabetic foot: a systematic review. Clin Infect Dis.

[145] Senneville E, Morant H, Descamps D (2009). Needle puncture and transcutaneous bone biopsy cultures are inconsistent in patients with diabetes and suspected osteomyelitis of the foot. Clin Infect Dis.

[146] Dos Santos VP, Alves CAS, Queiroz AB (2019). Is there concordance between bone and tendon cultures in patients with foot tissue loss?. J Vasc Bras.

[147] Al-Balas H, Metwalli ZA, Nagaraj A, Sada DM (2023). Is fluoroscopy-guided percutaneous bone biopsy of diabetic foot with suspected osteomyelitis worthwhile? A retrospective study. J Diabetes.

[148] Senneville É, Lipsky BA, Abbas ZG (2020). Diagnosis of infection in the foot in diabetes: a systematic review. Diabetes Metab Res Rev.

[149] Aragón-Sánchez J, Lipsky BA, Lázaro-Martínez JL (2011). Diagnosing diabetic foot osteomyelitis: Is the combination of probe-to-bone test and plain radiography sufficient for high-risk inpatients?. Diabet Med.

[150] Lauri C, Tamminga M, Glaudemans A (2017). Detection of osteomyelitis in the diabetic foot by imaging techniques: a systematic review and meta-analysis comparing MRI, white blood cell scintigraphy, and FDG- PET. Diabetes Care.

[151] Waibel FW, Schöni M, Kronberger L (2022). Treatment Failures in diabetic foot osteomyelitis associated with concomitant charcot arthropathy: the role of underlying arteriopathy. Int J Infect Dis.

[152] Michelsson O, Tukiainen E (2022). Minor forefoot amputations in patients with diabetic foot ulcers. Foot Ankle Clin.

[153] Nguyen S, Wallard P, Robineau O (2022). Conservative surgical treatment for metatarsal osteomyelitis in diabetic foot: experience of two French centres. Diabetes Metab Res Rev.

[154] Truong DH, Bedimo R, Malone M (2022). Meta-analysis: outcomes of surgical and medical management of diabetic foot osteomyelitis. Open Forum Infect Dis.

[155] Tone A, Nguyen S, Devemy F (2015). Six-week versus twelve-week antibiotic therapy for nonsurgically treated diabetic foot osteomyelitis: a multicenter open-label controlled randomized study. Diabetes Care.

[156] Soldevila-Boixader L, Fernández AP, Laguna JM, Uçkay I (2023). Local antibiotics in the treatment of diabetic foot infections: a narrative review. Antibiotics.

[157] Lafontaine N, Jolley J, Kyi M (2023). Prospective randomised placebo-controlled trial assessing the efficacy of silver dressings to enhance healing of acute diabetes-related foot ulcers. Diabetologia.

[158] Lipsky BA, Berendt AR, Deery HG (2004). Diagnosis and treatment of diabetic foot infections. Clin Infect Dis.

[159] Lipsky BA, Berendt AR, Embil J, de Lalla F (2004). Diagnosing and treating diabetic foot infections. Diabetes Metab Res Rev.

[160] Peters EJ, Lipsky BA (2013). Diagnosis and management of infection in the diabetic foot. Med Clin North Am.

[161] Lavery LA, Armstrong DG, Wunderlich RP, Mohler MJ, Wendel CS, Lipsky BA (2006). Risk factors for foot infections in individuals with diabetes. Diabetes Care.

[162] Senneville E, Lipsky BA, Abbas ZG (2020). Diagnosis of infection in the foot in diabetes: a systematic review. Diabetes Metab Res Rev.

[163] Zhan LX, Branco BC, Armstrong DG, Mills JL (2015). The Society for Vascular Surgery lower extremity threatened limb classification system based on Wound, Ischemia, and foot Infection (WIfI) correlates with risk of major amputation and time to wound healing. J Vasc Surg.

[164] Lavery LA, Peters EJ, Williams JR, Murdoch DP, Hudson A, Lavery DC (2008). Reevaluating the way we classify the diabetic foot: restructuring the diabetic foot risk classification system of the International Working Group on the Diabetic Foot. Diabetes Care.

[165] Uzun G, Solmazgul E, Curuksulu H (2007). Procalcitonin as a diagnostic aid in diabetic foot infections. Tohoku J Exp Med.

[166] Park JH, Suh DH, Kim HJ, Lee YI, Kwak IH, Choi GW (2017). Role of procalcitonin in infected diabetic foot ulcer. Diabetes Res Clin Pract.

[167] Al-Shammaree SAW, Abu-ALkaseem BA, Salman IN (2017). Procalcitonin levels and other biochemical parameters in patients with or without diabetic foot complications. J Res Med Sci.

[168] Korkmaz P, Koçak H, Onbaşı K (2018). The role of serum procalcitonin, interleukin-6, and fibrinogen levels in differential diagnosis of diabetic foot ulcer infection. J Diabetes Res.

[169] Armstrong DG, Perales TA, Murff RT, Edelson GW, Welchon JG (1996). Value of white blood cell count with differential in the acute diabetic foot infection. J Am Podiatr Med Assoc.

[170] Umapathy D, Dornadula S, Rajagopalan A (2018). Potential of circulatory procalcitonin as a biomarker reflecting inflammation among South Indian diabetic foot ulcers. J Vasc Surg.

[171] Senneville E (2016). Editorial commentary: probe-to-bone test for detecting diabetic foot osteomyelitis: rapid, safe, and accurate-but for which patients?. Clin Infect Dis.

[172] Álvaro-Afonso FJ, Lazaro-Martinez JL, Aragon-Sanchez J, Garcia-Morales E, García-Álvarez Y, Molines-Barroso RJ (2014). Inter-observer reproducibility of diagnosis of diabetic foot osteomyelitis based on a combination of probe-to-bone test and simple radiography. Diabetes Res Clin Pract.

[173] Lam K, van Asten SA, Nguyen T, La Fontaine J, Lavery LA (2016). Diagnostic accuracy of probe to bone to detect osteomyelitis in the diabetic foot: a systematic review. Clin Infect Dis.

[174] van Asten SA, Jupiter DC, Mithani M, La Fontaine J, Davis KE, Lavery LA (2017). Erythrocyte sedimentation rate and C-reactive protein to monitor treatment outcomes in diabetic foot osteomyelitis. Int Wound J.

[175] Ramanujam CL, Han D, Zgonis T (2018). Medical imaging and laboratory analysis of diagnostic accuracy in 107 consecutive hospitalized patients with diabetic foot osteomyelitis and partial foot amputations. Foot Ankle Spec.

[176] Dinh MT, Abad CL, Safdar N (2008). Diagnostic accuracy of the physical examination and imaging tests for osteomyelitis underlying diabetic foot ulcers: meta-analysis. Clin Infect Dis.

[177] Cohen M, Cerniglia B, Gorbachova T, Horrow J (2019). Added value of MRI to X-ray in guiding the extent of surgical resection in diabetic forefoot osteomyelitis: a review of pathologically proven, surgically treated cases. Skeletal Radiol.

[178] Baker JC, Demertzis JL, Rhodes NG, Wessell DE, Rubin DA (2012). Diabetic musculoskeletal complications and their imaging mimics. Radiographics.

[179] Chatha DS, Cunningham PM, Schweitzer ME (2005). MR imaging of the diabetic foot: diagnostic challenges. Radiol Clin North Am.

[180] Weinstein D, Wang A, Chambers R, Stewart CA, Motz HA (1993). Evaluation of magnetic resonance imaging in the diagnosis of osteomyelitis in diabetic foot infections. Foot Ankle.

[181] Senneville E, Melliez H, Beltrand E (2006). Culture of percutaneous bone biopsy specimens for diagnosis of diabetic foot osteomyelitis: concordance with ulcer swab cultures. Clin Infect Dis.

[182] Aslangul E, M’Bemba J, Caillat-Vigneron N (2013). Diagnosing diabetic foot osteomyelitis in patients without signs of soft tissue infection by coupling hybrid 67Ga SPECT/CT with bedside percutaneous bone puncture. Diabetes Care.

[183] Letertre-Gibert P, Desbiez F, Vidal M (2017). Blood cultures after bone biopsy in diabetic foot osteomyelitis. Diagn Microbiol Infect Dis.

[184] Selva Olid A, Sola I, Barajas-Nava LA, Gianneo OD, Bonfill Cosp X, Lipsky BA (2015). Systemic antibiotics for treating diabetic foot infections. Cochrane Database Syst Rev.

[185] Dumville JC, Lipsky BA, Hoey C, Cruciani M, Fiscon M, Xia J (2017). Topical antimicrobial agents for treating foot ulcers in people with diabetes. Cochrane Database Syst Rev.

[186] Arda B, Uysal S, Tasbakan M (2017). Use of tigecycline for diabetic foot infections. Wounds.

[187] Ingram PR, Rawlins MD, Murray RJ, Roberts JA, Manning L (2016). Tigecycline use in the outpatient parenteral antibiotic therapy setting. Eur J Clin Microbiol Infect Dis.

[188] Hurlow JJ, Humphreys GJ, Bowling FL, McBain AJ (2018). Diabetic foot infection: a critical complication. Int Wound J.

[189] Lipsky BA, Dryden M, Gottrup F, Nathwani D, Seaton RA, Stryja J (2016). Antimicrobial stewardship in wound care: a position paper from the British Society for Antimicrobial Chemotherapy and European Wound Management Association. J Antimicrob Chemother.

[190] Uçkay I, Berli M, Sendi P, Lipsky BA (2019). Principles and practice of antibiotic stewardship in the management of diabetic foot infections. Curr Opin Infect Dis.

[191] Uçkay I, Kressmann B, Malacarne S (2018). A randomized, controlled study to investigate the efficacy and safety of a topical gentamicin-collagen sponge in combination with systemic antibiotic therapy in diabetic patients with a moderate or severe foot ulcer infection. BMC Infect Dis.

[192] Lauf L, Ozsvar Z, Mitha I (2014). Phase 3 study comparing tigecycline and ertapenem in patients with diabetic foot infections with and without osteomyelitis. Diagn Microbiol Infect Dis.

[193] Siami G, Christou N, Eiseman I, Tack KJ (2001). Clinafloxacin versus piperacillin-tazobactam in treatment of patients with severe skin and soft tissue infections. Antimicrob Agents Chemother.

[194] Vick-Fragoso R, Hernández-Oliva G, Cruz-Alcázar J (2009). Efficacy and safety of sequential intravenous/oral moxifloxacin vs intravenous/oral amoxicillin/clavulanate for complicated skin and skin structure infections. Infection.

[195] Abbas M, Uckay I, Lipsky BA (2015). In diabetic foot infections antibiotics are to treat infection, not to heal wounds. Expert Opin Pharmacother.

[196] Li HK, Rombach I, Zambellas R (2019). Oral versus intravenous antibiotics for bone and joint infection. N Engl J Med.

[197] Tone A, Nguyen S, Devemy F (2015). Six-week versus twelve-week antibiotic therapy for nonsurgically treated diabetic foot osteomyelitis: a multicenter open-label controlled randomized study. Diabetes Care.

[198] Senneville E, Nguyen S (2013). Current pharmacotherapy options for osteomyelitis: convergences, divergences and lessons to be drawn. Expert Opin Pharmacother.

[199] Uçkay I, Kressmann B, Di Tommaso S (2018). A randomized controlled trial of the safety and efficacy of a topical gentamicin-collagen sponge in diabetic patients with a mild foot ulcer infection. SAGE Open Med.

[200] Gardner SE, Haleem A, Jao YL (2014). Cultures of diabetic foot ulcers without clinical signs of infection do not predict outcomes. Diabetes Care.

[201] Ulcay A, Karakas A, Mutluoglu M, Uzun G, Turhan V, Ay H (2014). Antibiotherapy with and without bone debridement in diabetic foot osteomyelitis: a retrospective cohor t study. Pak J Med Sci.

[202] Senneville E, Lombart A, Beltrand E (2008). Outcome of diabetic foot osteomyelitis treated nonsurgically: a retrospective cohort study. Diabetes Care.

[203] Game FL, Jeffcoate WJ (2008). Primarily non-surgical management of osteomyelitis of the foot in diabetes. Diabetologia.

[204] Acharya S, Soliman M, Egun A, Rajbhandari SM (2013). Conservative management of diabetic foot osteomyelitis. Diabetes Res Clin Pract.

[205] Lesens O, Desbiez F, Theis C (2015). *Staphylococcusaureus*-related diabetic osteomyelitis: medical or surgical management? A French and Spanish retrospective cohort. Int J Low Extrem Wounds.

[206] Lázaro-Martínez JL, Aragón-Sánchez J, García-Morales E (2014). Antibiotics versus conservative surgery for treating diabetic foot osteomyelitis: a randomized comparative trial. Diabetes Care.

[207] Lipsky BA (2014). Treating diabetic foot osteomyelitis primarily with surgery or antibiotics: have we answered the question?. Diabetes Care.

[208] Kowalski TJ, Matsuda M, Sorenson MD, Gundrum JD, Agger WA (2011). The effect of residual osteomyelitis at the resection margin in patients with surgically treated diabetic foot infection. J Foot Ankle Surg.

[209] Atway S, Nerone VS, Springer KD, Woodruff DM (2012). Rate of residual osteomyelitis after partial foot amputation in diabetic patients: a standardized method for evaluating bone margins with intraoperative culture. J Foot Ankle Surg.

[210] Hachmöller A (2007). Outcome of minor amputations at the diabetic foot in relation to bone histopathology: a clinical audit. Zentralbl Chir.

[211] Mijuskovic B, Kuehl R, Widmer AF (2018). Culture of bone biopsy specimens overestimates rate of residual osteomyelitis after toe or forefoot amputation. J Bone Joint Surg Am.

[212] Savvidou OD, Kaspiris A, Bolia IK (2018). Effectiveness of hyperbaric oxygen therapy for the management of chronic osteomyelitis: a systematic review of the literature. Orthopedics.

[213] Doctor N, Pandya S, Supe A (1992). Hyperbaric oxygen therapy in diabetic foot. J Postgrad Med.

[214] Dissemond J, Kroger K, Storck M, Risse A, Engels P (2015). Topical oxygen wound therapies for chronic wounds: a review. J Wound Care.

[215] Game FL, Apelqvist J, Attinger C (2016). Effectiveness of interventions to enhance healing of chronic ulcers of the foot in diabetes: a systematic review. Diabetes Metab Res Rev.

[216] Galhoum AE, Trivedi V, Askar M (2021). Management of ankle charcot neuroarthropathy: a systematic review. J Clin Med.

[217] Vopat ML, Nentwig MJ, Chong ACM, Agan JL, Shields NN, Yang SY (2018). Initial diagnosis and management for acute charcot neuroarthropathy. Kans J Med.

[218] Cates NK, Wagler EC, Bunka TJ (2020). Charcot reconstruction: outcomes in patients with and without diabetes. J Foot Ankle Surg.

[219] Güven MF, Karabiber A, Kaynak G, Oğüt T (2013). Conservative and surgical treatment of the chronic Charcot foot and ankle. Diabet Foot Ankle.

[220] Hester T, Kavarthapu V (2022). Etiology, epidemiology, and outcomes of managing charcot arthropathy. Foot Ankle Clin.

[221] Yousaf S, Dawe EJC, Saleh A, Gill IR, Wee A (2018). The acute Charcot foot in diabetics: Diagnosis and management. EFORT Open Rev.

[222] Jones PJ, Davies MJ, Webb D, Berrington R, Frykberg RG (2023). Contralateral foot temperature monitoring during Charcot immo- bilisation: a systematic review. Diabetes Metab Res Rev.

[223] Macdonald A, Petrova N, Ainarkar S (2017). Thermal symmetry of healthy feet: a precursor to a thermal study of diabetic feet prior to skin breakdown. Physiol Meas.

[224] Macdonald A, Petrova N, Ainarker S (2019). Between visit variability of thermal imaging of feet in people attending podiatric clinics with diabetic neuropathy at high risk of developing foot ulcers. Physiol Meas.

[225] De Bruijn J, Hagemeijer NC, Rikken QGH (2022). Lisfranc injury: refined diagnostic methodology using weightbearing and non‐ weightbearing radiographs. Injury.

[226] Ahluwalia R, Bilal A, Petrova N (2020). The role of bone scintigraphy with SPECT/CT in the characterization and early diagnosis of stage 0 charcot neuroarthropathy. J Clin Med.

[227] Fosbøl M, Reving S, Petersen EH, Rossing P, Lajer M, Zerahn B (2017). Three‐phase bone scintigraphy for diagnosis of Charcot neuro- pathic osteoarthropathy in the diabetic foot ‐ does quantitative data improve diagnostic value?. Clin Physiol Funct Imaging.

[228] Rosenbaum AJ, DiPreta JA (2015). Classifications in brief: Eichenholtz classification of Charcot arthropathy. Clin Orthop Relat Res.

[229] Christensen TM, Gade-Rasmussen B, Pedersen LW, Hommel E, Holstein PE, Svendsen OL (2012). Duration of off-loading and recurrence rate in Charcot osteo-arthropathy treated with less restrictive regimen with removable walker. J Diabetes Complications.

[230] Chantelau E (2005). The perils of procrastination: effects of early vs. delayed detection and treatment of incipient Charcot fracture. Diabet Med.

[231] Wukich DK, Sung W, Wipf SA, Armstrong DG (2011). The consequences of complacency: managing the effects of unrecognized Charcot feet. Diabet Med.

[232] Miller R (2022). NEMISIS: Neuropathic Minimally Invasive Surgeries. Charcot midfoot reconstruction, surgical technique, pearls and pitfalls. Foot Ankle Clin.

[233] Milne TE, Rogers JR, Kinnear EM (2013). Developing an evidence-based clinical pathway for the assessment, diagnosis and management of acute Charcot Neuro-Arthropathy: a systematic review. J Foot Ankle Res.

[234] Chantelau EA, Richter A (2013). The acute diabetic Charcot foot managed on the basis of magnetic resonance imaging: a review of 71 cases. Swiss Med Wkly.

[235] Chantelau E-A, Antoniou S, Zweck B, Haage P (2018). Follow up of MRI bone marrow edema in the treated diabetic Charcot foot: a review of patient charts. Diabet Foot Ankle.

[236] Gooday C, Game F, Woodburn J (2023). A randomised feasibility study of serial magnetic resonance imaging to reduce treatment times in Charcot neuroarthropathy in people with diabetes (CADOM). J Foot Ankle Res.

[237] Petrova NL, Dew TK, Musto RL (2015). Inflammatory and bone turnover markers in a cross‐sectional and prospective study of acute Charcot osteoarthropathy. Diabet Med.

[238] Petrova NL, Moniz C, Elias DA, Buxton‐Thomas M, Bates M, Edmonds ME (2007). Is there a systemic inflammatory response in the acute charcot foot?. Diabetes Care.

[239] Folestad A, Alund M, Asteberg S (2015). IL‐17 cytokines in bone healing of diabetic Charcot arthropathy patients: a prospective 2 year follow‐up study. J Foot Ankle Res.

[240] Schara K, Stukelj R, Krek JL (2017). A study of extracellular vesicle concentration in active diabetic Charcot neuroarthropathy. Eur J Pharm Sci.

[241] Hingsammer AM, Bauer D, Renner N, Borbas P, Boeni T, Berli M (2016). Correlation of systemic inflammatory markers with radiographic stages of Charcot osteoarthropathy. Foot Ankle Int.

[242] Gough A, Abraha H, Li F (1997). Measurement of markers of osteoclast and osteoblast activity in patients with acute and chronic diabetic Charcot neuroarthropathy. Diabet Med.

[243] Moura‐Neto A, Fernandes TD, Zantut‐Wittmann DE (2012). Charcot foot: skin temperature as a good clinical parameter for predicting disease outcome. Diabetes Res Clin Pract.

[244] Schlossbauer T, Mioc T, Sommerey S, Kessler SB, Reiser MF, Pfeifer KJ (2008). Magnetic resonance imaging in early stage charcot arthrop- athy: correlation of imaging findings and clinical symptoms. Eur J Med Res.

