# Supplementary material for: Brazilian Society of Angiology and Vascular Surgery 2023 guidelines on the diabetic foot
Source: J Vasc Bras. 2024 May 17;23:e20230087. doi: 10.1590/1677-5449.202300872 (PMC11129855; doi:10.1590/1677-5449.202300872)
Supplement: Translation

# Translation: Diretrizes da Sociedade Brasileira de Angiologia e de Cirurgia Vascular sobre o pé diabético 2023

**DOI:** 10.1590/1677-5449.202300871

**Authors:** Eliud Garcia Duarte, Cicero Fidelis Lopes, Danilo Roberto Fadel Gaio, Jamil Victor de Oliveira Mariúba, Lorena de Oliveira Cerqueira, Marcos Antonio Bonacorso Manhanelli, Tulio Pinho Navarro, Aldemar Araújo Castro, Walter Jr. Boim de Araujo, Hermelinda Pedrosa, Júnio Galli, Nelson de Luccia, Clayton de Paula, Fernando Reis, Milton Sérgio Bohatch, Tércio Ferreira de Oliveira, Amanda Fernandes Vidal da Silva, Júlio Cesar Peclat de Oliveira, Edwaldo Édner Joviliano

**Affiliations:** 1 Hospital Estadual de Urgência e Emergência do Estado do Espírito Santo – HEUE, Departamento de Cirurgia Vascular, Vitória, ES, Brasil.; 2 Universidade Federal da Bahia – UFBA, Departamento de Cirurgia Vascular, Salvador, BA, Brasil.; 3 Faculdade de Engenharia de Sorocaba – FACENS, Sorocaba, SP, Brasil.; 4 Universidade Estadual Paulista “Júlio de Mesquita Filho” – UNESP, Departamento de Cirurgia Vascular, São Paulo, SP, Brasil.; 5 Universidade Vila Velha – UVV, Departamento de Cirurgia Vascular, Vila Velha, ES, Brasil.; 6 Conjunto Hospitalar de Sorocaba – CHS/Seconci, Departamento de Cirurgia Vascular, Sorocaba, SP, Brasil.; 7 Universidade Federal de Minas Gerais – UFMG, Faculdade de Medicina, Belo Horizonte, MG, Brasil.; 8 Universidade Estadual de Ciências da Saúde de Alagoas – UNCISAL, Departamento de Cirurgia Vascular, Maceió, AL, Brasil.; 9 Sociedade Brasileira de Angiologia e de Cirurgia Vascular – SBACV-PR, Curitiba, PR, Brasil.; 10 Universidade Federal do Paraná – UFPR, Hospital das Clínicas – HC, Curitiba, PR, Brasil.; 11 Hospital Regional de Taguatinga – HRT, Departamento de Cirurgia Vascular, Brasília, DF, Brasil.; 12 Universidade de São Paulo – USP, Faculdade de Medicina, Hospital das Clínicas – HC, São Paulo, SP, Brasil.; 13 Rede D’or São Luiz, Departamento de Cirurgia Vascular, São Paulo, SP, Brasil.; 14 Faculdade de Medicina de São José do Rio Preto – FAMERP, Hospital de Base, São José do Rio Preto, SP, Brasil.; 15 Sociedade Brasileira de Angiologia e de Cirurgia Vascular – SBACV-SP, São Paulo, SP, Brasil.; 16 Universidade Federal do Estado do Rio de Janeiro – UNIRIO, Departamento de Cirurgia Vascular, Rio de Janeiro, RJ, Brasil.; 17 Universidade de São Paulo – USP, Faculdade de Medicina de Ribeirão Preto – FMRP, Departamento de Cirurgia Vascular, Ribeirão Preto, SP, Brasil.

**Keywords:** pé diabético, úlcera do pé, diabetes *mellitus*

## Abstract

O pé diabético corresponde a uma interação entre fatores anatômicos, vasculares e neurológicos que representam um desafio na prática clínica. O objetivo deste trabalho foi compilar as principais evidências científicas com base em uma revisão das principais diretrizes, além de artigos publicados nas plataformas *Embase, Lilacs e PubMed*. O sistema da Sociedade Européia de Cardiologia foi utilizado para desenvolver classes de recomendação e níveis de evidência. Os temas foram divididos em seis capítulos (Capítulo 1-Prevenção de úlceras nos pés de pessoas com diabetes; Capítulo 2-Alívio da pressão de úlceras nos pés de pessoas com diabetes; Capítulo 3-Classificações das úlceras do pé diabético; Capítulo 4-Pé diabético e a doença arterial periférica; Capítulo 5-Infecção e o pé diabético; Capítulo 6-Neuroartropatia de Charcot). A versão atual das Diretrizes sobre pé diabético apresenta importantes recomendações para prevenção, diagnóstico, tratamento e seguimento dos pacientes com pé diabético, oferecendo um guia objetivo para prática médica.

## INTRODUÇÃO

Diretrizes são um conjunto de informações médicas acerca de um tema, organizadas e embasadas por evidência científica de qualidade. O intuito é fornecer informações que possam ajudar o médico na tomada de decisões diagnósticas, terapêuticas e de acompanhamento de seus pacientes^1^ . Entendendo que tais imformações necessitam de atualização constante para manter um caráter informativo e de segurança para o especialista, a Sociedade Brasileira de Angiologia e de Cirurgia Vascular (SBACV), em 2023, atualiza e incorpora novas diretrizes à sua biblioteca. O objetivo é fornecer um instrumento de trabalho que auxilie as decisões clínicas, mas preserve a autonomia de atuação do médico, prevista no Código de Ética Médica do Conselho Federal de Medicina^2^ .

Estima-se que, em 2015, 415 milhões de adultos com idade entre 20 e 79 anos tinham diabetes *mellitus* (DM) em todo o mundo, e cerca de 46,5% deles viviam em três países: China (109 milhões), Índia (69 milhões) e EUA (29 milhões)^3^ . Dez a 20 milhões de pacientes com DM estão Brasil, Rússia e México^3^ . Segundo a Federação Internacional de Diabetes, estima-se que anualmente 9,1 a 26,1 milhões de indivíduos diabéticos desenvolverão úlceras de pé diabético (UPD)^4^ . Aproximadamente 34% dos pacientes diabéticos desenvolverão úlceras ao longo da vida^4^ , com risco anual de 2,5%^5^ . O desenvolvimento da UPD está associado à mortalidade de 5% em 1 ano, chegando a 42% em 5 anos^6^ . Além do prejuízo funcional e da qualidade de vida, cerca de 20% dos pacientes que apresentam lesões de pé não cicatrizam em 1 ano após o diagnóstico^7^ , e a taxa de recorrência nesse período é de aproximadamente 40%^4^ .

O Brasil tem papel de destaque na prevalência do DM no mundo, ocupando a quarta posição no *ranking* mundial com 13 milhões de pessoas diagnosticadas e liderando o número de casos na América do Sul (Figura 1)^3,8^ .

**Figura 1:** Número total estimado de adultos (20 a 79 anos) vivendo com diabetes *mellitus* na América do Sul^3^ .

Santos et al.^9^ analisaram as complicações do pé diabético em 27 capitais no período de 10 anos (2008-2018), registrando 45.095 complicações. Houve ainda um aumento expressivo das complicações de 2008 (média de 5,68/100.000 habitantes) para 2018 (17,68/100.000 habitantes).

O BRAZUPA é um estudo transversal com avaliação de 1.455 pacientes em 19 centros brasileiros, com foco nos fatores de risco para úlcera e amputação em pacientes com DM. Destaca-se o dado alarmante da população mais jovem (média de 57 anos) que apresenta o membro em risco, em comparação com a Europa Ocidental e a América do Norte. Dos pacientes estudados, 18,6% apresentavam úlcera ativa, 25,3% tinham história prévia de úlcera e 13,7%, de amputação^10^ .

O pé diabético corresponde a uma interação entre fatores anatômicos, vasculares (macro e microangiopatia) e neurológicos que em sua alteração representam esse complexo desafio na prática diária do cirurgião vascular^3,5^ .

## OBJETIVOS

O objetivo deste trabalho foi compilar as principais evidências científicas com base na revisão das principais diretrizes e artigos relevantes e apresentar recomendações importantes para prevenção, diagnóstico, tratamento e seguimento dos pacientes com pé diabético, oferecendo um guia objetivo para prática médica.

## METODOLOGIA

Os autores realizaram uma síntese crítica dos guidelines publicados e uma revisão dos principais artigos publicados nas plataformas Embase, Lilacs PubMed/MEDLINE e Cochrane, que acrescentassem informações importantes que influenciassem a tomada de decisões e recomendações. As principais diretrizes revisadas foram as seguintes: *The management of diabetic foot: A clinical practice guideline by the Society for Vascular Surgery in collaboration with the American Podiatric Medical Association and the Society for Vascular Medicine* (2016)^11^ ; *Best practice recommendations for the Prevention and Management of Diabetic Foot Ulcers* (2017)^12^ ; *Practical Guidelines on the prevention and management of diabetic foot disease* (2019)^13^ ; *Global vascular guidelines on the management of chronic limb-threatening ischemia* (2019)^14^ ; e *Australian evidence-based guidelines for diabetes-related foot disease* (2022)^15^ .

Foram incluídas apenas publicações revisadas, seguindo o princípio da “Pirâmide de Evidências” (Tabelas 1 e 2). Múltiplos Ensaios Clínicos Randomizados (ECR) ou meta-análises de múltiplos ECRs estavam no topo da pirâmide, seguidos de ECRs únicos ou grandes estudos não randomizados (incluindo meta-análises de grandes coortes), meta-análises de pequenos estudos não randomizados, estudos observacionais e séries de casos. A opinião de especialistas ficou na base da pirâmide, enquanto relatos de casos e resumos foram excluídos.

**Tabela 1:** Classes de recomendações segundo o sistema da Sociedade Européia de Cardiologia^16^ .

**Classe de recomendação**	**Definição**	**Sugestões para utilização**
Classe I	Evidência e/ou aceitação geral que o tratamento ou procedimento é benéfico, efetivo e aplicável.	Recomendado/indicado.
Classe II	Conflito de evidência e/ou opiniões divergentes sobre aplicabilidade/eficácia do tratamento ou procedimento.	
Classe IIa	Relevância da evidência é a favor da aplicabilidade/eficácia.	Deve ser considerado.
Classe IIb	Aplicabildade/eficácia é menos estabelecida por evidência/opinião.	Pode ser considerado.
Classe III	Evidência e/ou aceitação geral que o tratamento ou procedimento não é efetivo e aplicável e podem causar danos em certos casos.	Não recomendado.

**Tabela 2:** Níveis de evidência segundo o sistema da Sociedade Européia de Cardiologia^16^ .

**Níveis de evidência.**	
Nível de evidência A	Múltiplos ensaios clínicos randomizados ou meta-análises de ensaios clínicos randomizados.
Nível de evidência B	Único ensaio clínico randomizado ou grandes estudos não randomizados.
Nível de evidência C	Opinião especialistas e/ou estudos pequenos, retrospectivos ou registros.

O sistema da Sociedade Européia de Cardiologia (ESC) foi utilizado para desenvolver classes de recomendação e níveis de evidência^16^ . A força (classe) é graduada de I a III, sendo ‘I’ a mais forte (Tabela 1). As recomendações foram desenvolvidas pelos autores designados para cada seção e todos os membros aprovaram o texto e as classificações finais.

Os principais temas foram divididos em 6 capítulos:

**Capítulo 1** - Prevenção de úlceras nos pés de pessoas com diabetes;
**Capítulo 2** - Alívio da pressão de úlceras nos pés de pessoas com diabetes;
**Capítulo 3** - Classificações das úlceras do pé diabético;
**Capítulo 4** - Pé diabético e a doença arterial periférica;
**Capítulo 5** - Infecção e o pé diabético;
**Capítulo 6** - Neuroartropatia de Charcot.

## CAPÍTULO 1. PREVENÇÃO DE ÚLCERAS NOS PÉS DE PESSOAS COM DIABETES

### Introdução

Pé diabético está entre as complicações mais graves do DM. É uma fonte de grande sofrimento e de custos financeiros, além de representar um fardo considerável para a família do paciente, profissionais de saúde, instalações e para a sociedade em geral^13^ . Estima-se que em 2015 aproximadamente US$ 673 bilhões (12% dos gastos mundiais com saúde) foram gastos no tratamento do DM e suas complicações^3^ .

A frequência de amputação é muito maior em pessoas com diabetes do que em pessoas sem a doença^17^ . Isso é especialmente verdadeiro em países desenvolvidos, como o Canadá, onde adultos com diabetes têm 20 vezes mais probabilidade de serem hospitalizados por amputação não traumática de membros inferiores do que adultos sem diabetes^18^ . Medidas preventivas, educação sobre cuidados do pé e tratamento precoce e agressivo do diabético são componentes importantes do tratamento do diabetes.

### Fisiopatologia

Embora a prevalência e o espectro do pé diabético variem em diferentes regiões do mundo, as vias de ulceração são semelhantes na maioria dos pacientes. A neuropatia periférica e a doença arterial periférica (DAP) geralmente desempenham um papel central nas complicações do pé diabético, enquanto a infecção geralmente surge como um fenômeno secundário. No entanto, todos os três componentes frequentemente têm um papel sinérgico na tríade etiológica. A neuropatia periférica está presente em aproximadamente 50% dos pacientes diabéticos, que muitas vezes desenvolvem gradualmente zonas de “alta pressão” no pé com diminuição da sensação protetora, fenômeno considerado a principal causa de UPD^19,20^ .

Em pessoas apresentando neuropatia, traumas leves (por exemplo, sapatos inadequadamente ajustados ou lesão mecânica ou térmica aguda) podem precipitar a ulceração do pé. Perda da sensibilidade protetora (PSP), deformidades adquiridas dos pés e a limitação da mobilidade articular podem resultar em carga biomecânica anormal sobre o pé. Elevado estresse mecânico em algumas áreas pode levar à formação de calosidades. O calo, então, leva a um novo aumento na carga sobre o pé, geralmente com hemorragia subcutânea e, eventualmente, ulceração da pele. Por fim, qualquer que seja a causa primária da ulceração, continuar andando com o pé insensível prejudica a cicatrização da úlcera^13,21^ . Além disso, pessoas com diabetes são mais suscetíveis a infecções devido à neuropatia, DAP, disfunção da microcirculação e imunopatia^19^ . A Figura 2 ilustra a UPD decorrente da alteração biomecânica do pé, com formação do calo e a evolução para úlcera e infecção.

**Figura 2:** Mal perfurante plantar por estresse mecânico.

Existem cinco pilares da prevenção das UPDs (Figura 3):

**Figura 3:** Os pilares na prevenção das úlceras no pé diabético^22^ .

1. identificar o pé em risco;
2. inspecionar e examinar regularmente o pé em risco;
3. educar o paciente, a família e os profissionais de saúde;
4. garantir o uso rotineiro de calçados adequados;
5. tratar fatores de risco para ulceração.

#### 1. Identificar o pé em risco

A ausência de sintomas em pessoas com diabetes não exclui a doença; elas podem apresentar neuropatia assintomática, DAP, sinais pré-ulcerativos ou mesmo uma úlcera^13,22^ . Um exame minucioso dos pés é importante para detectar a doença precocemente. A triagem para neuropatia periférica e DAP pode ajudar a identificar pacientes com risco de úlceras nos pés (classe de recomendação I, nível de evidência B). Uma história de úlceras ou amputações e mau controle glicêmico aumentam o risco^23,24^ . Recomenda-se realizar reavaliações periódicas em pacientes com o membro em risco conforme a estratificação de risco demonstrado na Tabela 3 (classe de recomendação I, nível de evidência C).

**Tabela 3:** Estratificação IWGDF 2019 do risco do pé e o intervalo de seguimento.

**Categoria**	**Risco de ulceração**	**Características**	**Frequência**
**0**	Muito baixo	Sem PSP ou DAP	1x ao ano
**1**	Baixo	PSP ou DAP	1x a cada 6-12 meses
**2**	Moderado	PSP + DAP ou PSP + deformidade do pé ou DAP + deformidade do pé	1x a cada 3-6 meses
**3**	Alto	PSP ou DAP e: História de úlcera no pé Amputação prévia DRC terminal	1x a cada 1-3 meses

PSP: perda de sensibilidade protetora; DAP: doença arterial periférica; DRC: doença renal crônica. Fonte: Bus et al.^22^

#### 2. Inspecionar e examinar regularmente o pé em risco

Deve-se examinar os pés em cada consulta de acompanhamento em busca de doença ativa (ulceração ou gangrena) (Figura 4A) e procurar lesões que aumentam o risco de ulceração, como: infecção fúngica; rachaduras e fissuras cutâneas; unhas deformadas; maceração da pele; calosidades e deformidades (dedos em martelo, dedos em garra e pé cavo) (Figura 4B e 4C) (classe de recomendação I, nível de evidência B). A avaliação da temperatura do membro pode sugerir isquemia quando frio, bem como rubor, calor aumentado e inchaço podem sugerir inflamação, como pé de Charcot agudo ou celulite^23,24^ .

**Figura 4:** Achados de exame em pé diabético. (A) História de amputação prévia e dedos em martelo; (B) Perda do arco plantar; (C) Calosidades e úlcera.

As diretrizes atuais da American Diabetes Association e Canadian Diabetes Association recomendam a triagem para neuropatia diabética para todos os pacientes no momento do diagnóstico do DM tipo-2, e após 5 anos, nos pacientes com DM tipo-1^25^ . Após, a reavaliação deve seguir a estratificação de risco IWGDF^22^ .

##### Estratificação de risco IWGDF 0

Os pacientes devem ser avaliados anualmente para identificação de fatores de risco de ulceração, como a DAP e a neuropatia^21,22^ . O Quadro 1 sugere o exame clínico básico para esses pacientes. Em geral, a PSP é causada por neuropatia diabética. Se estiver presente, geralmente é necessário obter mais informações sobre o histórico da doença e conduzir exames adicionais a respeito de suas causas e consequências (classe de recomendação I, nível de evidência B).

**Quadro 1:** Avalição básica anual dos pacientes com muito baixo risco (IWGDF 0).

Ø **Histórico:**
• Úlcera/amputação previa; claudicação intermitente/dor em repouso.
Ø **Exame vascular:**
• Palpação dos pulsos (pedioso e tibial posterior).
Ø **Exame neurológico:**
• Percepção de pressão (monofilamento 10 g);
• Percepção de vibração (diapasão 128 Hz);
Teste da sensibilidade tátil (toque leve 1-2 segundos com o indicador do examinador nas pontas dos artelhos).

Fonte: Bus et al.^22^

##### Estratificação de risco IWGDF ≥ 1

Em pacientes com DAP, PSP, deformidades, história de úlcera/amputação ou doença renal crônica (DRC) terminal, deve-se executar um exame mais abrangente, conforme o Quadro 2 (classe de recomendação I, nível de evidência B).

**Quadro 2:** Avalição dos pacientes com maior risco IWGDF ≥ 1.

Ø **Anamnese:**
• Úlcera/amputação prévia;
• Doença renal em estágio terminal;
• Educação prévia sobre os pés/conhecimento dos cuidados dos pés;
• Condições biopsiocossociais;
• Acesso sistema de saúde;
• Limitações físicas que podem dificultar o autocuidado (acuidade visual, obesidade);
• Má higiene dos pés, por exemplo, corte indevido das unhas dos pés, pés sujos, infecção fúngica; superficial ou meias sujas;
• Claudicação intermitente e em repouso;
• Alteracões de sensibilidade (alodinia, hiperalgesia, parestesias).
Ø **Tipo de calçado.**
Ø **Exame vascular:**
• palpação dos pulsos dos pés (pedioso e tibial posterior).
Ø **Tegumentar:**
• Coloração da pele, temperatura, presença de calo ou edema, sinais pré-ulcerativos.
Ø **Osso/articulação:**
• Deformidades (dedos em garra ou em martelo);
• Proeminências ósseas anormalmente grandes;
• Mobilidade articular limitada.
Ø **Exame neurológico:**
• Percepção de pressão: monofilamento de Semmes-Weinstein de 10 g;
• Percepção de vibração: diapasão de 128 Hz;
Teste a sensação tátil: toque leve por 1-2 segundos do indicador do examinador nas pontas dos pododáctilos.

Fonte: Bus et al.^22^ .

#### 3. Educação do paciente, familiar e profissionais de saúde

Embora não esteja disponível nenhuma evidência de alta qualidade do efeito dessas intervenções devido a falta de padronização e alta heterogeneidade dos estudos sobre o autocuidado na prevenção de úlceras nos pés, estas permitem que uma pessoa detecte os primeiros sinais de UPD, contribuindo, assim, para a higiene básica dos pés^26^ .

O monitoramento em domicílio da temperatura plantar uma vez por dia com um termômetro infravermelho apresenta ação preventiva quando temperaturas elevadas são observadas por dois dias consecutivos (classe de recomendação IIb, nível de evidência B)^27-29^ . Entretanto, apesar da fácil aplicabilidade, os resultados podem preocupar desnecessariamente as pessoas e afetar a sua confiança no uso dessa abordagem, além de necessitar de equipamentos calibrados disponíveis^27-29^ .

Sinais pré-ulcerativos no pé, como bolhas, fissuras ou calosidade com hemorragia subcutânea, unhas encravadas ou onicomicose parecem ser fortes preditores de ulceração futura^30^ . O profissional de saúde deve: remover calosidades abundantes; proteger as bolhas e drená-las quando necessário; tratar fissuras; tratar unhas encravadas ou infecção fúngica (classe de recomendação I, nível de evidência C)^31,32^ . Pode-se considerar a tenotomia flexora para um paciente com úlcera ou sinal pré-ulcerativo no dedo do pé, que falhe em responder ao tratamento conservador e requeira a normalização da estrutura do pé para prevenir a ulceração (classe de recomendação IIb, nível de evidência C)^33,34^ . Esses tratamentos têm potencial para causar danos quando realizados de maneira inadequada e, portanto, devem ser feitos apenas por um profissional de saúde devidamente capacitado^31,32^ .

O Quadro 3 lista as principais recomendações para o cuidado com os pacientes com pé diabético^22^ .

**Quadro 3:** Principais recomendações para o cuidado com os pacientes com pé diabético

**Educação ao paciente, familiar e profissionais de saúde**
Dieta balanceada, rica em fibras, pobre em gorduras saturadas e açúcares.
Cortar as unhas de maneira adequada (em linha reta e evitar retirar cutículas).
Evitar andar descalço ou com sapatos inadequados.
Avaliação diária dos pés (no caso de deficiência visual ou incapacidade é preciso contar com ajuda familiar).
Lavar os pés diariamente (temperatura menor que 37°C) e secar bem entre os dedos.
Hidratar bem os pés (evitar entre os dedos).
Não utilizar bolsão de água quente nos pés, evitando queimaduras.
Não tentar remover calosidades.
Ter os pés regularmente avaliados por profissionais de saúde.
Notificar o profissional de saúde se houver bolhas, cortes, arranhão, ulceração ou aumento de temperatura.
Consultar rotineiramente oftalmologista, nutricionista e endocrinologista.
Adotar medidas de segurança no controle de quedas e amenizar riscos inerentes à alteração de propriocepção.

Fonte: Bus et al.^22^ .

#### 4. Garantir o uso de calçados adequados

A avalição clínica deve seguir as características de cada peça de calçado que o paciente usa^12^ :

- ajuste: a ponta do calçado deve ser grande o suficiente para evitar pressão sobre os dedos, e o calcanhar deve ser firme, mas não muito apertado;
- estrutura: os sapatos devem ter laços/velcro. Os sapatos não devem ter costuras ou estruturas que possam resultar em atrito ou pressão;
- amortecimento;
- características gerais: os sapatos devem ser feitos de materiais respiráveis, como couro, para permitir que a umidade se dissipe;
- controle de movimento: os sapatos devem limitar a pronação excessiva (pé em eversão e achatando o arco);
- outros: o clínico deve verificar a presença de objetos estranhos dentro do sapato;
- nunca utilizar um calçado que já causou ulceração prévia.

É importante que pessoas com risco moderado ou alto de ulceração (IWGDF risco 2-3) tenham seus calçados ajustados para proteger e acomodar o formato dedos pés, com comprimento, largura e profundidade adequados. Isso pode exigir calçados, palmilhas ou órteses feitos sob medida que podem reduzir o risco de ulceração ou a recidiva (classe de recomendação I, nível de evidência B)^35-37^ .

Os benefícios do uso contínuo de calçados ou palmilhas sob moldes e com comprovado efeito de alívio da pressão superam o dano potencial^38,39^ . Por outro lado, calçados inadequados (comprimento ou largura inadequados) aumentam o risco de ulceração, de forma que deve-se garantir o ajuste adequado do calcado^22^ .

As características do calçado adequado entram-se resumidas na Figura 5. Sugere-se seguir a estratificação de risco para a escolha do tipo de calçado, conforme o Quadro 4.

**Figura 5:** Características do calçado adequado^22^ .

**Quadro 4:** Estratificação de risco para a escolha do tipo de calçado.

**Estratificação de risco IWGDF 0**	Calçados apropriados prontos para o uso.
**Estratificação de risco IWGDF 1-3**	Calçados apropriados selecionados ou confeccionados.

Fonte: Bus et al.^22^ .

#### 5. Tratar os fatores de risco para ulceração

Deve-se tratar qualquer fator de risco modificável ou qualquer sinal pré-ulcerativo. Esse tratamento deve ser repetido até que essas anomalias desapareçam e não recorram. A intervenção cirúrgica deve ser considerada em pacientes com úlceras recidivantes por causa de deformidades nos pés e que se desenvolvem apesar das medidas preventivas ideais descritas anteriormente^22^ . O Quadro 5 resume as principais medidas para o controle do risco de ulceração. A Tabela 4 resume as principais recomendações do último consenso do IWGDF^22^ .

**Quadro 5:** Resumo das medidas para o controle de risco de ulceração.

**Medidas para controle de risco de ulceração**
Tratar calosidades em excesso.
Proteger ou drenar adequadamente bolhas se necessário.
Tratar adequadamente onicodistrofias e onicomicoses.
Realizar cirurgias de reestruturação da biomecânica do pé, sobretudo se houver ulcerações recorrentes.
Notificar o profissional de saúde se houver bolhas, cortes, arranhão, ulceração ou aumento de temperatura.
Consultar rotineiramente oftalmologista, nutricionista e endocrinologista.
Adotar medidas de segurança no controle de quedas e amenizar riscos inerentes à alteração de propriocepção.

Fonte: Bus et al.^22^ .

**Tabela 4:** Recomendações do Grupo de Trabalho Internacional sobre Pé Diabético, 2019.

**Recomendações**	**Classe de recomendação e nível de evidência**
1. Examine uma pessoa com diabetes com risco muito baixo de ulceração nos pés anualmente (IWGDF risco 0) para sinais ou sintomas de perda da sensibilidade protetora e doença arterial periférica, para determinar se ela apresenta risco aumentado de ulceração nos pés.	Classe I / nível evidência B^23^ .
2. Rastreie uma pessoa com diabetes com risco de ulceração nos pés (IWGDF risco 1-3) para: histórico de ulceração no pé ou amputação de membros inferiores; diagnóstico de doença renal em estágio terminal; presença ou progressão de deformidade no pé; limitação de mobilidade articular; calosidade abundante e qualquer sinal pré-ulcerativo no pé.	Classe I / nível evidência B^23^ .
3. Repita essa triagem uma vez a cada 6-12 meses para aqueles classificados como IWGDF risco 1, uma vez a cada 3-6 meses para IWGDF risco 2 e uma vez a cada 1-3 meses para IWGDF risco 3.	Classe I / nível de evidência C^22^ .
4. Instrua uma pessoa com diabetes em risco de ulceração nos pés (IWGDF risco 1-3) para proteger seus pés evitando andar apenas com meias e sem sapatos ou com chinelos, seja em ambientes fechados ou ao ar livre. Eduque e incentive o autocuidado.	Classe I / nível de evidência C^12^ .
5. Instrua/incentive/relembre uma pessoa com diabetes em risco de ulceração (IWGDF risco 1-3) a: inspecionar diariamente toda a superfície de ambos os pés e a parte interna dos sapatos que serão calçados; lavar os pés diariamente (com secagem cuidadosa, principalmente entre os dedos); usar hidratantes para hidratar a pele seca; cortar as unhas dos pés em linha reta; e evitar o uso de agentes químicos ou emplastros ou qualquer outra técnica para remover calos ou calosidades.	Classe I / nível evidência B^26^ .
6. Considere instruir uma pessoa com diabetes em risco moderado ou alto de ulceração no pé (IWGDF risco 2-3) a automonitorar a temperatura da pele do pé 1x/dia para identificar sinais precoces de inflamação e ajudar a prevenir a úlcera. Se a diferença de temperatura estiver acima do limite entre regiões semelhantes nos dois pés e em dois dias consecutivos, instrua o paciente a reduzir suas atividades de locomoção e consultar um profissional de saúde.	Classe IIb / nível de evidência B^27-29^ .
7. Instrua uma pessoa com diabetes em risco moderado de ulceração do pé (IWGDF risco 2) ou que teve uma úlcera não plantar cicatrizada (IWGDF risco 3) a usar calçado terapêutico que se adapte ao formato dos pés e que se ajuste adequadamente, para reduzir a pressão plantar e ajudar a prevenir uma úlcera no pé. Quando uma deformidade no pé ou um sinal pré-ulcerativo estiver presente, considere prescrever calçados feitos sob medida, palmilhas ou órteses para os dedos dos pés.	Classe I / nível de evidência B^35,36^ .
7. Considere a prescrição de intervenções ortopédicas, tais como órteses de silicones para os dedos ou dispositivos ortopédicos (semi-) rígidos, para ajudar a reduzir calosidades abundantes em uma pessoa com diabetes que corre risco de ulceração nos pés (IWGDF risco 1-3).	Classe IIa / nível de evidência B^37^ .
8. Para uma pessoa com diabetes que tem uma úlcera plantar cicatrizada (IWGDF risco 3), prescreva calçado terapêutico que tenha demonstrado efeito na descarga do peso durante a caminhada, para ajudar a prevenir uma úlcera plantar recorrente no pé; além disso, encoraje o paciente a usar esse calçado em todas as ocasiões.	Classe I / nível de evidência B^38,39^ .
9. Forneça tratamento apropriado para qualquer sinal pré-ulcerativo ou calosidade abundante no pé, unhas encravadas e infecções fúngicas, para ajudar a prevenir uma úlcera no pé em uma pessoa com diabetes em risco de ulceração no pé (IWGDF risco 1-3).	Classe I / nível de evidência C.^31,32^
10. Considere a tenotomia do tendão flexor digital para prevenir ulceração em uma pessoa com diabetes e calosidade abundante ou úlcera no topo ou região distal de um dedo em martelo não rígido e que não cicatrizou com tratamento não cirúrgico.	Classe IIb / nível de evidência C^33,34^ .
11. Em uma pessoa com diabetes e úlcera plantar do antepé que não cicatrizou com tratamento não cirúrgico, considere o alongamento do tendão de calcâneo, artroplastia da articulação, uni ou panressecção das cabeças metatarsianas, artroplastia da articulação metatarsofalangeana ou osteotomia, para ajudar a prevenir a recorrência de úlcera plantar do antepé, uma vez que a úlcera ativa tenha cicatrizado.	Classe IIb / nível de evidência C^40-42^ .
12. Sugere-se não utilizar a descompressão do nervo, em detrimento dos cuidados considerados como padrão de qualidade de atendimento, para ajudar a prevenir uma úlcera no pé em uma pessoa com diabetes em risco moderado ou alto de ulceração no pé (IWGDF risco 2-3), com dor neuropática.	Classe III / nível de evidência C^22^ .
13. Considere aconselhar uma pessoa com diabetes em risco baixo ou moderado de ulceração nos pés (IWGDF risco 1 ou 2) a realizar exercícios de mobilidade com os pés com o objetivo de reduzir os fatores de risco de ulceração, ou seja, diminuir a pressão plantar e aumentar a amplitude de movimento do pé e do tornozelo com o objetivo de melhorar os sintomas de neuropatia.	Classe IIa / nível de evidência B^43-46^ .
14. Aconselhe uma pessoa com diabetes (IWGDF risco 1 ou 2) a atividade diária, como caminhada (ou seja, 1.000 passos extras/dia). Aconselhe essa pessoa a usar calçados adequados ao realizar atividades de carga sobre o pé e a monitorar a pele com frequência para sinais pré-ulcerativos ou ruptura da pele.	Classe IIb / nível de evidência B^47,48^ .

Adaptado de Bus et al.^22^ .

## CAPÍTULO 2. ALÍVIO DA PRESSÃO DE ÚLCERAS NOS PÉS DE PESSOAS COM DIABETES

A descarga de pressão pode ser alcançada com calçado temporário até que a úlcera cicatrize e os tecidos do pé se estabilizem. Botas removíveis ou não para alívio de pressão são eficazes na diminuição da pressão nas úlceras da superfície plantar^20,49,50^ .

Embora os moldes de alívio de pressão sejam eficazes em apoiar a cura de úlceras neuropáticas da superfície plantar não infectadas e não isquêmicas, a utilização requer seleção cuidadosa de pacientes e pessoal que tenha treinamento especializado para minimizar o risco de desenvolver complicações iatrogênicas^51^ .

Quando a deformidade óssea do pé impede o uso de calçados apropriados ou o descarregamento de úlceras relacionadas à pressão, consulta com um cirurgião especializado em cirurgia do pé pode ser considerada para avaliar e tratar a deformidade^52,53^ . O Fluxograma 1 resume o manejo para o alívio de pressão. A Tabela 5 resume as principais recomendações do último consenso do IWGDF^13^ .

**Fluxograma 1:** Resumo prático das medidas para alívio de pressão. Fonte: Schaper et al.^13^

**Tabela 5:** Principais recomendações do último consenso do Grupo de Trabalho Internacional sobre Pé Diabético.

**Recomendações**	**Classe de recomendação e nível de evidência**
1. Em úlcera neuropática plantar do antepé ou do mediopé, use um dispositivo de alívio da pressão não removível na altura do joelho (gesso de contato total ou botas de descarga de peso pré-fabricadas com adição de gesso ou atadura adesiva ao redor da bota) com um dispositivo de interface para o pé, como a primeira escolha de tratamento de alívio da pressão. Dispositivos não removíveis de descarga do peso oferecem benefícios para a cicatrização de uma UPD por melhor redistribuição da pressão sobre o pé e parte inferior da perna e aderência forçada.	Classe I / nível de evidência A^51,54-58^ .
2. Em ulceras neuropáticas antepé/médiopé use gesso de contato total, ou uma bota de descarga de peso removível, tornando-a irremovível, com a escolha dependente dos recursos disponíveis, habilidades do técnico, preferências do paciente e extensão da deformidade presente no pé.	Classe I / nível de evidência A^51,55,59^ .
3. Úlcera neuropática plantar do antepé ou mediopé em que um dispositivo de alívio da pressão não removível na altura do joelho é contraindicado ou não tolerado, considere o uso de bota de descarga de peso removível na altura do joelho com um dispositivo de interface para o pé como segunda escolha de tratamento de alívio da pressão para promover a cicatrização da úlcera.	Classe IIb / nível de evidência B^60,61^ .
4. Úlcera neuropática plantar do antepé ou mediopé em que uma bota de descarga de peso na altura do joelho é contraindicado ou não tolerado, use um dispositivo de alívio da pressão removível na altura do tornozelo como a terceira opção de tratamento para promover a cicatrização da úlcera.	Classe IIa / nível de evidência C^62-66^ .
5. Úlcera neuropática plantar no antepé ou mediopé, instrua o não uso de calçado terapêutico pré-fabricado ou calçado convencional, como tratamento de alívio da pressão para promover a cicatrização da úlcera, a menos que nenhum dos dispositivos mencionados acima estejam disponíveis.	Classe IIb / nível de evidência B^55,67,68^ .
6. No item 5, considere o uso de espuma feltrada com encaixe apropriado em combinação com o calçado terapêutico pré-fabricado ou calçado convencional como a quarta opção de tratamento de alívio da pressão.	Classe IIb / nível de evidência C^69-71^ .
7. Úlcera plantar neuropática do antepé ou do mediopé com infecção ou isquemia leves, considere o uso de um dispositivo de alívio da pressão não removível na altura do joelho para promover a cicatrização da úlcera.	Classe IIb / nível de evidência C^55,72,73^ .
8. Úlcera neuropática plantar do antepé ou mediopé com infecção e isquemia leves ou com infecção e isquemia moderadas, considere o uso de um dispositivo removível de alívio da pressão na altura do joelho para promover a cicatrização da úlcera.	Classe IIb / nível de evidência C^73^ .
9. Úlcera neuropática plantar do antepé ou mediopé com infecção e isquemia moderadas, ou com infecção ou isquemia graves, trate principalmente da infecção e/ou isquemia e considere o uso de um dispositivo removível para alívio da pressão que seja funcional para o paciente.	Classe IIb / nível de evidência C^55,72,73^ .
10. Úlcera neuropática no calcanhar, considere o uso de um dispositivo de alívio da pressão na altura do joelho ou outra intervenção que efetivamente reduza a pressão plantar no calcanhar e que seja tolerada pelo paciente.	Classe IIa / nível de evidência B^74,75^ .
11. Úlcera não plantar, use um dispositivo removível de alívio da pressão na altura do tornozelo, modificações dos calçados, espaçadores de dedos ou órteses, dependendo do tipo e da localização da úlcera, para promover a cicatrização.	Classe I / nível de evidência B^74^ .

Adaptado de Schaper et al.^13^ .

## CAPÍTULO 3. CLASSIFICAÇÕES DAS ÚLCERAS DO PÉ DIABÉTICO

### Introdução

Devido à complexidade dos fatores envolvidos nas UPD ainda não há uma classificação ideal para uso de rotina na prática clínica destinada às mais diversas populações do mundo^13,76^ . Trinta e sete classificações para UPD foram encontradas em uma revisão realizada por Monteiro-Soares et al.^76^ Parte dessa ampla variedade de classificações se deve aos propósitos diferentes dos autores, envolvendo principalmente o emprego de cuidados clínicos, pesquisa ou auditoria. A primeira diz respeito a uma lesão individual, membro ou paciente, com objetivo de uniformizar a comunicação entre profissionais de saúde bem como estabelecer prognóstico e orientação de condutas terapêuticas. Pesquisa e auditoria, no entanto, estão preocupadas com grupos de pacientes, membros e lesões^13,76,77^ . Atualmente, não há nenhum sistema de classificação/pontuação para análise de prognóstico individual para pessoas com UPD^13^ .

### Prática clínica

Uma classificação consensual, sistema de pontuação ou descrição de lesões nos pés no atendimento clínico de rotina auxiliam a tomada de decisões clínicas, bem como a comunicação entre os profissionais^77^ . As classificações descritivas separam os pacientes em grupos, mas não necessariamente estabelecem prognósticos. Já os sistemas de pontuação atribuem escores aos fatores envolvidos na doença e geralmente servem para estimar a gravidade e os resultados adversos^13,77^ .

O sistema utilizado na prática clínica de rotina, inclusive para a comunicação entre equipes multidisciplinares na área da saúde, deve ser simples o suficiente para ser fácil de memorizar e de aplicar, além de não necessitar de nenhum equipamento especializado^13,77^ . O sistema recomendado pelo IWGDF é SINBAD (classe de recomendação I, nível de evidência B)^13^ .

Há apenas dois sistemas de classificação que auxiliam a tomada de decisão clínica: IWGDF/IDSA e WIfI. As diretrizes atuais recomendam a utilização do sistema WIfI; entretanto, pode-se empregar o IWGDF/IDSA isoladamente na indisponibilidade de equipamentos requeridos no WIfI (classe de recomendação IIb, nível de evidência B)^13,14,77^ .

### Pesquisa Clínica

O objetivo de uma classificação de pesquisa é identificar características clínicas para a inclusão ou exclusão de pacientes em estudos. Como isso geralmente é feito em uma base experimental, apenas os centros que participam da pesquisa precisam concordar com os critérios e descrições^77^ . O sistema de classificação Perfusion, Extent, Depth, Infection and Sensation (PEDIS) refinou as definições que podem ser usadas ao considerar critérios de inclusão e exclusão para projetos de pesquisa prospectivos, e enfatiza a confiança de qualquer sistema de trabalho em cinco critérios especificados: área; profundidade; infecção; neuropatia; e isquemia^77^ .

### Auditoria clínica

A necessidade de auditoria pode variar desde simples descrições de números até buscar associações entre doenças e resultados ou resultados comparativos entre instituições diferentes. Essas comparações são fundamentais para subsidiar o gerenciamento clínico otimizado. Como os grupos estudados em cada caso podem ser grandes, qualquer classificação ou sistema de pontuação deve ser simples, inequívoca, de fácil compreensão e claramente documentada sem a necessidade de equipamentos onerosos, além de ser precisa o suficiente para ser significativa^77^ . Atualmente, o sistema SINBAD é o melhor sistema validado para auditoria e o recomendado pelo IWGDF (classe de recomendação IIa, nível de evidência C)^13,77^ . O Fluxograma 2 sumariza as classificações das UPD conforme o objetivo a ser estudado.

**Fluxograma 2:** Classificações das UPD conforme o objetivo a ser estudado. UPD = úlcera em pé diabético. Fonte: Game^77^ e Schaper et al.^13^

### Sistemas de classificações

#### Classificação SINBAD

O sistema SINBAD é simples, fácil e rápido de usar, não requer nenhum equipamento especializado além do exame clínico e contém as informações necessárias para permitir a triagem por uma equipe especializada. Seu escore é obtido por meio da soma de todos os dados importantes sobre a úlcera, que são pontuados em 0 ou 1. Os seis elementos de pesquisa da UPD são: área; profundidade; presença de sepse; DAP; desnervação; e localização. A soma pode atingir um valor máximo de seis pontos. Essa classificação foi validada para predizer a cicatrização de úlcera e prever amputações^78-80^ . SINBAD é o sistema mais amplamente validado em diferentes contextos de pesquisa de validação e com resultados amplamente consistentes^13^ . A classificação SINBAD encontra-se descrita na Tabela 6.

**Tabela 6:** Classificação SINBAD.

**Categoria**	**Definição**	**Pontuação**
1. Local	Antepé	0
	Mediopé ou retropé	1
2. Isquemia	Fluxo preservado	0
	Fluxo ausente	1
3. Neuropatia	Sensibiliade protetora preservada	0
	Sensibildiade protetora ausente	1
4. Infecção	Ausente	0
	Presente	1
5. Área	Úlcera < 1 cm^2^	0
	Úlcera ≥1 cm^2^	1
6. Profundidade	Envolvimento de pele e subcutâneo	0
	Envolvimento de musculo, tendão ou mais profundo	1
Pontuação máxima		6

Adaptado de Schaper et al.^13^ .

#### Classificação Infectious Diseases Society of America

A Classificação Infectious Diseases Society of America (IDSA) foi desenvolvida como parte da classificação PEDIS, com finalidade científica e usada a fim de identificar os pacientes com necessidade de internação hospitalar para antibioticoterapia, e posteriormente foi validada para avaliação do risco de amputações maiores e menores. A classificação do IDSA consiste em quatro níveis de gravidade para UPD e infecção, conforme a Tabela 7^13^ .

**Tabela 7:** Classificação *Infectious Diseases Society of America*.

**Quadro clínico**	**Pontuação PEDIS**	**Gravidade da infecção**
Úlcera sem exsudato ou inflamação.	1	Não infectada
Presença de ≥2 manifestações de inflamação (exsudato ou eritema, 2 Leve dor, calor ou endurecimento), celulite/eritema se estende ≤2 em torno da úlcera e a infecção é superficial limitada à pele ou tecidos subcutâneos; nenhuma outra complicação local ou doença sistêmica.	2	Leve
Sinais de infecção (como acima) em paciente sem toxemia e metabolicamente estável, mas que tem ≥1 das seguintes características: celulite se estendendo >2 cm, estrias linfangíticas disseminadas abaixo da fáscia superficial, abscesso profundo tecidual, gangrena e envolvimento de músculo, tendão, articulação ou osso.	3	Moderada
Infecção em um paciente com toxemia ou instabilidade metabólica (por exemplo, febre, calafrios, taquicardia, hipotensão, confusão, vômito, leucocitose, acidose, hiperglicemia grave ou azotemia).	4	Grave

Schaper et al.^13^ .

#### Sistema WIfI

O sistema WIfI foi publicado em 2014 e destina-se à classificação dos membros em risco. Permite a estratificação do risco de amputação maior em 1 ano e na previsão da probabilidade de que a revascularização seja necessária para cicatrização de feridas e salvamento de membros^81^ . O Global Vascular Guideline, publicado em 2019, recomenda o uso da classificação WIfI de maneira análoga ao sistema de Classificação de Tumores Malignos de estadiamento do câncer para analisar o membro em risco em pacientes^14^ . É a classificação de escolha para pacientes com UPD conforme o último Guideline publicado pelo IWGDF, também em 2019^13^ . Foi validado em vários continentes, inclusive em grupos específicos com UPD^82-84^ . É o sistema avaliador nos principais *trials* multicêntricos, como BEST-CLI, BEST-CLI2, BASIL-2 e BASIL-3, além de apresentar níveis altos de reprodutibilidade interobservador e intraobservador^85^ .

O objetivo do sistema é fornecer uma descrição mais precisa sobre os principais fatores que levam à não cicatrização e perda do membro bem como auxiliar na tomada de decisão na prática clínica^76^ . O sistema não inclui a quantificação da área da ferida ou a presença de neuropatia. Necessita de equipamentos específicos para mensuração das pressões e índices isquêmicos^76,77^ .

O sistema consiste em três componentes: W: *Wound*, avalição da extensão da ferida; I: *Ischaemia*, presença de isquemia; e fI: *foot Infection*, o grau de infecção. A extensão da ferida é avaliada por critérios aperfeiçoados da classificação da Universidade do Texas, nos EUA, permitindo ainda a correlação com a cirurgia esperada conforme o grau da ferida. A classificação de infecção (fI) é a mesma utilizada pela IDSA para a gravidade do processo infeccioso, sendo preditora do risco de amputação e validada para UPDs. O grau de isquemia (I) é avaliado pelas pressões e índices para avaliação objetiva da perfusão do pé e do potencial de cicatrização sem a revascularização^80^ . O consenso Delphi dos membros do Comitê do Global Vascular Guidelines on the Management of Chronic Limb-threatening Ischemia, da Society for Vascular Surgery, criou uma pontuação do espectro de feridas, isquemia e infecção do pé em relação ao risco de amputação de membros em 1 ano (risco muito baixo, risco baixo, risco moderado ou risco alto) e atribuiu um risco de intervenção a cada uma das possíveis combinações de pontuações^14,76^ . As Tabelas 8, 9 e 10 contemplam a classificação WIfI pormenorizada.

**Tabela 8:** Classificação de feridas na classificação de *wound, ischemia and foot infection* (WIfI).

**Pontuação da úlcera**	**UPD**	**Gangrena**
0	Sem úlcera	Sem gangrena
	Descrição clínica: dor isquêmica em repouso*	
1	Úlcera pequena e rasa na parte distal da perna ou pé; nenhum osso exposto, a menos que limitado à falange distal.	Sem gangrena
	Descrição clínica: pequena perda tecidual. Recuperável com amputação digital simples (1 ou 2 dígitos) ou cobertura de pele.	
2	Úlcera mais profunda com osso, articulação ou tendão exposto; geralmente não envolvendo o calcanhar; úlcera rasa no calcanhar, sem envolvimento do calcâneo.	Gangrena limitada aos dedos
	Descrição clínica: grande perda de tecido recuperável com múltiplas amputações digitais (≥3) ou transmetatársica padrão com cobertura de pele padrão.	
3	Úlcera extensa e profunda envolvendo o antepé e/ou mediopé; úlcera profunda em calcanhar ± com envolvimento do calcâneo.	Gangrena extensa envolvendo o antepé ou mediopé; necrose do calcanhar ± envolvimento do calcâneo.
	Descrição clínica: extensa perda de tecido recuperável apenas com uma reconstrução complexa do pé ou amputação transmetatarsiana não tradicional (Chopart ou Lisfranc); cobertura com retalhos ou manuseio complexo da úlcera devido a grande perda de tecidos moles	

* A dor isquêmica em repouso afeta o antepé e muitas vezes piora com o decúbito, enquanto é aliviada com o membro pendente, com uma duração > 2 semanas e deve estar associado a um ou mais parâmetros hemodinâmicos anormais (ITB <0,4; PST <50 mm Hg; PSD <30 mmHg; PtcO_2_<30 mm Hg e onda planas ou minimamente pulsáteis de registro de volume de pulso). Fonte: Conte et al.^14^ e Schaper et al.^13^ .

**Tabela 9:** Classificação da isquemia na classificação de *wound, ischemia and foot infection* (WIfI).

**Grau**	**ITB**	**PSD, PtcO_2_**
0	≥ 0.8	≥ 60mmHg
1	0.6-0.79	40-59mmHg
2	0.4-0.59	30-39mmHg
3	≤0.39	<30mmHg

ITB: índice tornozelo-braço; PSD: pressão sistólica do dedo; Ptco_2_: pressão transcutânea de oxigênio. Mensurar PSD ou Ptco2 se o ITB for incompressível (>1,3). A PSD deve ser medida em pacientes dom diabetes e prevalece sobre o ITB quando apresentarem valores discordantes. Se a calcificação arterial impedir o exame fidedigno do ITB ou da PSD, deve-se avaliar a isquemia por PtcO_2_, pressão de perfusão da pele ou registro do volume de pulso. Se as medições de TP e ABI resultarem em graus diferentes, TP será o principal determinante do grau de isquemia. Fonte: Conte et al.^14^ e Schaper et al.^13^ .

**Tabela 10:** Classificação de infecção do pé na classificação de *wound, ischemia and foot infection* (WIfI).

**SVS**	**Manifestação clínica da infecção**	**Grau infecção IDSA/PEDIS**
0	Ausência de sinais e sintomas de infecção	Não infectado
1	Infecção presente, definida pela presença de pelo menos dois dos seguintes itens:	Mínimo
	1.Inchaço ou endurecimento local;	
	2.Eritema >0,5 a ≤2 cm ao redor da úlcera;	
	3.Sensibilidade ou dor local;	
	4.Calor local;	
	5.Secreção purulenta (secreção espessa, opaca a branca ou sanguinolenta).	
	Infecção local envolvendo apenas a pele e o tecido subcutâneo (sem envolvimento de tecidos mais profundos e sem SIRS.	
	Excluir outras causas de resposta inflamatória da pele (trauma, gota, neuroosteoartropatia aguda de Charcot, fratura, trombose, estase venosa).	
2	Infecção local (descrito acima) com eritema >2 cm ou envolvendo estruturas mais profundas que a pele e tecidos subcutâneos (por exemplo, abscesso, osteomielite, artrite séptica, fasciíte) e sem SIRS.	Moderado
3	Infecção local (descrito acima) com os sinais de SIRS, manifestada por dois ou mais dos seguintes:	Grave*
	1. Temperatura >38 °C ou <36 °C;	
	2. Frequência cardíaca >90 batimentos/min;	
	3. Frequência respiratória >20 respirações/min ou Paco_2_ <32 mm Hg;	
	4 Contagem de leucócitos >12.000 ou <4.000 células/mm^3^ ou 10% de formas imaturas.	

IDSA: Infectious Diseases Society of America; Paco_2_: pressão parcial de dióxido de carbono arterial; PEDIS: perfusão, extensão, profundidade, infecção e sensação; SIRS: síndrome da resposta inflamatória sistêmica; SVS: Society for Vascular Surgery.

* A isquemia pode complicar e aumentar a gravidade de qualquer infecção. A infecção sistêmica pode se manifestar com outros achados clínicos, como hipotensão, confusão e vômitos, ou evidência de distúrbios metabólicos, como acidose, hiperglicemia grave e azotemia de início recente. Fonte: Conte et al.^14^ e Schaper et al.^13^ .

#### Classificação Perfusion, Extent, Depth, Infection and Sensation

A classificação PEDIS foi desenvolvida como uma classificação descritiva para uso em pesquisas, com o objetivo de definir a UPD e facilitar a comunicação entre os serviços de saúde – mas não para fins de prognóstico. Não inclui características dos pacientes nem a localização ou o número de úlceras. Sua utilização principal é em pesquisas, não excluindo a possibilidade de uso na prática clínica e nas auditorias. O PEDIS classifica as úlceras do pé diabético em cinco categorias de comprometimento: perfusão; extensão; profundidade/perda tecidual; infecção; e sensibilidade (conforme descrito na Tabela 11)^76,77^ .

**Tabela 11:** Classificação de PEDIS (perfusão; extensão; profundidade/perda tecidual; infecção).

**Categoria**	**Definição**
1. Perfusão	Grau 1: sem sintomas ou sinais de DAP;
	Grau 2: com sintomas ou sinais de DAP, mas sem isquemia crítica;
	Grau 3: com isquemia crítica.
2. Extensão/tamanho	Medida em centímetros.
3. Profundidade/perda de tecido	Grau 1: úlcera superficial pequena, não penetrante em qualquer estrutura abaixo da derme;
	Grau 2: úlcera profunda, abaixo da derme, comprometendo estruturas do subcutâneo, envolvendo fáscia, músculo e tendão;
	Grau 3: todas as partes do pé comprometidas, incluindo ossos e/ou articulações (exposição óssea, comprometimento ósseo).
4. Infecção	Grau 1: sem sintomas ou sinais de infecção;
	Grau 2: infecção envolvendo a pele e o tecido celular subcutâneo, sem envolvimento dos tecidos profundos e sem SIRS;
	Grau 3: presença de eritema > 2 cm e mais um desses itens: edema, calor local, eritema ou infecção envolvendo estruturas mais profundas como abscesso, osteomielite, artrite séptica, fasciíte. Ausência de SIRS;
	Grau 4: presença de infecção no pé com SIRS:
	1.Temperatura > 38 graus ou < 36 graus;
	2.Frequência cardíaca > 90 batimentos por minuto;
	3.Frequência respiratória > 20 incursões por minuto ou PaCO_2_ < 32 mmHg;
	5.Contagem de leucócitos > 12.000/mm ou presença > 10% de formas imaturas de leucócitos.
5. Sensibilidade	Grau 1: não detectada perda de sensibilidade no pé afetado;
	Grau 2: perda da sensibilidade do pé afetado definido por testes objetivos:
	- Teste do monofilamento 10g;
	- Teste de sensibilidade vibratória.

DAP: doença arterial periférica; SIRS: síndrome da resposta inflamatória sistêmica; PaCO_2_: pressão parcial de CO_2_ arterial. Fonte: Peters and Laveryt^86^ .

As recomendações das diretrizes internacionais para o emprego dos sistemas de classificações estão sumarizadas no Tabela 12.

**Tabela 12:** Principais recomendações do último consenso do Grupo de Trabalho Internacional sobre Pé Diabético.

Recomendações	Classe de recomendação e nível de evidência
1. Utilize o sistema SINBAD para classificação da UPD e comunicação entre equipes multidisciplinares na área da saúde.	Classe I / nível de evidência B^14,87-90^
2. Não use nenhum dos sistemas de classificação disponíveis para oferecer um prognóstico individual para uma pessoa com UPD.	Classe III / nível de evidência C^13^
3. Use o sistema IWGDF/IDSA ou Wifi na prática clínica para orientar a tomada de decisão.	Classe IIb / nível de evidência B^82-84,91,92^
4. Use o sistema SINBAD para auditoria regional/nacional/internacional que permita comparações entre instituições sobre os resultados das UPDs.	Classe IIa / nível de evidência C^78,80^

Adaptado de Schaper et al.^13^ .

## CAPÍTULO 4. PÉ DIABÉTICO COM DOENÇA ARTERIAL PERIFÉRICA

### Introdução

O diabetes é um forte fator de risco para DAP, com desenvolvimento de uma doença arterial mais difusa, multissegmentar e de predomínio distal. Esses fatores aumentam a complexidade do tratamento e estão associados a pior prognóstico dos pacientes^93^ .

### Epidemiologia

A DAP é altamente prevalente em pacientes diabéticos, afetando cerca de 25% dos pacientes com mais de 60 anos^94^ . Stoberock et al. demonstraram que a prevalência de DAP varia entre 20-50% em pacientes diabéticos, e entre 10-26% em indivíduos sem diabetes^95^ . A coexistência de DM e *DAP* (índice tornozelo-braquial [ITB] < 0,9) está associada a um aumento de duas a quatro vezes da mortalidade^96^ .

A DAP afeta até 50% dos pacientes com UPD e está associada a mau prognóstico, como amputação (5-24%), persistência (10-15%) e recorrência da úlcera, aumento de internações hospitalares, redução da qualidade de vida e aumento da mortalidade^97-100^ . Pacientes com UPD e DAP têm mortalidade estimada de 50% em 5 anos. Esse prognóstico sombrio é em grande parte atribuível à natureza sistêmica da doença arterial. Além disso, pacientes com UPD isquêmicas e neuroisquêmicas têm maior risco de mortalidade por todas as causas em comparação com pacientes com diabetes sem UPD ou com UPD neuropática^95,101^ .

### Fisiopatologia e fatores de risco DAP nos portadores de DM

A DAP se desenvolve no diabetes por uma complexa interação entre fatores hemodinâmicos, metabólicos e neuro-hormonais, que atuam por diversos mecanismos, produzindo disfunção endotelial e das células musculares lisas, anormalidades na hemostasia e na viscosidade sanguínea, inflamação crônica, acúmulo de produtos finais da glicação e estresse oxidativo^102,103^ . Os principais fatores de risco para o desenvolvimento da DAP listados em ordem de importância são: tabagismo; diabetes; hipertensão arterial; e hipercolesterolemia. Entretanto, outras associações foram descritas particularmente em portadores de DM, como o tempo de doença, níveis altos de hemoglobina glicosilada, obesidade abdominal, sexo masculino e neuropatia^98^ .

### *Manifestações macrovascular e microvascular no paciente com diabetes* mellitus

A manifestação macrovascular da doença aterosclerótica no DM é geralmente bilateral e tem predileção por artérias no segmento infrapoplíteo. O acometimento femoro-poplíteo concomitante também é comum e de igual incidência na população não diabética, enquanto o segmento ilíaco, especialmente isolado, é menos frequente^13,103^ . Também é comum pacientes diabéticos apresentarem arco plantar incompleto e maior risco de doença arterial digital com presença de pulsos tibiais palpáveis ​​que, no entanto, podem evoluir para ulcerações e gangrenas dos dedos^13^ . As lesões têm características multissegmentares com oclusões longas, pouca rede de colaterais e extensa calcificação arterial^13,101^ . Afetam indivíduos mais jovens, apresentam rápida evolução com maior perda tecidual e risco de amputação e alta taxa de recorrência após revascularização^13,103,104^ .

O sistema microvascular inclui vasos capilares e arteríolas (até ≈100 um) e é essencial para manutenção da homeostase tecidual, fornecendo oxigênio e nutrientes para cicatrização de feridas, permitindo a angiogênese, a sinalização hormonal, e ainda participa na regulação da pressão arterial sistêmica^99,105^ . A disfunção microvascular é caracterizada pelo desbalanço entre o fluxo sanguíneo e o tônus vascular, resultando em comprometimento da oferta de oxigênio ao tecido, aumento do estresse oxidativo com prejuízo da cicatrização e lesão em órgão alvo^105^ . Entre as complicações microvasculares (polineuropatia, nefropatia e retinopatia), a retinopatia parece ter maior correlação com o insucesso na cicatrização de feridas, amputação menor e mortalidade^99^ . Assim, a doença microvascular foi proposta como um fator de risco para a progressão da DAP e amputação^99^ .

Embora haja autores que defendam esses mecanismos da microangiopatia sobre a cicatrização, atualmente não há evidências concretas que suportam essas hipóteses para que sejam extrapoladas para a prática clínica. Desse modo, a DAP continua a ser a causa mais importante de déficit de perfusão do membro em pacientes com diabetes, e a doença microvascular não deve impedir a indicação de revascularização dos membros em pacientes diabéticos^13^ .

### Diagnóstico

O diagnóstico, o prognóstico e o tratamento dos pacientes diabéticos com DAP são marcadamente diferentes daqueles sem DAP. Não há sinais ou sintomas clínicos que possam excluir com precisão a DAP nos pacientes com DM^97^ . Aproximadamente 50% dos pacientes são assintomáticos, apenas 10% apresentam sintomas de claudicação intermitente e a DAP pode permanecer subclínica até que os pacientes apresentem perda tecidual grave^13,97^ . A escassez de sintomas pode estar relacionada à polineuropatia com perda da sensibilidade dolorosa^13^ . Torna-se imprescindível a identificação precoce da DAP em pacientes com UPD, pois o comprometimento arterial está associado a maior risco de úlceras que não cicatrizam, infecção e amputação bem como aumento da morbimortalidade cardiovascular^13^ . A ulceração isquêmica geralmente acomete o antepé e os dedos, porém outras áreas podem ser afetadas em pacientes com neuropatia diabética, biomecânica alterada ou deformidade do pé. Assim, todos os pacientes que apresentam sinais ou sintomas de suspeita de DAP devem ser submetidos a uma avaliação vascular completa^14^ .

A anamnese deve ser direcionada para os fatores de risco da DAP, como hipertensão, dislipidemia, tabagismo, obesidade, duração do DM e manifestações da doença aterosclerótica, como doença cerebrovascular e coronariana. Pacientes diabéticos há mais de 10 anos são mais propensos ao risco de DAP^98^ . A manifestação da doença microvascular afeta principalmente os vasos retinianos (por exemplo, amaurose), glomerulares (por exemplo, insuficiência renal) e vasos responsáveis pela nutrição de nervos periféricos (*vasa nervorum*), causando a polineuropatia sensitiva, motora e autonômica^106^ .

A dor isquêmica em repouso afeta o antepé e muitas vezes piora com o decúbito, enquanto é aliviada com o membro pendente. Apresenta duração > 2 semanas e deve estar associada a um ou mais parâmetros hemodinâmicos alterados (ITB < 0,4; pressão sistólica tornozelo [PST] < 50 mmHg; pressão sistólica do dedo [PSD] < 30 mmHg; pressão transcutânea de oxigênio [PtcO_2]_ < 30 mmHg e ondas planas ou minimamente pulsáteis de registro de volume de pulso). As medições de pressão devem ser correlacionadas com as formas de onda arteriais ao Doppler devido à calcinose medial^14^ .

Todos os pacientes com suspeita de DAP devem ser submetidos a um exame físico minucioso. Apesar de inespecíficas, características como frialdade, xerose cutânea, atrofia muscular, rarefação de fâneros e unhas distróficas são frequentemente observadas em pacientes com DAP^14,98^ . Entretanto, a polineuropatia autonômica e motora pode se manifestar com algumas dessas alterações tegumentares e musculares, respectivamente. A temperatura do pé pode estar relativamente quente devido à disautonomia e presença de *shunts* arteriovenosos, mascarando os sinais de um membro isquêmico^13^ . Recomenda-se não examinar o paciente com suspeita de DAP sentado em uma cadeira com a perna pendurada, pois isso pode levar a falsa interpretação em relação à perfusão do pé^14^ . O sinal de Buerger, palidez do pé em elevação e rubor com membro pendente e o tempo de preenchimento capilar maior que 5 segundos também são sinais de membro com insuficiência arterial^14^ .

A palpação dos pulsos dos pés faz parte da propedêutica inicial; contudo, a presença de pulsos não deve ser usada de modo isolado para a exclusão da DAP nos pacientes com DM. Mesmo com a avaliação de profissionais experientes, pulsos palpáveis podem estar presentes em membros com isquemia significativa^13^ . Uma revisão sistemática demonstrou que a palpação dos pulsos do pé apresentou sensibilidade de 55% e especificidade de 60% para o diagnóstico de DAP^97^ . Portanto, uma avaliação mais objetiva deve ser realizada em todos os pacientes com UPD^13^ .

Os testes diagnósticos à beira leito podem perder a precisão devido à neuropatia periférica, calcificação arterial e edema periférico^13^ . Não há ainda um teste ideal e não há um valor de corte definido que exclua a DAP com segurança. Recomenda-se utilizar mais de um teste em paralelo para aumentar a acurácia do diagnóstico^13,97^ . Na maioria dos portadores de UPD, se faz necessário avaliar o formato das ondas pelo Doppler das artérias distais em combinação com a PST e o ITB ou a aferição da PSD e do IDB. Assim, o diagnóstico para DAP é menos provável na presença de ITB 0,9-1,3, IDB ≥ 0,75 e onda Doppler trifásica (classe de recomendação I, nível de evidência C). No entanto, se houver incerteza ou evolução desfavorável, recomenda-se complementar a investigação com exames de imagens^13^ . Testes alternativos que também podem ser úteis na investigação das UPDs incluem: registros de oximetria de pulso (OP); registro do volume de pulso (RVP); fotopletismografia; medição da tensão transcutânea de oxigênio; e pressão de perfusão da pele (PPP)^13,14^ .

### Índice tornozelo-braquial

A neuropatia periférica (autonômica) associada à calcificação da túnica média (esclerose de Mönckeberg) das artérias distais as torna rígidas e incompressíveis, ou, ainda, pode resultar em um ITB falsamente elevado entre 0,4-1,4. Deve-se suspeitar desse fenômeno quando o ITB se encontra próximo ou dentro da faixa de normalidade, mas está associado a formas de onda monofásicas amortecidas (reconhecidas acusticamente ou visualmente em uma tela). Esses valores falsamente normais de PST e ITB foram relatados como preditores independentes de amputação maior^13,14^ . A detecção de uma onda arterial trifásica com um Doppler portátil parece fornecer evidências mais fortes para a ausência de DAP. O ITB (< 0,9) é um teste útil para a detecção de DAP. No entanto, um ITB > 0,9 não exclui a DAP^13,97^ .

O ITB pode ser mais útil para determinar o diagnóstico de DAP em pacientes com pés intactos, mas é um teste menos útil para excluir DAP em pacientes com neuropatia ou ulceração do pé. O teste apresenta sensibilidade e especificidade menores quando há UPD em comparação com pés intactos (sensibilidade de 69,5% *versus* 80,7%; especificidade 74% *versus* 91,5%), de modo que não deve ser usado isoladamente para excluir DAP em pacientes UPD^97^ .

### Pressão sistólica do dedo e índice dedo-braço

As artérias digitais são frequentemente poupadas da extensa calcificação que ocorre nas artérias tibiais, de modo que suas mensurações de fluxo refletem com maior precisão a perfusão do pé em pacientes diabéticos^14^ . Recomenda-se a mensuração PSD sempre que o PST ou o ITB falsamente elevados forem detectados ou suspeitos, principalmente quando os valores não forem concordantes com a análise acústica ou visual do formato de onda^13,14,97^ .

A PSD é medida usando um manguito específico colocado ao redor da base do hálux e conectado a um manômetro padrão. Um detector de fluxo Doppler fotopletismográfico ou de onda contínua é então usado para determinar o retorno do fluxo após desinsuflação do manguito^14^ . A PSD é geralmente 20-40 mmHg menor que a PST. IDB < 0,7 é considerado anormal, e a PSD < 30 mm Hg está associado à isquemia avançada^14^ . Uma PSD < 50 mmHg tem ótima capacidade diagnóstica em pacientes com UPD, mas uma PSD normal não foi considerada precisa o suficiente para excluir o diagnóstico^97^ . A presença de DAP é improvável se o IDB for ≥ 0,75. O IDB e a análise do formato das ondas nas artérias tibiais (medidas no maléolo medial, no dorso do pé e no meio da panturrilha para a artéria fibular) são os testes não invasivos mais úteis para selecionar os pacientes que precisam complementar o diagnóstico por imagem^13^ .

### Doppler portátil audível: análise

Um estudo de validação identificou a utilidade de um Doppler portátil audível^107^ . Esse estudo examinou 200 pacientes e 379 pernas. Todos os pacientes tiveram o ITB e a PSD mensurados em laboratórios vasculares certificados. Os sinais audíveis do Doppler manual foram sensíveis para descartar DAP (98,6% tibial posterior, 97,8% dorsal do pé), mas não específicos para o diagnóstico de DAP (37,5% tibial posterior, 30,19% dorsal do pé). O teste é simples, rápido e pode ser uma alternativa ao ITB. Os resultados do Doppler portátil audível (ITB > 0,9) são identificados como bifásicos ou trifásicos. Se uma onda monofásica ou nenhum som audível for detectado, um Doppler duplex completo do membro deve ser solicitado^12^ .

### Pressão transcutânea de oxigênio

A PtcO*_2_* permite a avalição do componente microvascular, e também pode refletir a perfusão de grandes e pequenos vasos^108^ . A sensibilidade do PtcO_2_ parece ser melhor que a PST (82% *versus* 67%) em pés intactos^109^ . Entretanto, a sensibilidade se reduz tanto para PtcO_2_ quanto PST em pacientes com UPD (28% *versus* 47%, respectivamente), com valores diagnósticos baixos nesses casos^110^ . No entanto, deve-se notar que a PtcO_2_ diminui apenas quando as reduções na perfusão arterial macrovascular são tão críticas que reduzem o suprimento de oxigênio tecidual^108^ . Esse fenômeno ocorre pelos mecanismos compensatórios microvasculares de hiperemia no membro isquêmico, mantendo uma relação curvilínea entre os valores de PtcO_2_ e as pressões locais de perfusão suficiente para manter a oxigenação tecidual normal^108^ .

### Oximetria de pulso

A OP é uma técnica atraente pelo baixo custo e aparelhos disponíveis na maioria dos ambientes de saúde, porém sua aplicabilidade ainda é limitada pela falta de evidência científica para o diagnóstico de DAP em pacientes com UPD. A medida leva em consideração a saturação do dedo do pé < 2% menor que a saturação do dedo ou aumentada em > 2% quando a perna é elevada a 12 polegadas acima do plano horizontal^97^ .

De modo geral, ainda não há evidências suficientes para recomendar um único teste à beira do leito para descartar de forma confiável a DAP em um paciente com úlcera no pé. O ITB normal (ou pulsos palpáveis) não pode descartar de forma confiável a DAP. Um segundo teste deve ser realizado, como a avaliação das formas de onda Doppler, possivelmente em combinação com medições de PSD/IDB. A OP pode se tornar uma alternativa atraente se confirmada em estudos futuros^97^ . O emprego dos testes a beira leito em pés diabéticos quanto à probabilidade do diagnóstio de DAP estão resumidos abaixo^97^ :

1. um ITB < 0,9 pode ser útil para sugerir o diagnóstico de DAP, mas um valor entre 0,9 e 1,3 não exclui DAP, principalmente em pacientes com neuropatia e/ou úlcera no pé;
2. um IDB > 0,75 torna o diagnóstico de DAP menos provável;
3. a OP (se a saturação do dedo do pé < 2% for menor que a saturação do dedo ou aumentada em > 2% quando a perna é elevada a 12 polegadas acima do plano horizontal) pode ser útil para sugerir o diagnóstico de DAP ou tornar a DAP menos provável;
4. a análise do formato de onda das tibiais (trifásicas/monofásicas) pode ser útil no diagnóstico de DAP.

Vários outros testes não invasivos, incluindo fluxometria Doppler a *laser*, PPP e pletismografia, foram usados para avaliar a perfusão do membro. No entanto, esses testes podem ser influenciados por uma variedade de fatores de confusão e não são usados rotineiramente na maioria dos laboratórios vasculares ao redor do mundo^14^ .

### Avalição do prognóstico do pé diabético ulcerado isquêmico

Devido à complexidade das lesões arteriais (predomínio infrapoplíteo, calcificação extensa, pouca rede de colaterais e lesões longas), associadas às comorbidades em pacientes com UPD e à literatura escassa, ainda não há nenhuma medida isolada consistente para a previsão da cicatrização^13^ . Os testes diagnósticos, o sistema WifI e o tempo de evolução auxiliam o médico a considerar um estudo arterial mais pormenorizado e a necessidade de revascularização (classe de recomendação I, nível de evidência B). Sobretudo, as decisões sempre devem ser ponderadas com as comorbidades do paciente^13,14^ .

### Valores prognósticos dos testes à beira leito.

As medidas de probabilidade maior de cicatrização nos pacientes com DAP e UPD são: pressão de perfusão da pele ≥ 40 mmHg; PSD ≥ 30 mmHg; ou PtcO_2_ ≥ 25 mmHg (classe de recomendação I, nível de evidência B). Qualquer um desses achados aumenta a probabilidade de cicatrização em pelo menos 25%^13^ . Já as medidas de baixa probabilidade de cicatrização e risco aumentado de amputação são: PST < 50 mmHg; ITB < 0,5; PSD < 30 mmHg; ou PtcO_2_ < 25 mmHg. Apesar de o ITB ter pouco valor no prognóstico da cicatrização, valores abaixo dos mencionados estão associados a um maior risco de amputação. Sugere-se realizar exames de imagem e considerar a revascularização precoce nesses pacientes^13,97^ .

### Sistema de classificação WIfI

O sistema WIfI foi gerado a partir do consenso de especialistas e posteriormente validado em populações com diabetes e sem diabetes^13,14,81^ . Esse sistema de classificação de úlceras, isquemia e infecção do pé (WIfI) auxilia na estimativa do risco de amputação e potencial benefício de revascularização do membro, sendo recomendado pelas diretrizes internacionais^13,14^ . A classificação encontra-se pormenorizada no Capítulo 3.

### Tempo de evolução

Recomenda-se a realização de exames de imagem e considerar revascularização em todos os pacientes com UPD e DAP, independentemente dos resultados dos testes diagnósticos, caso a úlcera progrida ou não reduza o tamanho em cerca de 50% dentro de 4-6 semanas mesmo com tratamento ideal (controle adequado da infecção, cuidado da ferida e *offloading*) e não possua outra causa provável de má cicatrização das úlceras (classe de recomendação IIa, nível de evidência C)^13,14^ .

### Comorbidades

A cicatrização está relacionada à interação do déficit de perfusão com outras características do pé e do paciente, como quantidade de perda de tecido, presença de infecção, carga mecânica sobre a úlcera e comorbidades (por exemplo, insuficiência cardíaca e doença renal terminal). O controle metabólico e infeccioso bem como a estabilidade clínica otimizada são fundamentais para o processo regenerativo^13^ . Exames de imagem e tratamentos urgentes também devem ser considerados em pacientes com DAP (mesmo com níveis de pressão mais elevados) na presença de outros preditores de mau prognóstico, incluindo infecção ou úlcera extensa^13^ .

### Exames de imagem arterial

A realização de exames de imagem arterial de alta qualidade do membro é imprescindível para determinar o melhor método de revascularização. Informações anatômicas do leito arterial devem ser obtidas para avaliar a presença, a gravidade e a distribuição de estenoses ou oclusões arteriais. O estudo detalhado das artérias infrapoplíteas e dos pés é essencial para os pacientes dom UPD^13,14^ .

#### Ultrassom duplex colorido

É o exame de escolha para o início da investigação, pelo acesso mais fácil, natureza não invasiva, baixo custo, sem meio de contraste iodado, sem radiação ionizante e pela mobilidade do aparelho^13,14^ . Toda a circulação arterial do membro inferior pode ser avaliada diretamente, desde os vasos abdominais até os do pé. Fornece detalhes anatômicos e uma avaliação fisiológica do fluxo sanguíneo. Permite determinar a localização e a extensão da doença bem como informar sobre o volume e a velocidade do fluxo. O envolvimento multissegmentar difuso, calcificação extensa, edema e perda tecidual podem dificultar a qualidade do exame^13,14^ . As principais desvantagens do ultrassom duplex colorido se devem à demora para a realização do exame, alta dependência do operador, não produzir um mapa do leito arterial e apresentar limitações para estimar o suprimento arterial colateral^14^ .

#### Angiotomografia computadorizada e angiorressonância nuclear magnética

A angiotomografia computadorizada apresenta alta sensibilidade e especificidade nos segmentos aorto-ilíacos (95% e 96%, respectivamente) e femoro-poplíteo (97% e 94%). A sensibilidade e especificidade caem um pouco para o território infrapoplíteo (95% e 91%), principalmente em casos de calcificação extensa que pode dificultar a avaliação das artérias de menor calibre^111^ . Outras desvantagens são reações alérgicas, nefropatia induzida por contraste e uso de radiação ionizante^13,14^ .

Uma das principais vantagens da angiorressonância nuclear magnética (ARNM) é o uso de um agente de contraste com baixa nefrotoxicidade (gadolíneo) e não uso de radiação ionizante. As desvantagens incluem a superestimação de estenoses, dificuldade da avaliação de reestenose intra-stent, compatibilidade com dispositivos implantados (marca-passos e desfibriladores), tempos longos de aquisição de imagem e artefatos de imagem. Seu uso é limitado em pacientes com claustrofobia e insuficiência renal grave (*clearance* da creatinina < 30 mL/min) devido ao risco de desenvolvimento de fibrose nefrogênica sistêmica. Os novos agentes não gadolínicos, como as partículas paramagnéticas ultrapequenas de óxido de ferro, podem ser agentes alternativos e mais seguros em pacientes com função renal comprometida^13,14^ .

Para avalição das doenças arteriais nos vasos da perna/pé, tanto a ATC quanto a ARNM podem não obter imagens completas com resolução suficiente para planejamento terapêutico. Assim, a diretriz internacional publicada em 2019 do Global Vascular Guidelines on the Management of Chronic Limb-threatening Ischemia, endossada pela Society for Vascular Surgery, European Society for Vascular Surgery e World Federation of Vascular Societies, não recomenda a ATC para o estudo detalhado da doença infrapoplítea, que deve ser investigada por arteriografia diagnóstica completa, incluindo o tornozelo e o pé^13,14^ .

#### Arteriografia digital por subtração

A arteriografia digital por subtração ainda é considerada o padrão-ouro para imagens arteriais, em razão de sua alta resolução espacial, principalmente para o território infrapoplíteo. Tem a vantagem de permitir o tratamento durante o procedimento, mas a desvantagem do uso de contraste iodado e pela característica invasiva com possíveis complicações relacionadas à punção arterial^13,14^ . A angiografia com CO_2_ pode ser usada em pacientes com alergia ao contraste iodado e em indivíduos com DRC grave. Apresenta qualidade de imagem inferior à angiografia iodada e há degradação progressiva do desempenho da imagem ao longo da perna, mas ainda pode fornecer imagens diagnósticas úteis ou mesmo reduzir o volume de contraste iodado^13,14^ . O Fluxograma 3 sumariza o emprego dos exames de imagem nos pacientes com plano de revascularização.

**Fluxograma 3:** Emprego dos exames de imagem nos pacientes com plano de revascularização. UPD = úlcera em pé diabético; PST = pressão sistólica tornozelo; ITB = índice tornozelo-braquial; PSD = pressão sistólica do dedo; PtcO_2_ = pressão transcutânea de oxigênio; WifI: sistema de classificação (W) ferida, (I) isquemia e (FI) infecção do pé. Fonte: Conte et al.^14^ , Forsythe et al.^97^ e Schaper et al.^13^

### Revascularização do membro

As indicações para o tratamento da DAP em diabéticos são semelhantes aos pacientes não diabéticos: claudicação limitante, dor em repouso e perda tecidual associada a úlceras que não cicatrizam e gangrenas^14^ . Entre os pacientes com UPDs isquêmicos, aproximadamente 25% não possuem opções para revascularização, com uma taxa de 25-50% de amputação maior devido à revascularização malsucedida do membro ou não serem candidatos a revascularização^101,112^ . Esses indivíduos são geralmente caracterizados por uma doença arterial multinível, com alto envolvimento das artérias do pé (aproximadamente em 75% dos casos)^112^ . Faglia et al. demonstraram que em pacientes diabéticos com ausência de uma artéria tibial patente no final da angioplastia resultou em uma taxa de amputação de 62%, em comparação com 1,7% em pacientes com pelo menos uma artéria patente para o pé^113^ .

O objetivo da revascularização é restaurar o fluxo sanguíneo direto para pelo menos uma das artérias do pé, de preferência a artéria que supre a região anatômica da úlcera. A perfusão é o principal parâmetro para cicatrização da UPD, seleção do nível de amputação e salvamento do membro. Ressalta-se que o atraso em mais de 2 semanas desde o diagnóstico da UPD até a revascularização aumenta substancialmente o risco de perda de membro^114^ . No entanto, deve-se ponderar a indicação de revascularização para cada caso, pois até 50% dos pacientes com UPD e DAP sem revascularização podem cicatrizar as úlceras^13^ .

Com o diagnóstico de DAP estabelecido, a necessidade da revascularização vai se basear no conceito do PLAn, no qual: P – *patient risk* avalia o risco do paciente, L – *limb threat severity*, a severidade da ameaça ao membro (classificação WIfI), e An – *anatomic pattern of disease,* que avalia a extensão da doença arterial segundo a classificação do Global Anatomic Staging System (GLASS). O PLAn auxilia na escolha do tratamento, desde a amputação primária ou revascularização, e serve como ferramenta para ajudar a definir a melhor opção de revascularização (cirurgia aberta ou endovascular)^14^ .

Uma revisão sistemática mostrou que a taxa de salvamento de membro varia de 70-90% para os pacientes submetidos à revascularização (seja aberta ou endovascular), com mais de 60% das úlceras cicatrizadas em 1 ano^115^ . Mesmo em pacientes com anatomia arterial desfavorável submetidos a *bypass* ultradistal ou angioplastia inframaleolar, as taxas de salvamento do membro foram relatadas como 86% e 77% em 1 ano^101^ .

As evidências atuais não permitem estabelecer a superioridade de uma técnica sobre a outra (endovascular, aberta ou híbrida). Além disso, não há grandes estudos randomizados abordando os métodos mais apropriados de revascularização especificamente para pacientes com UPD e DAP^101^ . As decisões devem levar em consideração os fatores individuais, como distribuição morfológica da doença arterial periférica, disponibilidade de veia autógena, comorbidades e *expertise* do cirurgião (classe de recomendação I, nível de evidência B)^13,14,101^ .

O estudo Bypass *versus* Angioplasty in Severe Ischemia of the Leg (BASIL)^116^ comparou a intervenção endovascular com a cirurgia aberta. A morbidade perioperatória foi maior com a cirurgia, mas a sobrevida global e livre de amputação em 1 ano foram semelhantes entre os grupos. No entanto, no intervalo de 2 anos, a cirurgia foi associada a um risco menor de amputação e morte. Concluiu-se que a angioplastia deve ser usada primeiro para pacientes com expectativa de vida ≤ 2 anos, e que o *bypass* é preferível quando o enxerto venoso está disponível. Entretanto, o estudo incluiu uma pequena proporção de pacientes (42%) com DM, sem análise de subgrupo, além de não ser focado para pacientes com úlcera. Portanto, não podemos extrapolar esses achados para os pacientes com UPD e DAP^117^ .

A revascularização não deve ser realizada se não houver uma chance realista de cicatrização da úlcera, ou quando a evolução para amputação for inexorável (classe de recomendação III, nível de evidência C). Os pacientes não candidatos para a revascularização são aqueles com: fragilidade importante; baixa expectativa de vida; estado funcional ruim; acamados; grande área de destruição de tecido que torna o pé funcionalmente inviável; e incapacidade de reabilitação após revascularização. Nesses casos, procede-se a amputação primária ou uma abordagem paliativa que deve ser tomada em conjunto com o paciente e uma equipe multidisciplinar^13,14^ .

#### Revascularização guiada por angiossomo

Em 1987, Taylor and Palmer^118^ propuseram o conceito de angiossoma, no qual uma artéria nutre uma unidade tridimensional de tecido. Os três vasos principais (tibial posterior, fibular e tibial anterior) nutrem áreas específicas da perna e do pé (Figura 6). Dessa forma, visa-se a identificar e revascularizar a artéria que nutre a área específica de perda de tecido (revascularização direta), restaurando o fluxo pulsátil diretamente para a área isquêmica, tornando mais provável a cicatrização. Alternativamente, a terapia não direcionada por angiossoma (revascularização indireta) adota a abordagem do “melhor vaso”, em que a artéria-alvo mais adequada é escolhida, independentemente de estar relacionada à área de perda de tecido, e, portanto, o fluxo sanguíneo é restaurado para a área por colaterais^13,101,119^ .

**Figura 6:** Ilustração dos angiossomas do pé face anterior e face posterior^119^ . (1) Angiossoma comunicante anterior (da artéria fibular); (2) Angiossoma *Dorsalis Pedis* (da artéria tibial anterior); (3) Angiossoma plantar lateral (da artéria tibial posterior); (4) Angiossoma calcâneo lateral (da artéria fibular); (5) Angiossoma calcâneo medial (da artéria tibial posterior); (6) Angiossoma plantar medial (da artéria tibial posterior).

Como pacientes com DM têm uma pobre rede de circulação colateral e normalmente não possuem um arco pedioso completo ou colaterais da artéria fibular para pé, parece intuitivo que a revascularização dirigida por angiossomas pode ser mais eficaz. Assim, o consenso atual é que a revascularização direcionada ao angiossoma deve ser realizada quando possível (RD) (classe de recomendação IIb, nível de evidência C)^13,101^ . No entanto, devido à falta de padronização de definições e erros metodológicos, a eficácia científica robusta do conceito angiossômico em pacientes com diabetes é desconhecida^13^ .

A angioplastia bem-sucedida de um ou mais vasos ocluídos não é o mesmo que um procedimento clinicamente bem-sucedido, e, antes de o procedimento terminar, o fluxo sanguíneo para a área da úlcera deve ser avaliado. Se possível, a abertura de múltiplas artérias pode ser útil, desde que pelo menos uma alimente a área isquêmica diretamente^13^ . A eficácia de um procedimento de revascularização deve ser avaliada preferencialmente com aferições objetivas de perfusão para ser considerada eficaz, tais como: pressão de perfusão cutânea > 40 mmHg; PSD > 30 mmHg; ou PtcO_2_ > 25 mmHg. Como a tensão de oxigênio na pele aumenta progressivamente em um período de várias semanas após uma angioplastia transluminal percutânea bem-sucedida, as medições de PtcO_2_ devem ser realizadas preferencialmente em pelo menos 1-3 semanas após o procedimento^13^ .

O desbridamento extenso ou a amputação parcial do pé não deve ser realizada até que o membro seja revascularizado em pacientes com isquemia avançada, perda tecidual grave e sem infecção. Já em pacientes com infecção grave, principalmente com síndrome da resposta inflamatória sistêmica, deve-se realizar a drenagem imediata antes da revascularização, a fim de controlar a sepse. Assim que a sepse estiver controlada e o paciente apresente estabilidade clínica, procede-se o estudo arterial e a resvascularização do membro o mais precocemente possível. Após controle infeccioso e restauração do fluxo sanguíneo, realiza-se a cirurgia definitiva para tornar o membro funcional^13^ . A revascularização é mais uma etapa no tratamento da UPD, e, após o procedimento, deve-se garantir um seguimento multidisciplinar como parte de um plano de cuidados abrangente, que aborde o tratamento imediato de infecção, desbridamento da úlcera, descarga biomecânica, controle glicêmico e tratamento de comorbidades^13^ .

Deve-se realizar tratamento intensivo para redução do risco cardiovascular nesses pacientes, incluindo a interrupção do tabagismo, tratamento da hipertensão, controle da glicemia, tratamento com estatina e antiagregante plaquetário em dose baixa^13,15^ . Young et al. mostraram que a adoção de uma conduta agressiva no manejo de risco cardiovascular reduziu a mortalidade de pacientes com UPD neuroisquêmicos (mortalidade em 5 anos de 58% para 36%, com redução do risco relativo de 38%)^120^ . Não há evidências específicas que apoiem o agente antiplaquetário mais apropriado nem a combinação dos novos anticoagulantes (DOAC) em pacientes com DAP e UPD^13^ . Alguns trabalhos demonstram redução do desfecho cardiovascular em pacientes com DAP em uso do clopidogrel em relação ao ácido acetilsalicílico^121,122^ . Uma metanálise dos estudos COMPASS e VOYAGER demonstrou que a rivaroxabana em dose baixa associada à aspirina foi superior à aspirina utilizada isoladamente na redução dos desfechos cardiovasculares e dos membros, ao custo do aumento relativo de sangramentos maiores não fatais. Conclui-se que a combinação seja benéfica em pacientes com DAP. Entretanto, o número de pacientes diabéticos era limitado (40-47%), poucos tinham membro em risco (2,8-31,8%), nenhuma informação foi fornecida sobre a presença de úlcera no membro e não houve análise de subgrupo voltado para UPD e DAP^123^ . O Fluxograma 4 resume a abordagem dos pacientes com UPD e DAP, e a Tabela 13 resume as principais recomendações para o manejo dos pacientes diabéticos com DAP.

**Fluxograma 4:** Resumo da abordagem em pacientes com UPD e DAP. UPD = úlcera em pé diabético; PST = pressão sistólica tornozelo; ITB = índice tornozelo-braquial; PSD = pressão sistólica do dedo; IDB = índice dedo-braquial; PtcO_2_ = pressão transcutânea de oxigênio; PPP = pressão de perfusão da pele; WifI: sistema de classificação (W) ferida, (I) isquemia e (FI) infecção; GLASS: sistema de classificação anatômica, em inglês *global limb anatomic staging system*; PLAn = método de decisão de tomada clínica em três passos – P - *patient risk*, L - *limb threat severity* e An - *anatomic pattern of disease*; IWGDF = “International Working Group on the Diabetic Foot”. Fonte: Conte et al.^14^ , Forsythe et al.^97^ , Schaper et al.^13^ e Chuter et al.^15^ .

**Tabela 13:** Recomendações para o manejo dos pacientes diabéticos com DAP.

**Recomendações**	**Classe de recomendação e nível de evidência**
1. Investigue anualmente a DAP (anamnese e palpação pulsos) em todos os pacientes com DM, mesmo na ausência de ulceração nos pés.	Boa prática clínica^124,125^
2. Complemente a investigação de DAP e UPD com a avalição do formato de onda pelo Doppler em combinação com a PST e ITB ou PSD e IDB. O ITB 0,9-1,3, IDB ≥0,75 e a presença de ondas trifásicas tornam o diagnóstico de DAP menos provável.	Classe I/nível de evidência C^126-128^
3. Realize pelo menos um dos seguintes testes diagnósticos em paciente com UPD e DAP: PPP ≥40 mmHg; PSD ≥30 mmHg; ou PtcO_2_ ≥25 mmHg. Qualquer um deles aumenta a probabilidade de cicatrização em pelo menos 25%.	Classe I/nível de evidência B^97,129^
4. Estratifique o risco de amputação e o benefício da revascularização em pacientes com UPD e DAP pelo sistema de classificação WIfI.	Classe I/nível de evidência B^13,81,117^
5. Sempre considere imagens vasculares urgentes e revascularização em UPD quando: 1) PST <50 mmHg, ITB <0,5, PSD <30 mmHg ou PtcO_2_ <25 mmHg; 2) úlcera não estiver cicatrizando ou não cicatrizar em 4-6 semanas, apesar do tratamento ideal.	Classe IIa/nível de evidência C^114,130,131^
6. Não presuma que a microangiopatia diabética é a causa da má cicatrização em pacientes com UPD.	Classe III/nível de evidência C^114,130,131^
7. Realize o estudo arterial de todo o membro com imagens detalhadas das artérias infrapoplíteas no planejamento cirúrgico com: ultrassom duplex colorido, angiotomografia, angiorressonância ou arteriografia digital.	Classe I/nível de evidência C^14,132^
8. Trata os pacientes com UPD e DAP em caráter de urgência, pois eles apresentam risco alto de perda do membro.	Classe I/nível de evidência B^114,115^
9. Evite a revascularização quando houver risco-benefício desfavorável para a probabilidade de sucesso do procedimento.	Classe III/nível de evidência C^130,133^
10. Restaure o fluxo sanguíneo direto guiado pelo angiossoma quando possível e realize uma avalição objetiva da perfusão para avaliar a eficácia.	Classe IIb/nível de evidência C^134,135^
11. Escolha o método de revascularização com base em fatores individuais, complexidade anatômica da DAP, disponibilidade de veia autógena, comorbidades do paciente e experiência do serviço.	Classe I/nível de evidência B^14,96,115^
12. Certifique-se do seguimento multidisciplinar dos pacientes após a revascularização e promova o tratamento intensivo para redução do risco cardiovascular com DAP e UPD (interrupção do tabagismo, tratamento da hipertensão, controle da glicemia, administração de estatina e antiagregantes plaquetários.	Classe I / nível de evidência B^13,14,96^

DAP: doença arterial periférica; DM: diabetes mellitus; UPD: úlcera em pé diabético; PST: pressão sistólica tornozelo; ITB: índice tornozelo-braquial; PSD: pressão sistólico no dedo; IDB: índice dedo-braquial; PPP: pressão de perfusão da pele; PtcO_2_: pressão transcutânea de oxigênio; WifI: sistema de classificação (W) ferida, (I) isquemia e (FI) infecção. Fonte: Forsythe et al.^97^ , Schaper et al.^13^ e Chuter et al.^15^ .

## CAPÍTULO 5. DIAGNÓSTICO E TRATAMENTO DE INFECÇÃO NOS PÉS DE PESSOAS COM DIABETES

### Introdução

As infecções do pé diabético (IPDs) são as complicações mais frequente que levam à internação no paciente com diabetes, sendo responsável pela maior causa de amputação nesses pacientes^136,137^ . Até 17% dos pacientes portadores de uma UPD infectada evoluem para amputação em 1 ano, enquanto 10% apresentam reinfecção após cicatrização da ferida^137^ . Considerando-se apenas infecções agudas, as taxas de amputação menores necessárias para tratamento podem chegar a 40% dos casos^138^ . Dessa forma, se faz necessária uma abordagem sistemática e precisa com o intuito de realizar diagnóstico precoce das IPDs, reduzir a morbidade e melhorar os desfechos^13^ .

### Diagnóstico

A IPD foi definida clinicamente pelo IWGDF como “a presença de manifestação de um processo inflamatório em qualquer tecido abaixo dos maléolos em uma pessoa com diabetes”. A despeito da definição, ressalta-se a possibilidade de ausência de processo inflamatório característico, principalmente nos pacientes com DAP associada. Assim, a avaliação de fatores predisponentes à infecção, como úlcera profunda, recorrente, de longa data ou de etiologia traumática, DRC e alterações da imunidade associadas ao diabetes, pode auxiliar na suspeita diagnóstica^139,140^ . Avaliar as alterações de temperatura e edema também pode ser útil no diagnóstico, já que essas estão presentes em processos infecciosos, podendo ser resultado de celulite subjacente ou processos inflamatórios relacionados à artropatia de Charcot^141^ .

Apesar de a maioria das IPDs se tratar de infecções superficiais, as infecções profundas têm um potencial devastador à medida que, através das fáscias e tendões dos compartimentos profundos do pé, podem se disseminar de forma ascendente. Nesses casos podem gerar infecções rapidamente progressivas, determinando aumento de pressão dos compartimentos internos com o desenvolvimento de síndrome compartimental e consequente necrose por alteração de perfusão tecidual^13^ .

Ao avaliar uma úlcera no pé de um paciente com diabetes, o profissional deve investigar a presença de infecção. A diferenciação clínica entre uma infecção de partes moles, osteoartropatia neuropática diabética e osteomielite é um desafio diagnóstico e exige uma propedêutica detalhada. Dor, febre e marcadores inflamatórios elevados podem ocorrer e se sobrepor em todas essas condições^142^ . Nesse momento, deve-se classificá-la de acordo com a IWGDF/IDSA, sistema já validado para utilização na estratificação infecciosa, que, inclusive, encontra-se incluído na escala mais frequentemente utilizada para classificação do pé diabético, o sistema WIfI (classe de recomendação I, nível de evidência B)^13,136^ . As classificações encontram-se detalhadas no Capítulo 3.

Mediante avaliação da gravidade do processo infeccioso, sugere-se considerar internação hospitalar em infecções graves, complexas, com indicação de abordagem cirúrgica, principalmente em pacientes com comorbidades importantes e presença de DAP associada. A avaliação laboratorial complementar pode ser utilizada para determinar parâmetros de gravidade bem como auxiliar o diagnóstico infeccioso no âmbito de um exame físico inconclusivo. Incluída na classificação da IWGDF/ISDA, a leucocitose está associada à gravidade do processo infeccioso. Ademais, estão indicados exames laboratoriais marcadores de infecção, como a velocidade de hemossedimentação (VHS), proteína C reativa (PCR) e dosagem de procalcitonina (classe de recomendação IIb, nível de evidência C). Os dois últimos apresentam maior sensibilidade e elevação mais precoce, enquanto a VHS, quando em valores superiores a 70 mm/h, tem associação à infecção óssea^13^ .

Parâmetros adicionais podem ser avaliados para definição da presença de osteomielite nas IPDs. Existem dois preditores clínicos de osteomielite: o tamanho e a profundidade da úlcera e o teste de sondagem óssea – *probe to bone test*. Uma úlcera profunda com exposição visual óssea tem 100% de especificidade para o diagnóstico de osteomielite, e a sensibilidade é de apenas 32%. A sensibilidade aumenta para 52% quando a área da úlcera é maior que 2 cm, mantendo uma alta especificidade de 92%^143^ . O teste de sonda óssea consiste na introdução de uma sonda romba de forma estéril e suave no ferimento, e é positivo quando a sonda atinge o osso ou o espaço articular. Um teste positivo é indicativo de infecção óssea com sensibilidade de 87% e especificidade de 83%^144^ . O padrão-ouro para diagnóstico da osteomielite é a biópsia óssea de forma asséptica (via percutânea ou cirúrgica) com cultura ou histologia positiva, sendo essa também a única forma de isolar o patógeno específico causador da infecção para realização do tratamento antibiótico guiado. Apesar de factível, a biópsia óssea deve ser considerada em casos nos quais há possibilidade de resistência, uso de tratamentos anteriores ou falha do tratamento antimicrobiano atual^142^ .

Já as culturas de tecidos moles devem ser coletadas de todas as feridas e de forma asséptica (curetagem ou biópsia) para direcionamento do tratamento. Quando obtidas de tecidos profundos com crescimento de patógeno único, podem sugerir etiologia da infecção óssea associada, porém há que se considerar que estudos demonstram correlação da cultura de tecidos moles e óssea em menos de 50% dos casos, chegando em algumas casuísticas até 17,4%^145,146^ . Por esse motivo, na maioria dos casos é sugerida a coleta de material de forma asséptica, via cirúrgica ou via percutânea, a qual também apresenta resultados fidedignos^147^ . Infecções agudas de menor gravidade e sem tratamento anterior podem ser consideradas para tratamento empírico sem coleta de culturas, porém sugere-se realização ou repetição da mesma em face de qualquer evolução desfavorável ou quando a úlcera for submetida a desbridamento cirúrgico (classe de recomendação IIa, nível de evidência C)^11,13^ .

### Exames de imagem

A radiografia deve ser a modalidade de imagem inicial para avaliação de pacientes diabéticos com pé ulcerado e suspeita de osteomielite devido ao seu fácil acesso e baixo custo (classe de recomendação I, nível de evidência B)^142^ . Uma metanálise encontrou uma sensibilidade de 28% e uma especificidade de 68% para radiografias^143^ . Entretanto, quando essa modalidade é combinada com o *probe to bone test*, a sensibilidade e a especificidade aumentam para 97% e 93%, respectivamente^148^ . Assim, a combinação desses testes deve ser realizada para o diagnóstico inicial.

Alterações radiográficas são aparentes apenas quando ocorre 30-50% de perda óssea, podendo não ser visualizadas nos primeiros 10 dias da infecção^142^ . No Quadro 6 estão demonstrados parâmetros que podem ser encontrados na radiografia simples, associados a alterações de partes moles e osteomielite^149^ .

**Quadro 6:** Parâmetros radiográficos associados a alterações de partes moles e osteomielite.

Ø **Alterações de partes moles:**
• densidade anormal do tecido gorduroso;
• presença de gás;
• perda planos teciduais.
Ø **Alterações ósseas:**
• borramento ou perda da cortical óssea;
• erosões mal definidas e sequestro ósseo;
• reações periosteais;
esclerose óssea.

Fonte: Abikhzer et al.^142^ e Aragón-Sánchez et al.^149^ .

A tomografia computadorizada (TC) apresenta alta resolução espacial e fornece avaliação melhor das estruturas ósseas comparativamente à radiografia simples na avaliação de osteomielite. A TC também pode detectar gás e abscessos pequenos ou profundos que são imperceptíveis na radiografia, com sensibilidade de 67% e especificidade de 50% para o diagnóstico de osteomielite^142^ .

A ressonância magnética (RNM) continua a ser a principal modalidade anatômica para o diagnóstico de osteomielite em pacientes diabéticos (classe de recomendação I, nível de evidência B). A RNM também demonstra alterações no sinal da medula óssea, que podem se manifestar antes que a lise óssea se torne evidente na radiografia ou na TC^142^ . Apresenta uma sensibilidade de 93% e uma especificidade de 75% para o diagnóstico de osteomielite do pé diabético^150^ . A RM sem contraste de rotina com T1 sem supressão de gordura, T2 com supressão de gordura e sequências STIR em múltiplos planos ortogonais pode ser diagnóstica para osteomielite. Muitas vezes, o contraste é necessário e pode não ser viável em pacientes diabéticos com DRC^142^ .

A substituição da medula óssea por baixo sinal de T1 (mais escuro que o músculo esquelético) é tipicamente associada à osteomielite, que também pode estar associada à perda do sinal normal de T1 cortical devido à lise óssea ou à presença de edema periosteal, aumentando a confiança diagnóstica para osteomielite. O efeito “fantasma” das estruturas ósseas envolvidas na osteomielite também é um sinal útil. Os ossos são imperceptíveis nas sequências ponderadas em T1 pela substituição da medula e perda do córtex, tornando-se prontamente visíveis nas sequências sensíveis a fluídos (sequência T2 com saturação de gordura) ou realçadas por contraste. A substituição da medula óssea contígua a uma úlcera (com ou sem fístula) com edema de partes moles reforça o diagnóstico de osteomielite^142^ .

A tomografia por emissão de pósitrons com fluorodeoxiglicose e a cintilografia com leucócitos marcados podem também ser utilizadas para elucidação diagnóstica, porém, por apresentarem disponibilidade mais limitada e maior custo, devem ser reservadas para casos em que não foi possível chegar a uma conclusão com a avaliação inicial^142^ .

### Tratamento

O Fluxograma 5 resume o manejo da IPD. A terapia antibiótica empírica deve ser embasada em características de suscetibilidade local dos patógenos bem como levar em conta disponibilidade e interações medicamentosas possíveis. Recomenda-se o tratamento de patógenos virulentos, como *Staphylococcus aureus* e estreptococos beta-hemolíticos, considerando que patógenos menos virulentos tendem a apresentar-se apenas como contaminantes/colonizadores locais. Adicionalmente, as diretrizes do IWGDF recomendam a inclusão de um antibiótico que seja efetivo contra a *Pseudomonas aeruginosa* para todos os países de clima tropical, visto sua prevalência de isolamento em culturas realizadas, principalmente quando a lesão tem histórico de contato com meios úmidos^13^ .

**Fluxograma 5:** Abordagem de úlcera infectada do pé diabético.*O capítulo 4 estabelece as condutas para a úlcera de pé diabético na presença de doença arterial periférica. IPD = infecção do pé diabético; DAP = doença arterial periférica. Fonte: Conte et al.^14^ e Schaper et al.^13^ .

Em casos mais complexos, deve ser realizada interconsulta com infectologista, com discussão individualizada de cada caso^13^ . A Tabela 14 sugere combinações de terapia antibiótica empírica de acordo com a gravidade de apresentação da infecção. De acordo com opinião de especialistas, a terapia antibiótica para lesões simples pode ser realizada por 1 a 2 semanas. Pode-se considerar estender o prazo de tratamento até 4 semanas, principalmente em lesões extensas em cicatrização e casos em que haja associação de DAP de maior gravidade, comorbidade associada a retardo na cicatrização da ferida e aumento da taxa de falha no tratamento de infecções (classe de recomendação IIa, nível de evidência C)^151^ . Se houver ausência de resolução após esse período, o assistente deve considerar a possibilidade de falha e reavaliar o tratamento instituído até o momento^13,151^ .

**Tabela 14:** Sugestão de esquema antibiótico empírico para tratamento das infecções de pé diabético.

**Gravidade infecção**	**Fatores adicionais**	**Patógenos envolvidos**	**Sugestão terapia empírica**
**Leve**	Sem fatores	Gram positivo	PSRP, Cefalosporina de 1º geração. Em casos de alergia: Clindamicina, fluoroquinolonas, macrolídeo, doxiciclina.
	Uso de antibióticos recentes	Gram positivo e bacilos gram negativos	Betalactâmicos com inibidor de beta lactamase; S/T; Fluoroquinolonas.
	Risco para MRSA	MRSA	Linezolida, vancomicina, daptomicina.
**Moderada ou grave**	Sem fatores	Gram positivos e bacilos gram negativos	Betalactamicos com inibidor de beta lactamase.
	Uso de antibióticos recentes	Gram positivos e bacilos gram negativos	Betalactâmicos com inibidor de beta lactamase; Carbapênemicos; (solicitar avaliação de infectologista)
	Úlcera macerada ou clima quente	Gram positivos, bacilos gram negativos, Pseudomonas	Betalactâmicos com inibidor de beta lactamase; PSRP + ceftazidima; PSRP + ciprofloxacino; Carbapenêmicos.
	Membro isquêmico, necrose / gás	Gram positivos, Bacilos gram negativos, Anaeróbios	Betalactâmicos com inibidor de beta lactamase; Carbapênemicos; Cefalosporinas de 2º e 3º geração + clindamicina ou metronidazol.
	Fatores de risco para MRSA	MRSA	Considerar adicionar ou substituir por vancomicina, linezolida, daptomicina.
	Fatores de risco para resistência de bacilos Gram -	ESBL	Carbapenêmicos, fluoroquinolonas, aminoglicosídeos e colistina.

PSRP: penicilina semisintetica resistente à penicilinase; S/T: Sulfametoxazol + trimetoprim; ESBL: organismo produtor de Betalactamase; MRSA: *Staphyloccus aureus* resistente à meticilina. Fonte: Schaper et al.^13^ .

O uso de antibióticos tópicos na ferida ou uso indiscriminado de antibióticos sistêmicos/tópicos para prevenção de infecção em úlceras não infectadas não está indicado com o intuito de promover cicatrização. Em um contexto no qual a infecção de ferida não foi confirmada após avaliação pormenorizada, o uso de antibióticos não trouxe benefício e pode induzir resistência bacteriana, suplantando a possibilidade de benefício teórico nesse cenário (classe de recomendação III, nível de evidência B)^13^ .

Apesar de existirem casos de infecção leve, boa parte dos casos de IPDs necessitarão de intervenção cirúrgica para sua resolução. Em casos de infecção grave com necrose, presença de abscessos profundos, a avaliação cirúrgica é indispensável e a drenagem/descompressão deve ser idealmente realizada de urgência e na maioria dos casos em até 24 horas^11^ . Casos que envolvem urgência, em geral, estão associados à infecção de tecidos moles, sendo rara essa indicação em infecções ósseas isoladas. Em um primeiro momento, o procedimento cirúrgico deve envolver a ressecção de tecidos desvitalizados, infectados, redução da pressão em compartimentos profundos, atentando para manutenção de toda cobertura cutânea que seja viável, mesmo em posições não anatômicas, considerando a possibilidade do seu uso em cirurgias futuras para cobertura após controle infeccioso^152^ .

A despeito de o padrão-ouro para tratamento da osteomielite estar associado à ressecção do segmento ósseo envolvido, há descrição de taxas semelhantes de sucesso no tratamento da osteomielite sem ressecção óssea ou de ressecção óssea conservadora, principalmente quando limitada ao antepé, atingindo em algumas séries taxas de remissão de até 64% em 1 ano^153,154^ . O tempo sugerido de tratamento nesses casos é de 6 semanas, observando-se a evolução com melhora idealmente nas primeiras 2-4 semanas (classe de recomendação IIa, nível de evidência B). Caso não haja resolução nesse período, é sugerida a troca de abordagem com realização de biópsia para identificação do patógeno ou ressecção da estrutura envolvida^155^ .

Em casos nos quais foram realizadas ressecções completas da lesão óssea, a terapia antibiótica pôde ser reduzida para alguns dias após ressecção completa, principalmente quando coletados fragmentos de tecido ósseo da margem da ressecção cirúrgica que se mostraram livres de infecção, com culturas negativas. Em caso de culturas positivas da margem de ressecção intraoperatória, a recomendação se mantém em realizar o tratamento por 6 semanas (classe de recomendação IIb, nível de evidência C)^13^ .

De forma análoga às infecções graves, o tratamento da osteomielite pode ter sua terapia endovenosa modificada para via oral, com atenção à manutenção de um esquema antibiótico com cobertura semelhante e sugerindo-se a dosagem na faixa superior da dosagem terapêutica. O prolongamento além de 6 semanas de tratamento não mostrou benefício^88^ , e a consideração de manutenção de terapia supressiva antibiótica a longo prazo pelo IWGDF deve ser apenas para casos em que há grande quantidade de tecido necrótico ósseo que não seja passível de remoção ou material de síntese ortopédico infectado^155^ . Sugere-se seguimento com acompanhamento laboratorial reunindo dosagens seriadas de PCR, VHS e utilização de radiografias simples do pé, considerando-se a cura após 1 ano de acompanhamento^138^ .

Em relação a terapias adjuvantes para tratamento da infecção, até o momento não há recomendação de boa qualidade para utilização de oxigenioterapia hiperbárica ou tópica, fator estimulador de colônia de granulócitos, antissépticos tópicos^89^ , compostos com prata^90^ ou terapia por pressão negativa com intuito único de tratamento da infecção do pé diabético (classe de recomendação III, nível de evidência B)^13,156,157^ . Até o presente momento, os ensaios realizados para essas terapias adjuvantes são considerados de baixa qualidade e não apoiam substancialmente o seu uso, considerando os potenciais efeitos adversos e custos associados a essas terapias^13^ . A Tabela 15 resume as principais recomendações para o tratamento da IPD.

**Tabela 15:** Resumo das principais recomendações para o tratamento das infecções do pé diabético.

**Recomendações**	**Classe de recomendação e nível de evidência**
1. Avalie a presença de infecção baseado nos sinais e sintomas de inflamação local em todo paciente diabético e com úlcera nos pés.	Classe I/nível de evidência C^158-162^
2. Utilize a classificação da *Infectious Diseases Society of America* (IDSA) para estratificar a gravidade.	Classe I/nível de evidência B^90,163,164^
3. Utilize a dosagem de PCR, VHS ou procalcitonina em casos de dúvida diagnóstica.	Classe IIb/nível de evidência C^165-170^
4. Utilize a radiografia simples, teste de sondagem óssea e dosagem de velocidade de hemossedimentação para o diagnóstico de osteomielite.	Classe I/nível de evidência B^171-175^
5. Solicite RNM (preferencialmente) ou PET-TC /cintilografia com leucócitos marcados para diagnóstico da osteomielite apenas se houver dúvida diagnóstica após avaliação inicial.	Classe I/nível de evidência B^176-180^
6. Colete de culturas, de forma asséptica, para definição do patógeno envolvido em todas as úlceras de pé diabético infectadas.	Classe I/nível de evidência C^145,181-183^
7. Colete de culturas ósseas (cirúrgica ou percutânea) para identificação do patógeno em caso de osteomielite, principalmente quando houver falha do tratamento empírico ou alta probabilidade de osteomielite e dúvida diagnóstica após exames de imagem.	Classe IIa/nível de evidência C^145,181-183^
8. Utilize antibioticoterapia para tratamento das úlceras de pé diabético infectadas de acordo com o perfil de sensibilidade dos prováveis patógenos envolvidos, a gravidade da infecção e uso prévio de antibióticos.	Classe I/nível de evidência B^184-190^
9. Inicie antibioticoterapia parenteral em casos de infecção graves com possibilidade de modificação do tratamento para regime via oral quando melhora clínica e quando factível do ponto de vista de tolerância e biodisponibilidade.	Classe IIa/nível de evidência C^188-190^
10. Não utilize antibióticos tópicos para o tratamento de infecção de ferida.	Classe III/nível de evidência B^191,192^
11. Administre antibióticos por 1-2 semanas para o tratamento de infecção de partes moles e 3-4 semanas em lesões extensas em melhora e/ou coexistência de DAP grave que possa prolongar o período de cicatrização.	Classe IIa/nível de evidência C^189,190,193-195^
12. Administre antibióticos por até 6 semanas em casos de osteomielite, avaliando os resultados nas primeiras 2 a 4 semanas, considerando nova coleta ou adequação do tratamento de acordo com os resultados das culturas.	Classe IIa/nível de eviência B^196-198^
13. Administre antibióticos com espectro para gram-positivos e gram-negativos mais prevalentes nas lesões em caso de DAP, uso prévio de antibiótico, ou lesõess moderadas-graves. Adicione cobertura a anaeróbios estritos para os casos moderados a graves e considerar a associação para cobertura para *Pseudomonas aeruginosa.*	Classe IIa/nível de evidência C^189-195,199^
14. Reavalie e adeque a terapia antibiótica de acordo com os resultados de sensibilidade obtidos nas culturas coletadas e **não** utiliza antimicrobianos para feridas não infectadas com o objetivo de evitar infecção ou acelerar a cicatrização.	Classe I/nível de eviência C^200,201^
15. Considere reavaliar o tratamento e coletar novas culturas em caso de falha de tratamento após o tempo esperado.	Classe IIb/nível de evidência C^13^
16. O tratamento da osteomielite pode não envolver a ressecção cirúrgica do osso quando limitado ao antepé. Nos outros casos, considerar ressecção cirúrgica principalmente quando apresentar infecção de tecidos moles associados.	Classe IIb/nível de evidência B^201-207^
17. Durante a ressecção óssea cirúrgica sugerimos a coleta de um fragmento da porção óssea restante para realização de cultura e avaliação de infecção residual.	Classe IIb/nível de evidência C^208-211^
18. O tratamento antibiótico da osteomielite pode ser abreviado caso o foco de todo o osso seja retirado e a cultura do fragmento residual coletado seja negativa. Em caso de cultura positiva, seguir tratamento por 6 semanas.	Classe IIb/nível de evidência C^208-211^
19. Não utilize oxigenoterapia hiperbárica, oxigenoterapia tópica, uso rotineiro de antissépticos tópicos, preparações com prata, ou terapia por pressão negativa para tratamento de úlceras se a única indicação for para tratar a infecção.	Classe III/nível de evidência B^212-215^

Adaptado de Schaper et al.^13^ .

## CAPÍTULO 6. ARTROPATIA DE CHARCOT

### Introdução

A artropatia de Charcot é uma condição descrita desde 1868 e que permanece com sua fisiopatologia indefinida até o momento. Seu desenvolvimento está associado a condições que causam neuropatia dos membros inferiores, e o diabetes é sua principal causa^216^ . Sua incidência na população portadora de DM pode variar de 0,1-13%, chegando até 29% nos pacientes já portadores de neuropatia^217,218^ . As deformidades resultantes da artropatia de Charcot são causa de distribuição inadequada de pressão no pé, e, consequentemente, causa importante de úlceras dos pés nos pacientes diabéticos^217^ .

### Diagnóstico

Define-se a artropatia de Charcot como uma neuro-osteoartropatia não infecciosa dos ossos e articulações em pacientes com alterações de sensibilidade, levando à destruição da arquitetura do pé^219^ . Usualmente envolve o médiopé, retropé e tornozelo, e há dois mecanismos descritos para seu desenvolvimento^216,217^ .A teoria neurovascular explica o desenvolvimento pela disautonomia causada pela neuropatia com aumento da vascularização e estímulo da atividade osteoclástica como causa das deformidades; já a teoria neurotraumática explica o desenvolvimento por múltiplas lesões articulares e ósseas pela ausência da sensibilidade protetora e consequentes cicatrizações não adequadas das lesões com o desenvolvimento da artropatia^216,220^ .

Apesar de o diagnóstico precoce ser decisivo para a preservação do membro, é retardado por erro em até 79% dos casos, levando a atraso de até 7 meses no diagnóstico^217^ . Duas apresentações são possíveis, aguda e crônica, e seu diagnóstico ainda é essencialmente clínico. A forma aguda se apresenta com eritema, edema, dor e aumento da temperatura do pé, sendo frequentemente confundida com outras doenças, como celulite, gota, torções ou tromboses venosas profundas. A forma crônica é a mais característica, com perda do arco plantar do pé e lesão descrita como em “mata-borrão”.^221^ Em uma apresentação com edema, dor e eritema do pé sem lesões cutâneas evidenciáveis, a hipótese de artropatia de Charcot aguda deve sempre ser considerada (boa prática clínica).

Pode-se realizar a mensuração da temperatura infravermelho da pele na suspeita de artropatia de Charcot com pele íntegra nos locais de maior temperatura do pé ou tornozelo comparando com o membro contralateral no mesmo ponto anatômico (classe de recomendação IIb, nível de evidência C). Um aumento na temperatura da pele de 2° Celsius do pé em comparação com o mesmo local no pé contralateral tem sido usado como limiar para o diagnóstico de artropatia de Charcot ativa^222^ . Na ausência de outros sinais e sintomas de inflamação (ou seja, vermelhidão e inchaço), um aumento isolado na temperatura do pé pode nem sempre ser indicativo de CNO ativa e deve ser interpretado no contexto de outros achados clínicos^223,224^ . Embora seja parte essencial da avaliação diagnóstica, a elevação isolada da temperatura da pele do pé não é suficiente para diagnosticar ou descartar doença ativa. Consequentemente, a elevação assimétrica da temperatura é sensível, mas não específica, no diagnóstico de artropatia de Charcot ativa.

Idealmente, radiografias simples bilaterais devem ser realizadas, se possível, para fins de comparação em pacientes com diabetes e suspeita de artropatia de Chacot ativa (classe de recomendação IIa, nível de evidência B). O estudo radiográfico deve incluir projeções anteroposteriores (AP), oblíquas mediais e laterais em uma pessoa com diabetes mellitus e suspeita de neuroosteoartropatia de Charcot ativa. As visualizações do tornozelo e do pé devem incluir as projeções AP e lateral. Idealmente, radiografias em pé (também conhecidas como “sustentação de peso”) devem ser realizadas. Se um paciente não for capaz de suportar peso sobre os pés, as radiografias sem suporte de peso são uma alternativa, mas podem não demonstrar desalinhamentos que são mais aparentes na posição ortostática^225^ .

Caso a radiografia não apresente alterações, o diagnóstico não deve ser descartado, uma vez que estas alterações não são esperadas no início do processo^221^ . A RNM pode ser utilizada para o diagnóstico, porém suas alterações podem não ser diferenciáveis da osteomielite e seus achados devem ser correlacionados com a clínica, exames laboratoriais, biópsias e culturas ósseas^221^ . Se a ressonância magnética não está disponível ou é contraindicada, pode-se considerar estudo com cintilografia, tomografia computadorizada (TC) ou SPECT-CT (*Single Photon Emission Computerized Tomography*) para complementar o diagnóstico de neuro‐osteoartropatia de Charcot ativa^226,227^ .

As radiografias sem carga podem não apresentar alterações, e, por esse motivo, a radiografia deve sempre ser solicitada com carga. Caso a radiografia não apresente alterações, o diagnóstico não deve ser descartado, uma vez que as alterações não são esperadas no início do processo^95^ . A RNM pode ser utilizada para o diagnóstico, porém suas alterações podem não ser diferenciáveis da osteomielite e seus achados devem ser correlacionados com a clínica, exames laboratoriais, biópsias e culturas ósseas^95^ .

Marcadores bioquímicos em geral não estão alterados na artropatia de Charcot, porém as dosagens de PCR e VHS podem ser parâmetros utilizados para diagnóstico diferencial de uma lesão por infecção^216,217,219^ . Classicamente, a artropatia de Charcot aguda foi dividida por Eichenholtz em quatro estágios, conforme descrito na Tabela 16^218^ .

**Tabela 16:** Classificação de Eichenholtz modificada.

**Estágio**	**Radiografia**	**Clínica**	**Tratamento sugerido**
**0 (pré charcot – prodrômico)**	Normal.	Edema, eritema, calor.	Educação ao paciente, radiografias seriadas, descarga com proteção do membro.
**1 (desenvolvimento)**	Osteopenia, fragmentação, subluxações ou luxações de articulações.	Edema, eritema, calor, alterações ligamentares.	Descarga com proteção e uso de gesso de contato total ou órtese removível até resolução radiográfica dos fragmentos e redução clínica da temperatura.
**2 (coalescência)**	Absorção de debris, esclerose, fusão de fragmentos grandes.	Redução do edema, eritema e calor.	Uso de gesso de contato total ou outras órteses de proteção para descarga.
**3 (reconstrução)**	Consolidação da deformidade, anquilose fibrosa, fragmentos ósseos com bordas arredondadas e suaves.	Ausência de edema, eritema, calor, estabilidade da articulação e deformidade fixada.	Calçados adaptados para prevenção de úlceras, considerar encaminhamento ao cirurgião ortopédico para avaliação de correção de deformidades.

Fonte: Adaptado de Rosenbaum et al.^228^ .

### Tratamento

Pacientes com artropatia de Charcot devem ser referenciados a um time multidisciplinar para acompanhamento e cuidado. O tratamento inicial baseia-se na retirada da carga no membro e no uso de gesso de contato total, que tem sido realizado com sucesso para tratamento da fase aguda^229^ . A imobilização/descarga na altura do joelho deve ser iniciada imediatamente quando houver suspeita de artropatia de Charcot ativa em uma pessoa com diabetes mellitus e pele intacta (classe de recomendação IIa, nível de evidência C). Demonstrou-se que a detecção precoce, a imobilização e a redução da carga sobre o pé doente minimizam o desenvolvimento da deformidade^230,231^ . Seu uso deve ser realizado por tempo suficiente até a remissão dos sintomas, e os pacientes devem ser acompanhados com radiografias simples seriadas e exame clínico do membro. A diferença de temperatura cutânea entre os membros inferior a 2 °C é relacionada à resolução do processo bem como a consolidação das alterações ósseas nas radiografias^217,229^ .

Deve se atentar às contraindicações ao uso de gesso de contato total nos pacientes portadores de úlceras com infecções ativas. A monitorização semanal deve ser realizada com os pacientes em tratamento. Após remissão da fase inicial, estão indicados os calçados adaptados para prevenir recorrência, ulcerações e deformidades (classe de recomendação IIa, nível de evidência C)^217^ .

O tratamento da fase crônica da artropatia de Charcot pode envolver, além do uso de calçados adaptados, a abordagem cirúrgica. O encaminhamento desses pacientes a um time multidisciplinar com cirurgião ortopédico deve ser considerado para avaliação de possíveis indicações cirúrgicas, no intuito de prevenir ulcerações ou recidivas da doença^232,233^ . O Fluxograma 6 sugere a abordagem dos pacientes diabéticos com suspeita de artropatia de Charcot, e a Tabela 17 resume as principais recomendações.

**Fluxograma 6:** Fluxogama proposto para abordagem da artropatia de Charcot. Fonte: Adaptado de *Nothern* Diabetes Foot Care Network – Active Charcot Foot Guidelines^233^ .

**Tabela 17:** Recomendações para a artropatia de Charcot.

**Recomendações**	**Classe de recomendação e nível de evidência**
1. Atentar a pacientes diabéticos com fratura dos pés considerando que podem evoluir para artropatia de Charcot.	Boa prática clínica^216,217,220^
2. Sempre considere a possibilidade de artropatia de Charcot aguda como diagnóstico diferencial em pacientes diabéticos com neuropatia periférica que se apresentam com edema, eritema, aumento de temperatura no pé (em relação ao contra lateral) mesmo na ausência de dor.	Boa prática clínica^217,221^
3. Considere mensuração da temperatura infravermelho da pele na suspeita de artropatia de Charcot com pele íntegra nos locais de maior temperatura do pé ou tornozelo comparando com o membro contralateral no mesmo ponto anatómico.	Classe IIb/nível de evidência C^222-224^
4. Para diagnóstico da artropatia de Charcot sugerimos realização de radiografia com carga e se mantida a suspeita com radiografia normal, considerar realizar ressonância magnética nuclear.	Classe IIa/nível de evidência B^225,234-236^
5. Sugerimos não usar proteína C reativa (PCR), velocidade de hemossedimentação (VHS), hemograma, fosfatase alcalina ou outros exames de sangue em uma pessoa com diabetes mellitus e suspeita de neuroosteoartropatia de Charcot ativa com pele intacta para diagnosticar ou excluir a doença.	Classe III/nível de evidência C^237-242^
6. Inicie imobilização da perna/descarga de peso na suspeita clínica de artropatia de Charcot enquanto realiza estudos para confirmação.	Classe I/nível de evidência C^229-231^
7. Recomendamos o tratamento com o uso dispositivos de descarga de peso preferencialmente não removíveis e caso não seja possível, uso de dispositivos removíveis.	Classe IIa/nível de evidência C^230,231^
8. Pacientes com artropatia de Charcot aguda devem ser seguidos clinicamente semanalmente quanto a temperatura do membro até sua redução (a uma diferença inferior a 2º) e com radiografias seriadas até que não haja novas alterações e consolidação de lesões ósseas.	Classe IIa/nível de evidência C^243,244^

Adaptado de *Nothern Diabetes Foot Care Network – Active Charcot Foot Guidelines* ^233^ .
